# A Unified Synthesis of Diazenes from Primary Amines Using a SuFEx/Electrochemistry Strategy

**DOI:** 10.1021/acs.orglett.4c02218

**Published:** 2024-09-03

**Authors:** Katarzyna Doktor, Julien C. Vantourout, Quentin Michaudel

**Affiliations:** † Department of Chemistry, Texas A&M University, College Station, Texas 77843, United States; ‡ Syngenta Crop Protection AG, Schaffauserstrasse, 4332, Stein, Switzerland

## Abstract

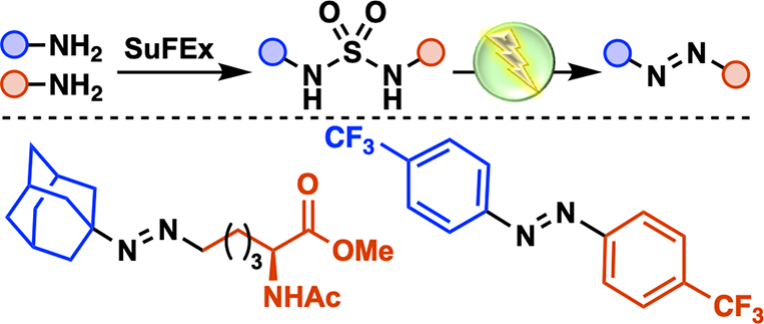

The electrochemical synthesis of 1,2-disubsituted diazenes via anodic oxidation of bench stable symmetrical and unsymmetrical sulfamides is reported. This work capitalizes on the streamlined preparation of diverse *N*,*N*′-disubstituted sulfamides using Sulfur(VI) Fluoride Exchange (SuFEx) click chemistry that were subsequently subjected to electrochemical oxidation to afford the desired diazenes. The electrochemical nature of the reaction conditions obviated the need for chlorinating reagents, which considerably improved the sustainability of the overall process. Noteworthy, in addition to the synthesis of alkyl diazenes, these milder conditions were shown to be competent for the formation of azobenzenes, albeit in lower yields. Mechanistic experiments were conducted to delineate the reaction pathway and to rationalize the formation of side products observed during the electro-oxidation of *N,N'*-diarylsulfamides.

Despite their known utility, universal synthetic methods to access organic 1,2-disubstituted diazenes (—N=N—) are scarce, in contrast to the numerous reactions available for the formation of alkenes. 1,2-Diaryldiazenes, also referred to as azo compounds, are found in a myriad of molecules from pharmaceuticals^1^ to photoresponsive materials^2^ including dyes, pigments, photochemical switches, and chemosensors (Scheme 1A) and are accessed through azo couplings and related reactions.^3−5^ 1,2-Dialkyldiazenes, on the other hand, have mostly been confined to the role of radical initiators, and only a handful of synthetic protocols have been reported to prepare such compounds. For example, azobis(isobutyronitrile) (AIBN), the staple of azo initiators, is routinely used in industrial processes including polymer manufacturing.^6^ While AIBN is synthesized from hydrazine, another common method toward 1,2-dialkyldiazenes is the oxidative transformation of sulfamides through the *aza*-Ramberg-Bäcklund reaction (Scheme 1B).^7^ Chlorination of a sulfamide derivative **A** under basic conditions leads to the formation of thiadiaziridine-1,l-dioxide **C** from chloro intermediate **B**.^8,9^ Loss of SO_2_ in turn generates the desired diazene **D**. First reported in 1965, this reaction has been seldomly used since and mostly for mechanistic studies relying on structurally simple diazenes.^10,11^ The requirement for strongly oxidizing reagents (e.g., NaOCl, trichloroisocyanuric acid (TCCA), or 1,3-dichloro-5,5-dimethylhydantoin) and an excess of strong bases (e.g., NaOH, DBU, BEMP)^7−15^ might explain the scarcity of reported 1,2-dialkyldiazene derivatives, as well as the lack of synthetic application for such compounds. A notable exception is the elegant pseudo dimerization strategy toward cyclotryptamine alkaloids reported by Movassaghi and co-workers that relied on the propensity of 1,2-dialkyldiazenes to undergo homolytic cleavage followed by radical recombination.^12−14^ More recently, 1,2-dialkyldiazenes were harnessed as radical precursors in photocatalyzed C—C cross-coupling reactions.^15−17^ These recent methods should motivate the development of efficient and modular processes to access diazene structures. A universal method to access both 1,2-dialkyl and 1,2-diaryldiazenes is especially desirable. However, the latter are known to resist the *aza*-Ramberg-Bäcklund reaction.^18^ The typical reaction conditions with aromatic substrates indeed lead to mixtures of oxidized compounds including arylbenzoquinone imine derivatives rather than the targeted azobenzene compound.^18^ In addition to being restricted to *N*,*N*′-dialkylsulfamides, few examples of unsymmetrical diazenes have been reported thus far.

**Scheme 1:**
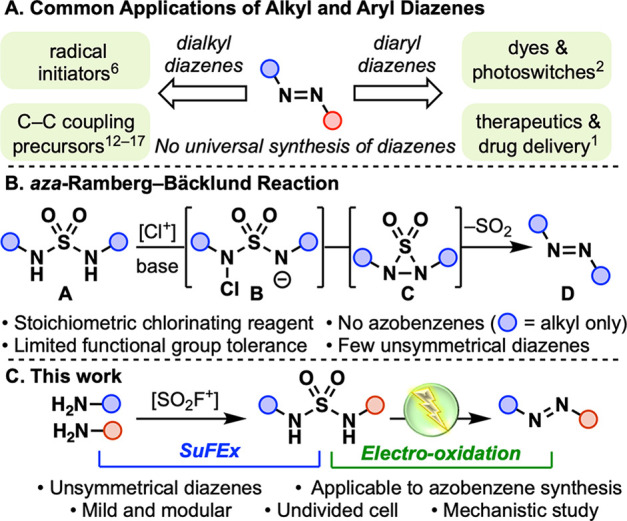
Synthesis and Applications of Diazenes

Herein, we report the electrochemical synthesis of diazenes via anodic oxidation of unsymmetrical sulfamides obtained with Sulfur(VI) Fluoride Exchange (SuFEx) click chemistry starting from readily available amines (Scheme 1C). The anodic oxidation obviated the need for chlorinating reagents, which considerably improved the sustainability^19−21^ of the overall process. It is noteworthy that, in addition to the synthesis of 1,2-dialkyldiazenes, these milder conditions were shown to be competent for the formation of 1,2-diaryldiazenes (azobenzenes) as well, albeit in lower yields.

The electrochemical oxidation of *N*,*N*′-dialkylsulfamides to afford the corresponding diazenes was first disclosed by Bauer and Wendt in 1978.^22^ While this report clearly demonstrated the feasibility of the transformation, the use of a divided cell^23−25^ and the scope being limited to only four substrates bearing no polar functional groups likely prohibited the broad adoption of this method.^26^ Inspired by the recent developments in electrochemical N–N bond formation by Baran,^27^ Waldvogel,^28,29^ and Stahl^30^ we set out to develop a more practical and general electrochemical variant of the *aza*-Ramberg-Bäcklund reaction. Coupled with our efforts toward the modular synthesis of sulfamides via SuFEx,^31−33^ this strategy should afford a streamline access to a large array of diazenes.

SuFEx provides milder reaction conditions and exceptional functional group tolerance in contrast to oxidative reagents like SO_2_Cl_2_.^34−36^ Capitalizing on an efficient one-pot protocol,^15^ a variety of *N*,*N*′-disubstituted sulfamides were isolated in good to high yields from a primary amine and 1-(fluorosulfonyl)-2,3-dimethyl-1*H*-imidazol-3-ium triflate (SuFEx-IT)^37^(Table 2 and Supporting Information). Delayed addition of DBU was observed to be crucial to avoid undesired side reactions during the formation of symmetrical sulfamides. Unsymmetrical sulfamides were obtained through isolation of the intermediate sulfamoyl fluoride by mixing a primary amine and SuFEX-IT, followed by addition of the second primary amines (Table 2 and Supporting Information).

**Table 1:**
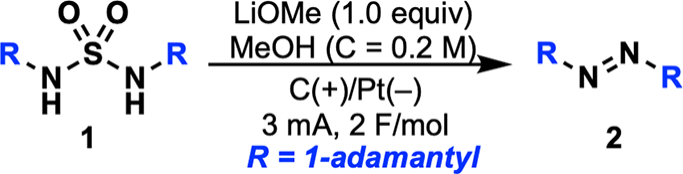
Optimization of the Anodic Oxidation of **1**

Entry	Deviations from above	**2**^a^	**1**^a^
1	None	14%	68%
2	Cs_2_CO_3_ (2.0 equiv)	21%	68%
3^b^	Cs_2_CO_3_ (2.0 equiv)	30%	63%
4^b^	LiCl and Cs_2_CO_3_	49%	48%
**5**^b^	**8 F/mol instead of 2 F/mol and LiCl, Cs**_**2**_**CO**_**3**_	**87%**^c^	**traces**
6^b^	Entry 5 but KPF_6_ instead of LiCl	51%	38%
7^b^	Entry 5 but C(−) instead of Pt(−)	78%	22%
8^b^	Entry 5 but no electricity	—	>99%

a Yields were calculated by ^1^H NMR using 1,2,4,5-tetramethylbenzene as the internal standard.

b MeOH [*C* = 0.04 M].

c Isolated yield.

**Table 2:**
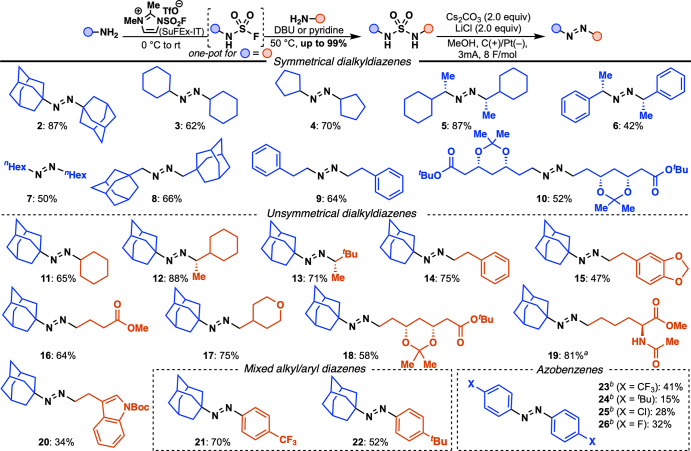
Scope of 1,2-Disubstituted Diazenes

a 6 F/mol instead of 8 F/mol.

b RVC(+) instead of C(+) and 6 F/mol instead of 8 F/mol.

We started our investigation of the electro-oxidation by adapting Wendt’s protocol^22^ to an undivided cell. Sulfamide **1** dissolved in MeOH was subjected to a constant current of 3 mA in the presence of LiOMe (1 equiv) using a graphite anode and a platinum foil cathode. Passing the charge of 2 F/mol resulted in a low 14% yield along with recovery of starting material (68%, Table 1, entry 1). Screening of bases revealed that inexpensive Cs_2_CO_3_ (2 equiv) slightly increased the yield compared to LiOMe (21%, Table 1, entry 2). Increase of the concentration of **1** enhanced the yield to 30% (Table 1, entry 3). LiCl proved to be the most efficient as a supporting electrolyte leading to a substantial increase of yield to 49% (Table 1, entry 4). Increasing the reaction charge to 8 F/mol delivered **2** in 87% isolated yield (Table 1, entry 5). Of note, substitution of the LiCl electrolyte by KPF_6_ provided **2** in 51% yield (Table 1, entry 6). This suggests that the mechanism may not involve a chlorination step, unlike the traditional *aza*-Ramberg-Bäcklund reaction. Interestingly, using a graphite electrode instead of a platinum cathode slightly reduced the efficiency but offered a less expensive alternative (Table 1, entry 7). In the absence of electricity, full recovery of **1** was observed (Table 1, entry 8).

With the optimized conditions in hand, several symmetrical and unsymmetrical sulfamides synthesized via SuFEx were tested. A variety of aliphatic diazenes were isolated in good to high yields including alicyclic compounds **2**–**5** and linear compound **7**. Notably, bulky α-tertiary compound **2** was tolerated, as well as α-secondary (**3**–**6**) and α-primary (**7**–**10**) sulfamides. With enantiopure sulfamides **5**, **6**, **12**, and **13**, only one stereoisomer was isolated as confirmed by NMR spectroscopy and measurement of their specific rotation.^38^ The electrochemical conditions delivered diazenes arising from benzylic and homobenzylic amines in moderate yields, as well as more challenging substrates containing *L-*lysine (**19**), Boc-protected tryptamine (**20**), or acetal (**10**, **15**, and **18**), ester (**10**, **16**, **18**, and **19**), and ether (**17**) groups. A variety of sulfamides bearing different substituents were prepared to showcase the potential of this two-step method to synthesize unsymmetrical diazenes (**11**–**22**) in high yields (up to 88%) including mixed alkyl-aryl diazenes **21** and **22**.

The successful isolation of diverse 1,2-dialkyldiazenes prompted us to revisit using the *aza*-Ramberg-Bäcklund transformation for azobenzene synthesis. We hypothesized that the mild electrochemical conditions might avoid the oxidative side reactions observed with chlorinating reagents.^18^ Applying the optimized conditions to *N*,*N*′-diarylsulfamides resulted in only trace amounts of the azo desired product; however, to our delight, switching to RVC(+) afforded moderate yields of azo compounds **23**–**26**. The presence of a *para*-substituent appeared to be a necessary condition as the reaction with *N*,*N*′-diphenylsulfamide did not deliver azobenzene. In addition, electron withdrawing groups through induction such as CF_3_, F, or Cl led to higher yields than the electron-rich *tert*-butyl group. Although yields are moderate, to the best of our knowledge, this is the first report of an *aza*-Ramberg-Bäcklund-like reaction to access azobenzenes.

To investigate the mechanism of this reaction, cyclic voltammetry (CV) experiments were conducted to compare the oxidation of dialkylsulfamide **1** and diarylsulfamidediarylsulfamide **27** (Figure 1A), in the presence and absence of Cs_2_CO_3_. Both substrates were characterized by a nonreversible oxidative cycle. Interestingly, **1** exhibited a higher oxidation potential than **27** suggesting that the lower yields obtained with diarylsulfamides are not caused by the initial oxidation step. Importantly, deprotonation with Cs_2_CO_3_ significantly decreased the oxidation potential of both substrates, from 2.00 to 1.48 V for **1** and from 1.76 to 1.05 V for **27** (vs Ag/AgCl). During the exploration of the reaction scope, anodic oxidation of unsymmetrical *N*,*N*′-diarylsulfamide **28** yielded only trace amounts of the desired product **29**. The major product was phenazine **30** (∼40%), confirmed by X-ray crystallography. (Figure 1B). Additionally, high-resolution mass spectrometry (HRMS) supported the formation of symmetrical azo compounds **23** and **24**, as well as phenazines **31** and **32**. Taken together, this data suggests a bimolecular mechanism for diaryl substrates involving a combination of two sulfamides followed by fragmentation. Delocalization of the nitrogen-centered radical over the aryl group putatively leads to the formation of the phenazine motif (see the postulated mechanism in Figure S4).^39^

**Figure 1:**
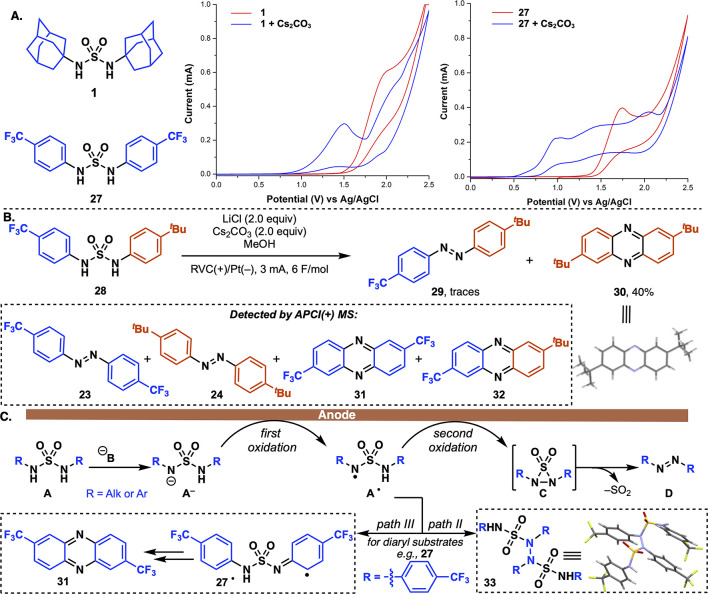
Mechanistic investigation. (A) Cyclic voltammetry of **1** and **27** (*C* = 10 mM) in MeOH with or without Cs_2_CO_3_ (2 equiv) using ^n^BuNPF_6_ (*C* = 100 mM), a glassy carbon electrode, a platinum wire auxiliary electrode, and a Ag/AgCl reference electrode. Scan rate of 100 mV/s. (B) Electro-oxidation of *N*,*N*′-diarylsulfamide **28**. (C) Proposed mechanism.

A proposed mechanism for the developed electrochemical *aza-*Ramberg-Bäcklund reaction is depicted in Figure 1C. Following deprotonation of **A**, single-electron oxidation produces sulfamidyl radical **A**^**•**^, a type of radical that has previously been generated photochemically.^40,41^ Indirect evidence for the formation of A^•^ was obtained through mass spectrometry, which detected dimer **33** as a minor side product arising from the electro-oxidation of **27**, consistent with previous electrochemical N–N coupling (path II).^42^ Replacing LiCl with KI as the supporting electrolyte led to a 33% yield of **33** and allowed for definitive structural identification through X-ray crystallography. The significant enhancement of the N–N coupling pathway with KI may be attributed to the *in situ* formation of iodine,^30^ underscoring the challenges in distinguishing between radical and polar mechanisms in this electrochemical oxidation. Subsequent deprotonation and oxidation, followed by cyclization, would generate **C**, the key intermediate^8,9^ of the *aza*-Ramberg-Bäcklund reaction en route to diazene **D**. Determining the exact pathway to C was complicated by side products, including **33**, which might contribute to diazene formation, and by potential oxidation of methoxide under these conditions (see Figures S5–S9). A coulometric experiment indicated that over 2 electrons were exchanged in the reaction (Supporting Information), which is consistent with the existence of competitive pathways and the required 8 F/mol to achieve full completion of the sulfamides. Of note, the oxidation potentials of deprotonated species **27**^**–**^ and **1**^**–**^ are 1.05 and 1.48 V (vs Ag/AgCl), respectively, and are thus lower than the first oxidation peak of LiCl (1.75 V vs Ag/AgCl) (Supporting Information). *In situ* generation of N–Cl bonds via LiCl oxidation is, therefore, unlikely the main pathway, which was confirmed by isolation of dialkyldiazene **2** in 51% yield when KPF_6_ was used instead of LiCl (Table 1, Entry 6).^43^ A diradical species akin to the one posited by Waldvogel for the electrochemical synthesis of pyrazolidin-3,5-diones could be at play,^28,29^ but ionic pathways cannot be ruled out yet. Finally, formation of phenazines (Figure S4) enabled by radical delocalization (pathway III) likely explains the lower yields observed with *N*,*N*′-diarylsulfamides.

In conclusion, an electrochemical oxidation of symmetrical and unsymmetrical *N*,*N*′-disubstituted sulfamides was developed. All sulfamides were efficiently obtained via SuFEx click chemistry and were stable on the benchtop. The reaction was shown to proceed under mild conditions allowing for the formation of a variety of diazenes including dialkyl, mixed alkyl/aryl, and diaryldiazenes (azobenzenes). A series of experiments provided insights into the potential reaction pathways occurring after anodic oxidation for various types of substrates.

## Data Availability

The data underlying this study are available in the published article and its Supporting Information.

## Supporting Information Available

Detailed experimental procedures, spectroscopic characterization, HRMS data, X-ray crystallographic data of compounds **30**, **31**, and **33**, and additional supporting data (PDF)
