# Supplementary material for: A Unified Synthesis of Diazenes from Primary Amines Using a SuFEx/Electrochemistry Strategy
Source: Org Lett. 2024 Sep 3;26(36):7501–6. doi: 10.1021/acs.orglett.4c02218 (PMC11406575; doi:10.1021/acs.orglett.4c02218)

A Unified Synthesis of Diazenes from Primary Amines Using a SuFEx/Electrochemistry Strategy

Katarzyna Doktor,[a] Julien C. Vantourout,[b]* and Quentin Michaudel,[a]*

[a]*Department of Chemistry, Texas A&M University, College Station, Texas 77843, United States*
[b]*Syngenta Crop Protection AG, Schaffauserstrasse, 4332, Stein, Switzerland*

Supporting Information

Experimental Procedures

General Reagent Information S2
General Analytical Information S2
Experimental procedures S3
Synthesis of sulfamoyl fluorides: **General Procedure A** S3
Synthesis and characterization of sulfamoyl fluorides S3
Synthesis of sulfamides: **General Procedure B** S4
Synthesis of sulfamides: **General Procedure C** S4
Synthesis and characterization of sulfamides S5
Synthesis of diazenes: **General Procedure D** S12
Synthesis of diazenes: **General Procedure E** S13
Synthesis and characterization of diazenes S13
Optimization of the synthesis of **23** S20
Isolation of phenazine **30** during the oxidation of *N,N'*-diarylsulfamide **28** S21
Oxidation of unsymmetrical sulfamide **S21** (aryl-alkyl) S22
Representative Synthesis of diazene **3** (1 mmol scale) S22
Characterization of isolated byproducts S23
Postulated mechanism for the formation of phenazines S24
Cyclic voltammetry (CV) S25
Coulometric experiment with **27** S27
X-ray crystallographic data S28
References S53
NMR Spectra S54

General reagent information

All reactions were performed without any precaution for moisture and oxygen unless otherwise stated. Dry acetonitrile (MeCN) and dichloromethane (DCM) was obtained by passing the previously degassed solvents through activated alumina columns. Anhydrous methanol (MeOH) was purchased from Sigma Aldrich and used without further purifications. All starting materials and reagents were purchased at the highest commercial quality and used without further purification, unless otherwise stated. Sulfuryl fluoride was purchased from SYNQUEST LABORATORIES. 1-(fluorosulfonyl)-2,3-dimethyl-1H-imidazol-3-ium triflate (SuFEx-IT)[1] was synthesized following a known procedure. The reactions were heated using an oil bath unless otherwise stated. Yields refer to chromatographically and spectroscopically (^{1}H NMR) homogeneous material, unless otherwise stated. Reactions were monitored by thin layer chromatography (TLC) carried out on 250 μm SiliCycle SilicaPlate™ silica plates (F254), using UV light as the visualizing agent and an acidic solution of p-anisaldehyde and heat, phosphomolybdic acid and heat, or ninhydrin and heat as developing agents. Flash silica gel chromatography was performed using SiliCycle SilicaFlash® Irregular Silica Gel (60 Å, particle size 40–63 μm). The electrochemical reactions were carried out using IKA ElectraSyn 2.0 equipped with the IKA carousel to perform several reactions simulatenously. All the electrodes (graphite, RVC, platinum foil) were purchased on the IKA website (https://www.ika.com/en/Products-LabEq/Electrochemistry-Kit-pg516/ElectraSyn-20-Package-20008980/Accessories-cpacc.html).

General analytical information

^{1}H Nuclear magnetic resonance (NMR) spectra were recorded on two Bruker Avance NEO 400 MHz and a Bruker Avance 500 MHz; ^{13}C spectra were recorded on a Bruker Avance 500 MHz and a Bruker Avance NEO 400 MHz; ^{19}F spectra were recorded using a Bruker Avance NEO 500 MHz instrument. All ^{1}H and ^{13}C spectra were calibrated using residual deuterated solvent as an internal reference ($CDCl_3$ @ 7.26 ppm ^{1}H NMR, 77.16 ppm ^{13}C NMR; DMSO-d_6 @ 2.50 ppm ^{1}H NMR, 39.52 ppm ^{13}C NMR). The following abbreviations were used to explain NMR peak multiplicities: s = singlet, d = doublet, t = triplet, q = quartet, m = multiplet, br = broad. High resolution mass spectra (HRMS) were recorded on an Agilent LC/MSD TOF mass spectrometer by electrospray ionization time-of-flight (ESI-TOF) reflection experiments. Mass spectrometric analysis was performed on an Orbitrap Velos Pro (Thermo Fisher Scientific) mass spectrometer. The sample was ionized using a homemade wire-in-a-capillary nanoelectrospray ionization source (nanoESI) with a spray voltage of 1.5 kV. The following MS parameters were used for data acquisition: S-lens RF level was set to 67.9%, and the capillary temperature was set at 280 °C. Full MS scans were acquired at *m/z* 110-600 at a resolving power of 60,000. A maximum injection time of 500 ms and 1 microscan were used for full MS scans. Cyclic voltammetry (CV) measurements were performed using an EG & G-Princeton Applied Research 263A all-in-one potentiostat-galvanostat, using a standard three-electrode setup with a glassy carbon electrode (working electrode, diameter = 3 mm), platinum wire auxiliary electrode and a Ag/AgCl as the reference electrode (4 M KCl). The Powersuite software was used for CV experiments. Data were saved using Microsoft Excel and treated using the Origin Pro 8.5 software.

Experimental procedures

Synthesis of sulfamoyl fluorides: **General Procedure A** (2.1 mmol scale is provided as an example)

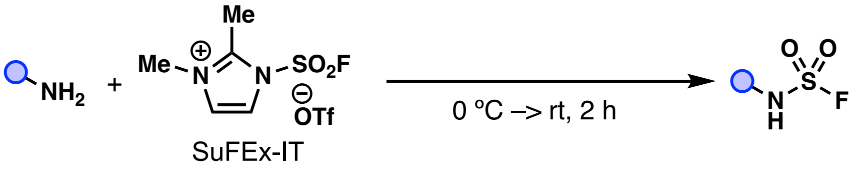

To a flame-dried 100 mL round-bottom-flask equipped with a PTFE-coated stir bar was added the starting amine (1.0 equiv, 2.1 mmol) followed by anhydrous DCM (4 mL, C = 0.25 M). The mixture was cooled to 0 °C with an ice-bath, and SuFEx-IT (328 mg, 1.0 equiv, 1.0 mmol) was added quickly to the solution in one portion. The reaction mixture was allowed to reach room temperature (~5–10 minutes) and then stirred for 2 hours. Upon completion, the reaction was quenched by the addition of aq. HCl (C = 1 M, ~20 mL). The mixture was extracted with DCM (~3×20 mL), and the combined organic layers were washed with brine (~20 mL), dried over $MgSO_4$ and then filtered. The solvent was evaporated *in vacuo* and the crude material was purified by column chromatography to afford the desired product.

Synthesis and characterization of sulfamoyl fluorides

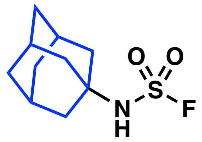

S1

S1 was prepared from 1-adamantylamine (1.0 g, 6.6 mmol) following **general procedure A**. Column chromatography (SiO_2, DCM) afforded **S1** as a white solid (1.1 g, 70%).

R_f = 0.28 (20:80 EtOAc:hexanes)
^{1}H NMR (500 MHz, $CDCl_3$) δ: 4.73 (s, 1 H), 2.19–2.12 (m, 3 H), 1.98 (d, J = 3.1 Hz, 6 H), 1.78–1.61 (m, 6 H) ppm.
^{13}C NMR (126 MHz, $CDCl_3$) δ: 57.1 (d, J = 2.6 Hz), 42.4, 35.8, 29.7 ppm.
^{19}F NMR (470 MHz, $CDCl_3$) δ: 60.9 (d, J = 5.0 Hz) ppm.
HRMS(–APCI) calc'd for $C_{10}H_{16}FNO_2S$ $[M–H]^-$ 232.0802, found 232.0806.

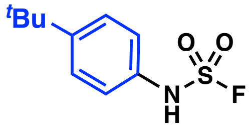

S2

S2 was prepared from 4-tert-butylaniline (746 mg, 5.0 mmol) following **general procedure A**. Column chromatography (SiO_2, 2:98 EtOAc:hexanes) afforded **S2** as a pink solid (860 mg, 74%).

R_f = 0.55 (10:90 EtOAc:hexanes)
^{1}H NMR (500 MHz, $CDCl_3$) δ: 7.45 – 7.41 (m, 2 H), 7.24 – 7.20 (m, 2 H), 6.67 (br, 1 H), 1.32 (s, 9 H) ppm.
^{13}C NMR (126 MHz, $CDCl_3$) δ: 151.9, 127.4, 126.7, 123.1, 34.6, 31.3 ppm.
^{19}F NMR (470 MHz, $CDCl_3$) δ: 50.7 (d, J = 4.9 Hz) ppm.
HRMS(–ESI) calc'd for $C_{10}H_{14}FNO_2S$ $[M–H]^-$ 230.0646, found 230.0652.

Synthesis of sulfamides: **General Procedure B** (2.1 mmol scale is provided as an example)

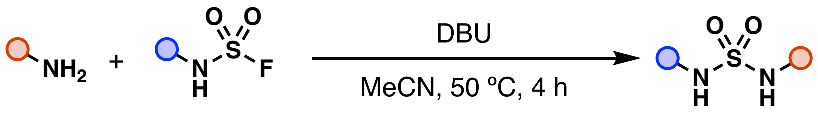

To a flame-dried 100 mL round-bottom-flask equipped with a PTFE-coated stir bar was added the starting sulfamoyl fluoride (1.0 equiv, 2.1 mmol) under argon, followed by amine (1.0 equiv, 2.1 mmol), and anhydrous MeCN (4 mL, C = 0.25 M). 1,8-diazabicyclo(5.4.0)undec-7-ene (DBU, 0.15 mL, 1.0 mmol, 1.0 equiv) was subsequently added dropwise over 1 minute and then the reaction mixture was stirred at 50 °C. The progression of the reaction was monitored by TLC. Upon completion, the reaction was quenched by the addition of aq. HCl (C = 1 M, ~20 mL). The mixture was extracted with EtOAc (~3×20 mL), and the combined organic layers were washed with brine (~20 mL), dried over $MgSO_4$ and then filtered. The solvent was evaporated *in vacuo* and the crude material was purified by column chromatography to afford the desired product.

Synthesis of sulfamides: **General Procedure C** (2.1 mmol scale is provided as an example)

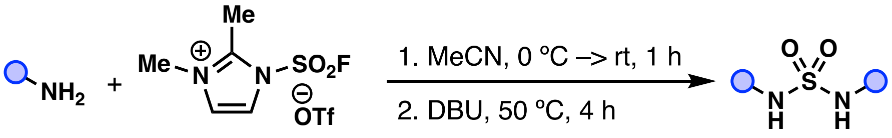

To a flame-dried 100 mL round-bottom-flask equipped with a PTFE-coated stir bar was added the starting amine (2.1 equiv, 2.1 mmol) under argon, followed by anhydrous MeCN (4 mL, C = 0.25 M). The mixture was cooled to 0 °C with an ice-bath, and SuFEx-IT (328 mg, 1.0 equiv, 1.0 mmol) was added quickly to the solution in one portion. The reaction mixture was allowed to reach room temperature (~5–10 minutes) and then stirred for 1 hour. 1,8-diazabicyclo(5.4.0)undec-7-ene (DBU, 0.15 mL, 1.0 mmol, 1.0 equiv) was subsequently added dropwise over 1 minute and then the reaction mixture was stirred at 50 °C. The progression of the reaction was monitored by TLC. Upon completion, the reaction was quenched by the addition of aq. HCl (C = 1 M, ~20 mL). The mixture was extracted with EtOAc (~3×20 mL), and the combined organic layers were washed with brine (~20 mL), dried over $MgSO_4$ and then filtered. The solvent was evaporated *in vacuo* and the crude material was purified by column chromatography to afford the desired product.

Synthesis and characterization of sulfamides

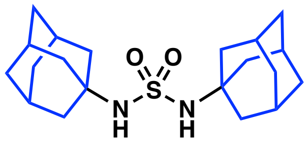

1

1 was prepared from **S1** (430 mg, 1.84 mmol) and 1-adamantylamine (279 mg, 1.84 mmol) following **general procedure B** without further purification. **1** was isolated as a white solid (540 mg, 80%).

R_f = 0.53 (20:80 EtOAc:hexanes)
^{1}H NMR (500 MHz, $CDCl_3$) δ: 3.95 (d, *J* = 5.6 Hz, 2 H), 2.12–2.07 (m, 6 H), 1.97 (d, *J* = 3.0 Hz, 12 H), 1.69–1.62 (m, 12 H) ppm.
^{13}C NMR (126 MHz, $CDCl_3$) δ: 54.8, 43.2, 36.2, 29.7 ppm.
HRMS(+ESI) calc'd for $C_{20}H_{32}N_2O_2S$ $[M+H]^+$ 365.2257, found 365.2246.

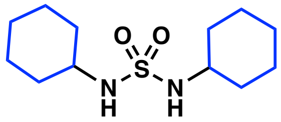

S3

S3 was prepared from SuFEx-IT (1.06 g, 3.2 mmol) and cyclohexylamine (500 mg, 6.8 mmol) following **general procedure C**. Column chromatography (SiO_2, 20:80 EtOAc:hexanes) afforded **S3** as a white solid (620 mg, 73%).

The spectroscopic data for this compound were identical to those reported in the literature (*Macromol. Rapid Commun.* **2006** *,27,* 976–981).

^{1}H NMR (400 MHz, $CDCl_3$) δ: 3.99 (d, *J* = 7.8 Hz, 2 H), 3.22–3.12 (m, 2 H), 2.07–1.94 (m, 4 H), 1.72–1.67 (m, 4 H), 1.64–1.55 (m, 2 H), 1.40–1.11 (m, 10 H) ppm.

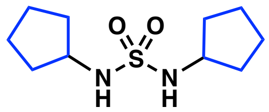

S4

S4 was prepared from SuFEx-IT (1.5 g, 4.6 mmol) and cyclopentylamine (0.95 mL, 9.6 mmol) following **general procedure C**. Column chromatography (SiO_2, 20:80 EtOAc:hexanes) afforded **S4** as a white solid (952 mg, 90%).

The spectroscopic data for this compound were identical to those reported in the literature (*Macromol. Rapid Commun.* **2006**, *27,* 976–981).

^{1}H NMR ($CDCl_3$) δ: 4.08–4.01 (m, 2 H), 3.67–3.60 (m, 2 H), 1.93 (br, 4 H), 1.65–1.57 (m, 4 H), 1.54–1.49 (m, 4 H), 1.47–1.41 (m, 4 H) ppm.

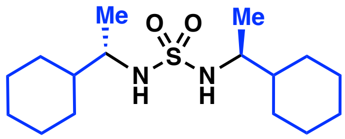

S5

S5 was prepared from SuFEx-IT (614 mg, 1.9 mmol) and (*S*)-(−)-1-cyclohexylethylamine (500 mg, 3.9 mmol) following **general procedure C**. Column chromatography (SiO_2, 20:80 EtOAc:hexanes) afforded **S5** as a white solid (422 mg, 71%)

R_f= 0.60 (20:80 EtOAc:hexanes)
^{1}H NMR (500 MHz, $CDCl_3$) δ: 4.01 (d, *J* = 8.8 Hz, 2 H), 3.30–3.23 (m, 2 H), 1.78–1.72 (m, 6 H), 1.69–1.64 (m, 4 H), 1.46–1.40 (m, 2 H), 1.27–1.19 (m, 4 H), 1.17 (d, *J* = 6.8 Hz, 6 H), 1.14–0.94 (m, 6 H) ppm.
^{13}C NMR (126 MHz, $CDCl_3$) δ: 54.6, 43.6, 29.4, 28.2, 26.6, 26.5, 26.3, 18.4 ppm.
HRMS(+ESI) calc'd for $C_{16}H_{32}N_2O_2S$ $[M+H]^+$ 317.2257, found 317.2247.

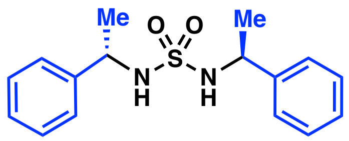

S6

S6 was prepared from SuFEx-IT (328 mg, 1.0 mmol) and (*S*)-(–)-1-phenylethylamine (318 mg, 2.1 mmol) following **general procedure C**. Column chromatography (SiO_2, 10:90 to 15:85 EtOAc:hexanes) afforded **S6** as a white solid (272 mg, 90%).

The spectroscopic data for this compound were identical to those reported in the literature (*J. Org. Chem.* **1984**, *49*, 3861–3862).

^{1}H NMR ($CDCl_3$, 400 MHz) δ: 7.64–7.50 (m, 10 H), 4.89–4.80 (m, 2 H), 1.79–1.71 (m, 2 H), 1.58–1.47 (m, 6 H) ppm.

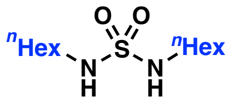

S7

S7 was prepared from from SuFEx-IT (765 mg, 2.3 mmol) and hexylamine (500 mg, 4.9 mmol) following **general procedure C**. Column chromatography (SiO_2, 20:80 EtOAc:hexanes) afforded **S7** as a white solid (440 mg, 99%).

The spectroscopic data for this compound were identical to those reported in the literature (*Polym. Adv. Technol.* **2011***, 22* 1529–1538).

^{1}H NMR (400 MHz, $CDCl_3$) δ 4.03 (t, *J* = 6.1 Hz, 2 H), 3.04 (q, *J* = 6.8 Hz, 4 H), 1.61–1.49 (m, 4 H), 1.41–1.22 (m, 12 H), 0.93–0.85 (m, 6 H) ppm.

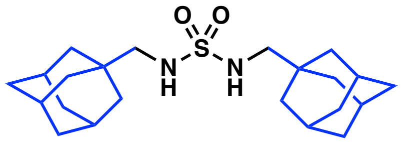

S8

S8 was prepared from SuFEx-IT (236 mg, 0.72 mmol) and 1-adamantanemethylamine (250 mg, 1.51 mmol) following **general procedure C**. Recrystallization from DCM at –20 ºC afforded **S8** as a white solid (165 mg, 58%).

R_f = 0.44 (20:80 EtOAc:hexanes)
^{1}H NMR (500 MHz, $CDCl_3$) δ 4.07 (t, *J* = 6.8 Hz, 2 H), 2.68 (d, *J* = 6.6 Hz, 4 H), 2.04–2.00 (br, 6 H), 1.75–1.62 (m, 12 H), 1.51 (d, *J* = 2.9 Hz, 12 H) ppm.
^{13}C NMR (126 MHz, $CDCl_3$) δ 55.2, 40.4, 37.0, 33.1, 28.3 ppm.
HRMS(+ESI) calc'd for $C_{22}H_{36}N_2O_2S$ $[M+H]^+$ 393.2570, found 393.2560.

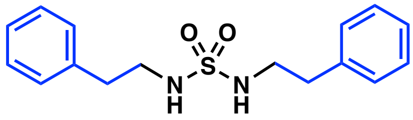

S9

S9 was prepared from SuFEx-IT (644 mg, 1.96 mmol) and 2-phenylethylamine (0.52 mL, 4.12 mmol) following **general procedure C**. Column chromatography (SiO_2, 20:80 EtOAc:hexanes) afforded **S9** as a white solid (512 mg, 86%)

The spectroscopic data for this compound were identical to those reported in the literature (*J. Am. Chem. Soc.* **2022**, *144*, 25, 11364–11376).

^{1}H NMR (400 MHz, $CDCl_3$) δ: 7.33–7.15 (m, 10 H), 4.02 (t, *J* = 6.5 Hz, 2 H), 3.22 (q, *J* = 6.7 Hz, 4 H), 2.81 (t, *J* = 6.8 Hz, 4 H) ppm.

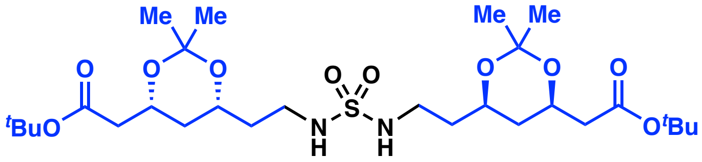

S10

S10 was prepared from SuFEx-IT (281 mg, 0.86 mmol) and 1,1-dimethylethyl (4*R*-*cis*)-6 aminoethyl-2,2-dimethyl-1,3-dioxane-4-acetate (500 mg, 1.83 mmol) following **general procedure C**. Column chromatography (SiO_2, 40:60 EtOAc:hexanes) afforded **S10** as a clear sticky solid (408 mg, 78%)

R_f = 0.33 (50:50 EtOAc:hexanes)
^{1}H NMR (500 MHz, $CDCl_3$) δ 4.77 (dd, *J* = 6.8, 4.7 Hz, 2 H), 4.30–4.21 (m, 2 H), 4.07 – 3.98 (m, 2 H), 3.23 – 3.07 (m, 4 H), 2.43 (dd, *J* = 15.2, 7.0 Hz, 2 H), 2.30 (dd, *J* = 15.2, 6.1 Hz, 2 H), 1.79–1.65 (m, 4 H), 1.54 (dt, *J* = 12.8, 2.5 Hz, 2 H), 1.45 (s, 6 H), 1.44 (s, 18 H), 1.37 (s, 6 H), 1.27 (dd, *J* = 18.5, 11.7 Hz, 2 H) ppm.
^{13}C NMR (126 MHz, $CDCl_3$) δ: 170.3, 99.0, 80.9, 68.8, 66.2, 42.7, 40.8, 36.3, 35.1, 30.3, 28.3, 19.9 ppm.
HRMS(+ESI) calc'd for $C_{28}H_{52}N_2O_{10}S$ $[M+NH_4]^+$ 626.3681, found 626.3659.

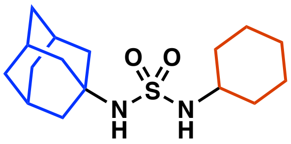

S11

S11 was prepared from **S1** (319 mg, 1.37 mmol) and cyclohexylamine (136 mg, 1.37 mmol) following **general procedure B**. Column chromatography (SiO_2, 20:80 EtOAc:hexanes) afforded **S11** as a white solid (392 mg, 92%)

R_f = 0.43 (20:80 EtOAc:hexanes)
^{1}H NMR (400 MHz, $CDCl_3$) δ: 3.99 (s, 1H), 3.97 (d, *J* = 7.5 Hz, 1H), 3.29–3.20 (m, 1 H), 2.10–2.08 (m, 3 H), 2.04 (dd, *J* = 12.5, 3.7 Hz, 2 H), 1.95 (d, *J* = 3.0 Hz, 6 H), 1.78–1.55 (m, 9 H), 1.41–1.11 (m, 5 H) ppm.
^{13}C NMR (126 MHz, $CDCl_3$) δ: 54.7, 53.0, 43.1, 36.2, 34.2, 29.7, 25.5, 25.0 ppm.
HRMS(+ESI) calc'd for $C_{16}H_{28}N_2O_2S$ $[M+H]^+$ 313.1944, found 313.1932.

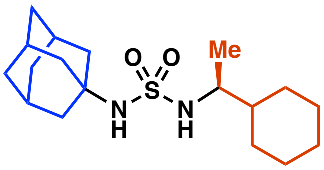

S12

S12 was prepared from **S1** (319 mg, 1.37 mmol) and (S)-(−)-1-cyclohexylethylamine (174 mg, 1.37 mmol) following **general procedure B**. Column chromatography (SiO_2, 10:90 EtOAc:hexanes) afforded **S12** as a white solid (428 mg, 92%)

R_f = 0.55 (20:80 EtOAc:hexanes)
^{1}H NMR ($CDCl_3$, 400 MHz) δ: 3.92 (s, 1H), 3.90 (d, *J* = 8.3 Hz, 1H), 3.55–3.28 (m, 1 H), 2.10–2.08 (m, 3 H), 1.96 (d, *J* = 3.0 Hz, 6 H), 1.78–1.72 (m, 3 H), 1.69–1.63 (m, 8 H), 1.48–1.41 (m, 1 H), 1.28–1.20 (m, 2 H), 1.18 (d, *J* = 6.8 Hz, 3 H), 1.15–0.94 (m, 3 H) ppm.
^{13}C NMR (126 MHz, $CDCl_3$) δ: 54.8, 54.7, 43.5, 43.1, 36.2, 29.8, 29.4, 28.3, 26.6, 26.5, 26.3, 18.2 ppm.
HRMS(+ESI) calc'd for $C_{18}H_{32}N_2O_2S$ $[M+H]^+$ 341.2257, found 341.2245.

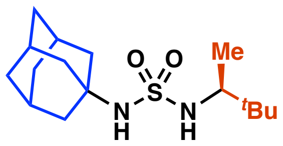

S13

S13 was prepared from **S1** (100 mg, 0.43 mmol) and (*S*)-(+)-3,3-dimethyl-2-butylamine hydrochloride (140 mg, 0.43 mmol) following **general procedure B** (*Note: 1.5 equiv. of DBU was added instead of 1.0 equiv.)* Column chromatography (SiO_2, 20:80 EtOAc:hexanes) afforded **S13** as a white solid (115 mg, 85%)

R_f = 0.62 (20:80 EtOAc:hexanes)
^{1}H NMR (500 MHz, $CDCl_3$) δ: 3.85 (s, 1 H), 3.79 (d, *J* = 9.3 Hz, 1 H), 3.20 (dq, *J* = 9.3, 6.7 Hz, 1 H), 2.11–2.09 (m, 3 H), 1.97 (d, *J* = 3.3 Hz, 6 H), 1.70–1.63 (m, 6 H), 1.23 (d, *J* = 6.7 Hz, 3 H), 0.92 (s, 9 H) ppm.
^{13}C NMR (126 MHz, $CDCl_3$) δ: 58.8, 55.0, 43.1, 36.2, 34.6, 29.8, 26.5, 17.0 ppm.
HRMS(+ESI) calc'd for $C_{16}H_{30}N_2O_2S$ $[M+H]^+$ 315.2101, found 315.2087.

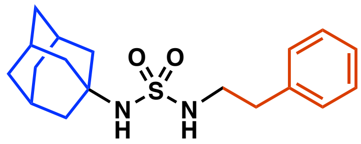

S14

S14 was prepared from **S1** (319 mg, 1.37 mmol) and 2-phenylethylamine (0.17 mL, 1.37 mmol) following general procedure B. Column chromatography (SiO_2, 20:80 EtOAc:hexanes) afforded **S14** as a white solid (398 mg, 87%)

R_f = 0.30 (20:80 EtOAc:hexanes)
^{1}H NMR (500 MHz, $CDCl_3$) δ: 7.33 (t, *J* = 7.5 Hz, 2 H), 7.23 (dd, *J* = 9.3, 7.7 Hz, 3 H), 3.96 (t, *J* = 6.2 Hz, 1 H), 3.91 (s, 1 H), 3.32 (q, *J* = 6.6 Hz, 2 H), 2.89 (t, *J* = 6.8 Hz, 2 H), 2.04–2.03 (m, 3 H), 1.82 (d, *J* = 2.9 Hz, 6 H), 1.66–1.57 (m, 6 H) ppm.
^{13}C NMR (126 MHz, $CDCl_3$) δ: 138.3, 129.0, 129.0, 127.0 54.5, 44.7, 42.8, 36.1, 35.7, 29.7 ppm.
HRMS(+ESI) calc'd for $C_{18}H_{26}N_2O_2S$ $[M+H]^+$ 335.1788, found 335.1775.

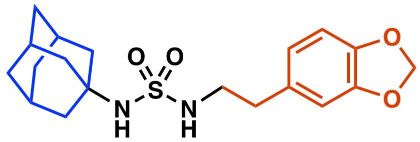

S15

S15 was prepared from **S1** (250 mg, 1.07 mmol) and 3,4-methylenedioxyphenethylamine (177 mg, 1.07 mmol) following **general procedure B**. Column chromatography (SiO_2, 20:80 EtOAc:hexanes) afforded **S15** as a white solid (320 mg, 72%)

R_f = 0.21 (20:80 EtOAc:hexanes)
^{1}H NMR (500 MHz, $CDCl_3$) δ: 6.76 (d, *J* = 7.8 Hz, 1 H), 6.70 (d, *J* = 1.7 Hz, 1 H), 6.66 (dd, *J* = 7.9, 1.7 Hz, 1 H), 5.94 (s, 2H), 3.98 (t, *J* = 6.7 Hz, 1H), 3.95 (s, 1 H), 3.26 (q, *J* = 6.6 Hz, 2 H), 2.80 (t, *J* = 6.7 Hz, 2 H), 2.05–2.04 (m, 3 H), 1.83 (d, *J* = 2.9 Hz, 6 H), 1.66–1.59 (m, 6 H) ppm.
^{13}C NMR (126 MHz, $CDCl_3$) δ: 148.2, 146.6, 132.0, 122.0, 109.3, 108.6, 101.2, 54.5, 44.8, 42.8, 36.1, 35.3, 29.6 ppm.
HRMS(+APCI) calc'd for $C_{19}H_{26}N_2O_4S$ $[M+H]^+$ 379.1686, found 379.1680.

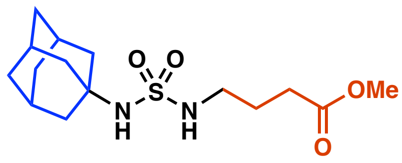

S16 was prepared from **S1** (319 mg, 1.37 mmol) and methyl 4-aminobutanoate (210 mg, 1.37 mmol) following **general procedure B**. Column chromatography (SiO_2, 30:70 EtOAc:hexanes) afforded **S16** as a white solid (518 mg, 77%)

R_f = 0.40 (50:50 EtOAc:hexanes)
^{1}H NMR (500 MHz, $CDCl_3$) δ: 4.27 (t, *J* = 6.3 Hz, 1 H), 4.08 (br, 1 H), 3.68 (s, 3 H), 3.11 (q, *J* = 6.7 Hz, 2 H), 2.43 (t, *J* = 7.1 Hz, 2 H), 2.10–2.09 (m, 3 H), 1.94–1.87 (m, 8 H), 1.69–1.63 (m, 6 H) ppm.
^{13}C NMR (126 MHz, $CDCl_3$) δ: 173.7, 54.6, 51.9, 43.0, 42.9, 36.2, 31.4, 29.7, 24.8 ppm.
HRMS(+ESI) calc'd for $C_{15}H_{26}N_2O_4S$ $[M+H]^+$ 331.1686, found 331.1674.

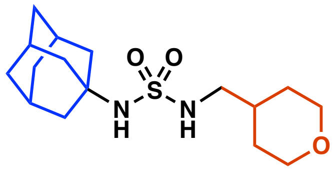

S17 was prepared from **S1** (319 mg, 1.37 mmol) and methyl 4-(aminomethyl)tetrahydropyran (158 mg, 1.37 mmol) following **general procedure B**. Column chromatography (SiO_2, 40:60 EtOAc:hexanes) afforded **S17** as a white solid (415 mg, 92%)

R_f = 0.22 (50:50 EtOAc:hexanes)
^{1}H NMR (400 MHz, CD_3CN) δ: 4.91 (t, *J* = 6.6 Hz, 1 H), 4.83 (s, 1 H), 3.90–3.85 (m, 2 H), 3.31 (td, *J* = 11.7, 2.1 Hz, 2 H), 2.82 (t, *J* = 6.6 Hz, 2 H), 2.06–2.04 (m, 3 H), 1.91 (d, *J* = 3.0 Hz, 6 H), 1.78–1.61 (m, 9 H), 1.27–1.16 (m, 2 H) ppm.
^{13}C NMR (126 MHz, CH_3CN) δ: 68.1, 54.7, 49.9, 43.4, 36.8, 36.0, 31.7, 30.6 ppm.
HRMS(+ESI) calc'd for $C_{16}H_{28}N_2O_3S$ $[M+H]^+$ 329.1893, found 329.1883.

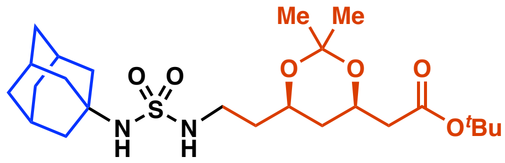

S18 was prepared from **S1** (319 mg, 1.37 mmol) and 1,1-dimethylethyl (4*R*-*cis*)-6-aminoethyl-2,2-dimethyl-1,3-dioxane-4-acetate (374.5 mg, 1.37 mmol) following **general procedure B**. Column chromatography (SiO_2, 20:80 EtOAc:hexanes) afforded **S18** as a yellow solid (518 mg, 77%)

R_f = 0.70 (50:50 EtOAc:hexanes)
^{1}H NMR (500 MHz, $CDCl_3$) δ: 4.72 (dd, *J* = 7.0, 4.8 Hz, 1 H), 4.28–4.23 (m, 1 H), 4.04–3.99 (m, 2 H), 3.23–3.09 (m, 2 H), 2.42 (dd, *J* = 15.2, 7.0 Hz, 1 H), 2.29 (dd, *J* = 15.2, 6.1 Hz, 1 H), 2.10–2.09 (m, 3 H), 1.94 (d, *J* = 2.9 Hz, 6 H), 1.75–1.62 (m, 8 H), 1.55 (dt, *J* = 12.8, 2.6 Hz, 1 H), 1.45 (s, 3 H), 1.44 (s, 9 H), 1.36 (s, 3H), 1.26 (dd, *J* = 12.9 Hz, *J* = 11.6 Hz, 1 H) ppm.
^{13}C NMR (126 MHz, $CDCl_3$) δ: 170.3, 99.0, 80.8, 68.7, 66.2, 54.4, 43.0, 42.7, 41.0, 36.3, 36.2, 35.1, 30.2, 29.7, 28.2, 19.9 ppm.
HRMS(+ESI) calc'd for $C_{24}H_{42}N_2O_6S$ $[M+NH_4]^+$ 504.3102, found 504.3083.

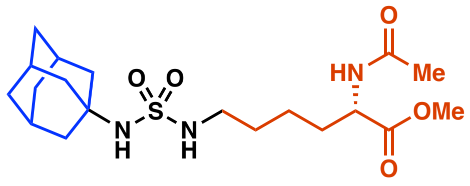

S19

S19 was prepared from **S1** (250 mg, 1.07 mmol) and N_α-acetyl-*L*-lysine methyl ester hydrochloride (220 mg, 1.07 mmol) following **general procedure B** (Note: 1.5 equiv. of DBU was added instead of 1.0 equiv.). Column chromatography (SiO_2, EtOAc) afforded **S19** as a white solid (320 mg, 72%)

R_f = 0.41 (EtOAc)
^{1}H NMR (500 MHz, $CDCl_3$) δ: 6.19 (d, *J* = 7.9 Hz, 1 H), 4.60 (td, *J* = 7.9, 5.1 Hz, 1 H), 4.31 (t, *J* = 6.2 Hz, 1 H), 4.18 (s, 1 H), 3.74 (s, 3 H), 3.03 (q, *J* = 6.7 Hz, 2 H), 2.10–2.08 (s, 3 H), 2.03 (s, 3 H), 1.94–1.91 (m, 6 H), 1.88–1.80 (m, 1 H), 1.71–1.53 (m, 9 H), 1.47–1.35 (m, 2 H) ppm.
^{13}C NMR (126 MHz, $CDCl_3$) δ: 173.2, 170.2, 54.5, 52.6, 52.0, 43.03, 42.95, 36.2, 32.1, 29.7, 29.0, 23.3, 22.4 ppm.
HRMS(+APCI) calc'd for $C_{19}H_{33}N_3O_5S$ $[M+H]^+$ 416.2214, found 416.2203.

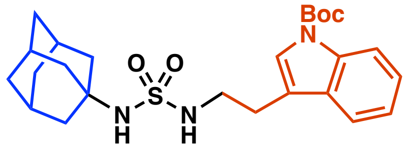

S20

S20 was prepared from **S1** (179 mg, 0.76 mmol) and 1-boc-tryptamine (200 mg, 0.76 mmol) following **general procedure B**. Column chromatography (SiO_2, 20:80 EtOAc:hexanes EtOAc) afforded **S20** as a white solid (277 mg, 77%)

R_f = 0.43 (20:80 EtOAc:hexanes)
^{1}H NMR (500 MHz, $CDCl_3$) δ: 8.14 (d, *J* = 8.3 Hz, 1 H), 7.54 (d, *J* = 7.7 Hz, 1 H), 7.46 (br, 1 H), 7.33 (t, *J* = 7.5 Hz, 1 H), 7.29 – 7.22 (m, 1 H *overlaps with CDCl₃*), 4.08 (t, *J* = 6.2 Hz, 1 H), 3.97 (br, 1 H), 3.39 (q, *J* = 6.6 Hz, 2 H), 3.00 (t, *J* = 6.6 Hz, 2 H), 1.98–1.96 (m, 3 H), 1.75 (d, *J* = 3.0 Hz, 6 H), 1.68 (s, 9 H), 1.61–1.51 (m, 6 H) ppm.
^{13}C NMR (126 MHz, $CDCl_3$) δ: 149.7, 135.9, 130.1 124.9, 123.9, 122.8, 118.9, 116.9, 115.7, 83.9, 54.5, 42.9, 42.7, 36.1, 29.6, 28.4, 25.2 ppm.
HRMS(+ESI) calc'd for $C_{25}H_{35}N_3O_4S$ $[M+H]^+$ 474.2421, found 474.2415.

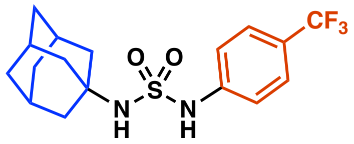

S21

S21 was prepared from **S1** (319 mg, 1.37 mmol) and 4-(trifluoromethyl)aniline (220 mg, 1.37 mmol) following **general procedure B**. Column chromatography (SiO_2, 20:80 EtOAc:hexanes) afforded **S21** as a white solid (445 mg, 87%)

R_f = 0.50 (20:80 EtOAc:hexanes)
^{1}H NMR (400 MHz, $CDCl_3$) δ: 7.52 (d, *J* = 8.5 Hz, 2 H), 7.12 (d, *J* = 8.4 Hz, 2 H), 6.46 (s, 1 H), 4.32 (s, 1 H), 2.02–1.99 (m, 3 H), 1.82 (d, *J* = 2.9 Hz, 6 H), 1.61–1.52 (m, 6 H) ppm.
^{13}C NMR (126 MHz, $CDCl_3$) δ: 140.9, 126.6 (q, *J* = 3.7 Hz), 125.3 (q, *J* = 33.1 Hz), 124.1 (q, *J* = 271.4 Hz), 117.4, 54.4, 42.6, 35.8, 29.5 ppm.
^{19}F NMR ($CDCl_3$, 470 MHz) δ: 62.0 ppm.
HRMS(–APCI) calc'd for $C_{17}H_{21}F_3N_2O_2S$ $[M–H]^-$ 373.1192, found 373.1196.

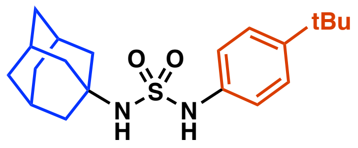

S22

S22 was prepared from **S1** (319 mg, 1.37 mmol) and 4-tert-butylaniline (204 mg, 1.37 mmol) following **general procedure B**. Column chromatography (SiO_2, 20:80 EtOAc:hexanes) afforded **S22** as a light brown solid (405 mg, 82%)

R_f = 0.48 (20:80 EtOAc:hexanes)
^{1}H NMR (400 MHz, $CDCl_3$) δ 7.33 (d, *J* = 8.7 Hz, 2 H), 7.09 (d, *J* = 8.7 Hz, 2 H), 6.23 (br, 1 H), 4.30 (br, 1 H), 2.10–2.05 (m, 3 H), 1.92 (d, *J* = 3.0 Hz, 6 H), 1.69 – 1.59 (m, 6 H), 1.30 (s, 9 H) ppm.
^{13}C NMR (126 MHz, $CDCl_3$) δ 147.4, 135.0, 126.4, 119.6, 55.1, 42.9, 36.1, 34.5, 31.5, 29.7 ppm.
HRMS(+ESI) calc'd for $C_{20}H_{30}N_2O_2S$ $[M+H]^+$ 363.2101, found 363.2099.

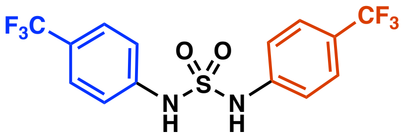

27

27 was prepared from SuFEx-IT (328 mg, 1.00 mmol) and 4-(trifluoromethyl)aniline (338 mg, 2.1 mmol) following **general procedure C** (*Note: pyridine was used instead of DBU).* Column chromatography (SiO_2, 20:80 EtOAc:hexanes) afforded **27** as a white solid (165 mg, 43%)

The spectroscopic data for this compound were identical to those reported in the literature (*Chem. Commun.* **2021**, *57*, 4775–4778).^{1}H NMR (400 MHz, DMSO-d_6) δ: 11.05 (br, 2 H), 7.63 (d, *J* = 8.5 Hz, 4 H), 7.31 (d, *J* = 8.5 Hz, 4 H) ppm.

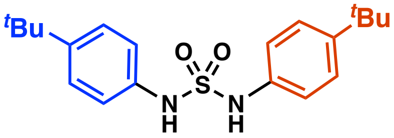

S23

S23 was prepared from **S2** (400 mg, 1.88 mmol) and 4-tert-butylaniline (280 mg, 1.88 mmol) following **general procedure B** *(Note: pyridine was used instead of DBU).* Column chromatography (SiO_2, 20:80 EtOAc:hexanes) afforded **S23** as a purple solid (662 mg, 97%).

The spectroscopic data for this compound were identical to those reported in the literature (*Chem. Commun.* **2021**, *57*, 4775–4778).

^{1}H NMR (400 MHz, $CDCl_3$) δ: 7.34–7.28 (m, 4 H), 7.06–7.01 (m, 4 H), 6.59 (br, 2 H), 1.29 (s, 18 H) ppm.

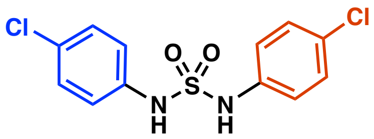

S24

S24 was prepared from SuFEx-IT (300 mg, 0.91 mmol) and 4-chloro- aniline (243 mg, 1.92 mmol) following **general procedure C** *(Note: pyridine was used instead of DBU).* Column chromatography (SiO_2, 20:80 EtOAc:hexanes) afforded **S24** as a yellow solid (141 mg, 49%).

The spectroscopic data for this compound were identical to those reported in the literature (*Chem. Commun.* **2021**, *57*, 4775–4778).

^{1}H NMR (400 MHz, DMSO-d_6) δ: 10.46 (br, 2 H), 7.42–7.26 (m, 4 H), 7.17–7.05 (m, 4 H) ppm.

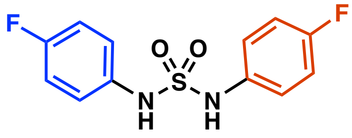
S25

S25 was prepared from SuFEx-IT (300 mg, 0.91 mmol) and 4-fluoro-aniline (212 mg, 1.92 mmol) following **general procedure C** *(Note: pyridine was used instead of DBU).* Column chromatography (SiO_2, 20:80 EtOAc:hexanes) afforded **S25** as a purple solid (106 mg, 41%).

The spectroscopic data for this compound were identical to those reported in the literature (*Chem. Commun.* **2021**, *57*, 4775–4778).

^{1}H NMR (400 MHz, $CDCl_3$) δ: 7.10–6.96 (m, 8 H), 6.60 (br, 2 H) ppm.

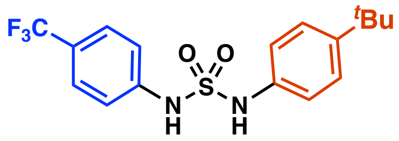
28

28 was prepared from **S2** (300 mg, 1.41 mmol) and 4-fluoro-aniline (227 mg, 1.41 mmol) following **general procedure C** *(Note: pyridine was used instead of DBU and the reaction was complete after 16 h).* Column chromatography (SiO_2, 5:95 to 20:80 EtOAc:hexanes) afforded **28** as an off-white solid (492 mg, 94%).

R_f = 0.19 (20:80 EtOAc:hexanes)
^{1}H NMR (400 MHz, $CDCl_3$) δ: 7.57 (d, *J* = 8.4 Hz, 2 H), 7.31 (d, *J* = 8.6 Hz, 2 H), 7.19 (d, *J* = 8.4 Hz, 2 H), 6.99 (d, *J* = 8.7 Hz, 2 H), 6.71 (s, 1 H), 6.50 (s, 1 H), 1.29 (s, 9 H) ppm.
^{13}C NMR (126 MHz, $CDCl_3$) 149.8, 139.9, 132.7, 126.8 (q, *J* = 3.8 Hz), 126. 7 (q, *J* = 33.2 Hz), 126.5, 123.9 (q, *J* = 271.5 Hz), 122.8, 119.3, 34.5, 31.7 ppm.
^{19}F NMR ($CDCl_3$, 470 MHz) δ: 62.2 ppm.
HRMS(–APCI) calc'd for $C_{17}H_{19}F_3N_2O_2S$ $[M–H]^-$ 371.1036, found 371.1043.

Synthesis of diazenes: **General Procedure D**

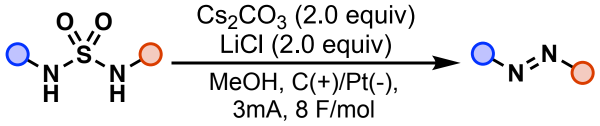

An oven dried ElectraSyn vial equipped with a magnetic stir bar was charged with solids, sulfamide (1.0 equiv, 0.30 mmol), Cs_2CO_3 (2.0 equiv, 0.60 mmol), and LiCl (2.0 equiv, 0.60 mmol). The ElectraSyn vial was then sealed with a cap equipped with selected anode (graphite) and cathode (Pt foil). MeOH (7.5 mL, C = 0.04 M). was then added, and stirring was applied for 20 min (or until the sulfamide was fully dissolved). The reaction mixture was electrolyzed under 3 mA constant current and with 6–8 F/mol total charge. The progression of the reaction was monitored by TLC. Upon completion, the solvent was removed under vacuo and the reaction mixture was directly purified by column chromatography to afford the desired product.

Synthesis of diazenes: **General Procedure E**

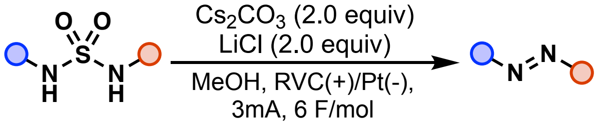

Three oven dried ElectraSyn vials were equipped with a magnetic stir bar and each vial was charged with solids, sulfamide (1.0 equiv, 0.10 mmol), Cs_2CO_3 (2.0 equiv, 0.20 mmol), and LiCl (2.0 equiv, 0.20 mmol). The ElectraSyn vials were then sealed with a cap equipped with anode (RVC) and cathode (Pt foil). MeOH (5 mL, C = 0.02 M) was then added, and stirring was applied for 20 min (or until the sulfamide was fully dissolved). The reaction mixtures were electrolyzed under 3 mA constant current and with 6 F/mol total charge. The solvent was then removed under vacuo and the reaction mixture of three reactions was directly purified by column chromatography to afford the desired product.

Note: While we have never observed any explosive or uncontrolled reactions with this procedure, using a blast shield is recommended because of the high reactivity of diazenes. All diazenes were stored at –20 °C in the dark. No decomposition was observed in these conditions over the course of several weeks. Slow decomposition was observed at room temperature over the course of several days or hours.

Synthesis and characterization of diazenes

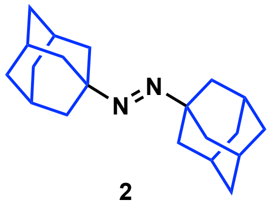

2

2 was prepared from **1** (109.5 mg, 0.3 mmol) following **general procedure D**. Column chromatography (SiO_2, 5:95 EtOAc:hexanes) afforded **2** as a white solid (78 mg, 87%).

The spectroscopic data for this compound were identical to those reported in the literature (*J. Am. Chem. Soc*. **1987**, *109*, 2750–2759).

^{1}H NMR (400 MHz, $CDCl_3$) δ: 2.13 (br, 6 H), 1.77–1.63 (m, 24 H) ppm.

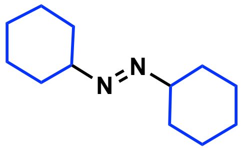

3

3 was prepared from **S3** (78.1 mg, 0.3 mmol) following **general procedure D**. Column chromatography (SiO_2, hexanes) afforded **3** as a yellow oil (36 mg, 62%).

The spectroscopic data for this compound were identical to those reported in the literature (*Macromol. Rapid Commun.* **2006** *,27,* 976–981).

^{1}H NMR (400 MHz, $CDCl_3$) δ: 3.31–3.20 (m, 2 H), 1.85–1.79 (m, 4 H), 1.74–1.60 (m, 10 H), 1.41–1.20 (m, 6 H) ppm.

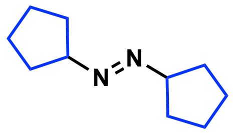

4 was prepared from **S4** (70 mg, 0.3 mmol) following **general procedure D**. Column chromatography (SiO_2, hexanes) afforded **4** as a colorless oil (35 mg, 70%). *Note: the solvent was removed under air.*

4

The spectroscopic data for this compound were identical to those reported in the literature (*Polym. Adv. Technol.* **2011**, *22* 1529–1538).

^{1}H NMR (400 MHz, $CDCl_3$) δ: 3.87–3.77 (m, 2 H), 1.87–1.66 (m, 16 H) ppm.

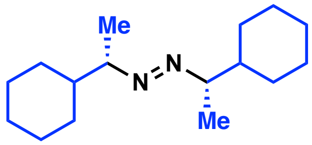

5 was prepared from **S5** (95 mg, 0.3 mmol) following **general procedure D**. Column chromatography (SiO_2, 5:95 EtOAc:hexanes) afforded **5** as a yellow oil (65 mg, 87%).

5

R_f = 0.78 (5:95 EtOAc:hexanes)
^{1}H NMR (500 MHz, $CDCl_3$) δ: 3.15 (p, *J* = 6.8 Hz, 2H), 1.82–1.62 (m, 12 H), 1.33–1.14 (m, 6 H), 1.11 (d, *J* = 6.8 Hz, 6 H), 1.05–0.91 (m, 4 H) ppm.
^{13}C NMR (126 MHz, $CDCl_3$) δ: 77.6, 42.1, 30.3, 29.5, 26.7, 26.4, 26.3, 16.2 ppm.
HRMS(+ESI) calc'd for $C_{16}H_{30}N_2$ $[M+H]^+$ 251.2482, found 251.2475.
Optical rotation: $[\alpha]_D^{23}$(c = 20 mg/mL, CCl_4) = –53°.

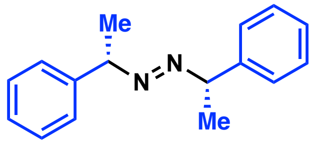

6 was prepared from **S6** (95 mg, 0.3 mmol) following **general procedure D**. Column chromatography (SiO_2, 5:95 EtOAc:hexanes) afforded **6** as a yellow oil (30 mg, 42%).

6

The spectroscopic data for this compound were identical to those reported in the literature (*ACS Cat.* **2023**, *13,* 7263–7268).

^{1}H NMR ($CDCl_3$, 400 MHz) δ: 7.39–7.31 (m, 8 H), 7.30–7.24 (m, 2 H, *overlaps with* $CDCl_3$), 4.65 (q, *J* = 6.8 Hz, 2 H), 1.57 (d, *J* = 6.8 Hz, 6 H) ppm.
Optical rotation: $[\alpha]_D^{23}$(c = 18 mg/mL, CCl_4) = –276°.

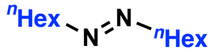

7 was prepared from **S7** (58 mg, 0.3 mmol) following **general procedure D**. Column chromatography (hexanes) afforded **7** as a colorless oil (19 mg, 50%). *Note: the solvent was removed under air.*

7

The spectroscopic data for this compound were identical to those reported in the literature (*Polym. Adv. Technol.* **2011**, *22* 1529–1538).

^{1}H NMR ($CDCl_3$, 400 MHz) δ: 3.65 (t, *J* = 7.2 Hz, 4 H), 1.72–1.65 (m, 4 H), 1.28–1.19 (m, 12 H), 0.81–0.72 (m, 6 H) ppm.

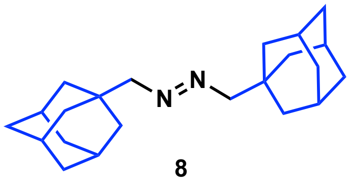

8

8 was prepared from **S8** (118 mg, 0.3 mmol) following **general procedure D**. Column chromatography (SiO_2, 5:95 EtOAc:hexanes) afforded **8** as a white solid (65 mg, 66%).

R_f = 0.75 (hexanes)
^{1}H NMR (400 MHz, $CDCl_3$) δ 3.41 (s, 4 H), 2.04–1.98 (m, 6 H), 1.76–1.68 (m, 12 H), 1.65 (d, J = 3.0 Hz, 12 H) ppm.
^{13}C NMR (126 MHz, $CDCl_3$) δ 81.7, 41.5, 37.2, 34.3, 28.7 ppm.
HRMS(+ESI) calc'd for $C_{22}H_{34}N_2$ $[M+H]^+$ 327.2795, found 327.2786.

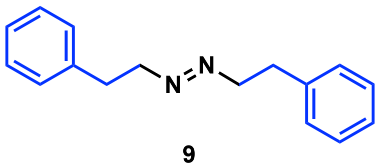

9

9 was prepared from **S9** (91 mg, 0.3 mmol) following **general procedure D**. Column chromatography (SiO_2, 5:95 EtOAc:hexanes) afforded **9** as a yellow solid (46 mg, 64%).

R_f = 0.65 (5:95 EtOAc:hexanes)
^{1}H NMR (400 MHz, $CDCl_3$) δ: 7.31–7.26 (m, 4 H, *overlaps with CDCl₃*), 7.23–7.18 (m, 6 H), 4.07 (t, J = 7.6 Hz, 4 H), 3.06 (t, J = 7.6 Hz, 4 H) ppm.
^{13}C NMR (126 MHz, $CDCl_3$) δ: 139.5, 129.0, 128.6, 126.4, 70.3, 34.0 ppm.
HRMS(+ESI) calc'd for $C_{16}H_{18}N_2$ $[M+H]^+$ 239.1543, found 239.1539.

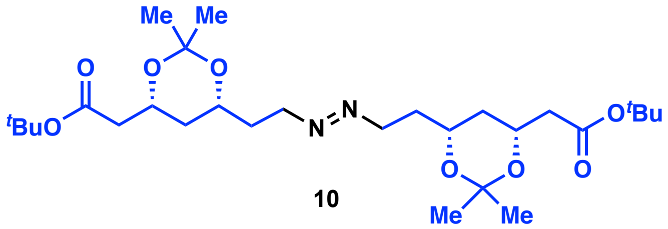

10

10 was prepared from **S10** (183 mg, 0.3 mmol) following **general procedure D**. Column chromatography (SiO_2, 5:95 to 30:80 EtOAc:hexanes) afforded **10** as a viscous oil (86 mg, 52%).

R_f = 0.55 (40:60 EtOAc:hexanes)
^{1}H NMR (500 MHz, $CDCl_3$) δ: 4.28–4.22 (m, 2 H), 3.97–3.92 (m, 2 H), 3.83 (hept, J = 6.6 Hz, 4 H), 2.43 (dd, J = 15.1, 7.0 Hz, 2 H), 2.30 (dd, J = 15.1, 6.1 Hz, 2 H), 1.98–1.85 (m, 4 H), 1.59 (dt, J = 12.7, 2.4 Hz, 2 H), 1.45 (s, 6 H), 1.42 (s, 18 H), 1.36 (s, 6 H), 1.24 (dd, J = 11.9, 11.8 Hz, 1H) ppm.
^{13}C NMR (126 MHz, $CDCl_3$) δ: 170.4, 98.9, 80.7, 67.0, 66.4, 65.1, 42.9, 36.7, 34.2, 30.2, 28.3, 19.9 ppm.
HRMS(+APCI) calc'd for $C_{28}H_{50}N_2O_8$ $[M+H]^+$ 543.3640, found 543.3632.
Optical rotation: $[\alpha]_D^{23}$(c = 10 mg/mL, CCl_4) = +13°.

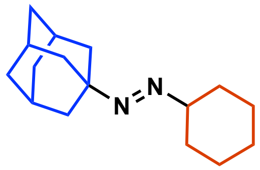

11

11 was prepared from **S11** (93.8 mg, 0.3 mmol) following **general procedure D**. Column chromatography (SiO_2, hexanes) afforded **11** as a yellow oil (48 mg, 65%).

R_f = 0.83 (hexanes)
^{1}H NMR (500 MHz, $CDCl_3$) δ: 3.30–3.24 (m, 1 H), 2.15–2.13 (m, 3 H), 1.87–1.82 (m, 2 H), 1.75–1.63 (m, 16 H), 1.40–1.30 (m, 4 H) ppm.
^{13}C NMR (126 MHz, $CDCl_3$) δ: 76.3, 66.9, 40.6, 36.8, 30.9, 29.5, 25.8, 24.5 ppm.
HRMS(+ESI) calc'd for $C_{16}H_{26}N_2$ $[M+H]^+$ 247.2169, found 247.2162.

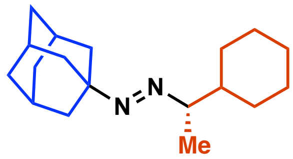

12

12 was prepared from **S12** (102 mg, 0.3 mmol) following **general procedure D**. Column chromatography (SiO_2, 5:95 EtOAc:hexanes) afforded **12** as a yellow oil (72 mg, 88%).

R_f = 0.62 (5:95 EtOAc:hexanes)
^{1}H NMR ($CDCl_3$, 400 MHz) δ: 3.10 (p, J = 6.8 Hz, 1 H), 2.15–2.13 (m, 3 H), 1.81–1.56 (m, 18 H), 1.32–1.14 (m, 3 H), 1.12 (d, J = 6.6 Hz, 3 H), 1.01–0.88 (m, 2 H) ppm.
^{13}C NMR (126 MHz, $CDCl_3$) δ: 77.8, 67.2, 42.1, 40.7, 36.8, 29.8, 29.6, 29.5, 26.7, 26.5, 26.3, 16.4 ppm.
HRMS(+ESI) calc'd for $C_{18}H_{30}N_2$ $[M+H]^+$ 275.2482, found 275.2477.
Optical rotation: $[\alpha]_D^{23}$(c = 20 mg/mL, CCl_4) = –29º.

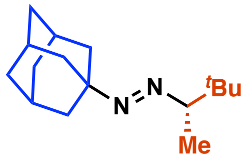

13

13 was prepared from **S13** (94.4 mg, 0.3 mmol) following **general procedure D**. Column chromatography (SiO_2, hexanes) afforded **13** as a colorless oil (53 mg, 71%).

Rf = 0.40 (hexanes)
^{1}H NMR (500 MHz, $CDCl_3$) δ: 3.02 (q, J = 6.8 Hz, 1 H), 2.16–2.14 (m, 3 H), 1.81–1.67 (m, 12 H), 1.05 (d, J = 6.8 Hz, 3 H), 0.93 (s, 9 H) ppm.
^{13}C NMR (126 MHz, $CDCl_3$) δ: 81.0, 67.4, 40.6, 36.8, 33.8, 29.5, 26.8, 14.1 ppm.
HRMS(+APCI) calc'd for $C_{16}H_{28}N_2$ $[M+H]^+$ 249.2325, found 249.2321.
Optical rotation: $[\alpha]_D^{23}$(c = 15 mg/mL, CCl_4) = –55º.

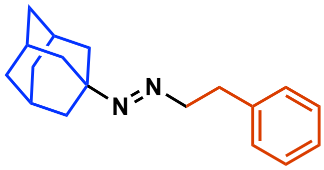

14

14 was prepared from **S14** (100 mg, 0.3 mmol) following **general procedure D**. Column chromatography (SiO_2, 2:98 EtOAc:hexanes) afforded **14** as a yellow solid (60 mg, 75%).

R_f = 0.45 (5:95 EtOAc:hexanes)

^{1}H NMR (500 MHz, $CDCl_3$) δ: 7.29–7.18 (m, 5 H, *overlaps with* $CDCl_3$), 4.05 (t, J = 7.6 Hz, 2 H), 3.07 (t, J = 7.6 Hz, 2 H), 2.16–2.14 (m, 3 H), 1.76–1.66 (m, 12 H) ppm.
^{13}C NMR (126 MHz, $CDCl_3$) δ: 139.7, 129.1, 128.5, 126.2, 70.4, 67.8, 40.5, 36.7, 34.3, 29.4 ppm.
HRMS(+ESI) calc'd for $C_{18}H_{24}N_2$ $[M+H]^+$ 269.2012, found 269.2010.

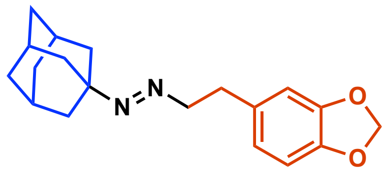

15

15 was prepared from **S15** (113.5 mg, 0.3 mmol) following **general procedure D**. Column chromatography (SiO_2,hexanes) afforded **15** as a yellow oil (44 mg, 47%).

R_f = 0.80 (2:98 EtOAc:hexanes)
^{1}H NMR (500 MHz, $CDCl_3$) δ: 6.73–6.71 (m, 2 H), 6.65 (dd, J = 7.8, 1.8 Hz, 1 H), 5.91 (s, 2 H), 3.99 (t, J = 7.5 Hz, 2 H), 2.99 (t, J = 7.5 Hz, 2 H), 2.16–2.14 (m, 3 H), 1.77–1.66 (m, 12 H) ppm.
^{13}C NMR (126 MHz, $CDCl_3$) δ: 147.7, 146.0, 133.4, 121.9, 109.6, 108.3, 100.9, 70.6, 67.9, 40.5, 36.7, 34.1, 29.4 ppm.
HRMS(+ESI) calc'd for $C_{19}H_{24}N_2O_2$ $[M+H]^+$ 313.1911, found 313.1923.

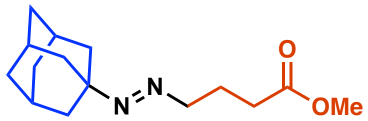

16

16 was prepared from **S16** (99 mg, 0.3 mmol) following **general procedure D**. Column chromatography (SiO_2, 5:95 EtOAc:hexanes) afforded **16** as a white solid (51 mg, 64%).

R_f = 0.22 (5:95 EtOAc:hexanes)
^{1}H NMR (500 MHz, $CDCl_3$) δ: 3.77 (t, J = 6.9 Hz, 2 H), 3.68 (s, 3 H), 2.39 (t, J = 7.6 Hz, 2 H), 2.16–2.08 (m, 5 H), 1.76 – 1.66 (m, 12 H) ppm.
^{13}C NMR (126 MHz, $CDCl_3$) δ: 173.7, 68.1, 67.9, 51.7, 40.5, 36.7, 31.8, 29.4, 23.3 ppm.
HRMS(+APCI) calc'd for $C_{15}H_{24}N_2O_2$ $[M+H]^+$ 265.1911, found 265.1903.

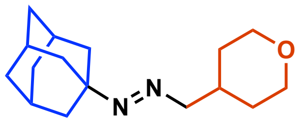

17

17 was prepared from **S17** (98.5 mg, 0.3 mmol) following **general procedure D**. Column chromatography (SiO_2, 10:90 to 20:80 EtOAc:hexanes) afforded **17** as a white solid (59 mg, 75%).

R_f = 0.79 (40:60 EtOAc:hexanes)
^{1}H NMR (500 MHz, $CDCl_3$) δ: 3.99–3.95 (m, 2 H), 3.67 (d, J = 6.7 Hz, 2 H), 3.45–3.39 (m, 2 H), 2.21–2.11 (m, 4 H), 1.77–1.66 (m, 12 H), 1.63–1.59 (m, 2 H), 1.48–1.40 (m, 2 H) ppm.
^{13}C NMR (126 MHz, $CDCl_3$) δ: 75.0, 68.0, 40.6, 36.7, 34.6, 31.4, 29.4 ppm.
HRMS(+APCI) calc'd for $C_{16}H_{26}N_2O$ $[M+H]^+$ 263.2110, found 263.2118.

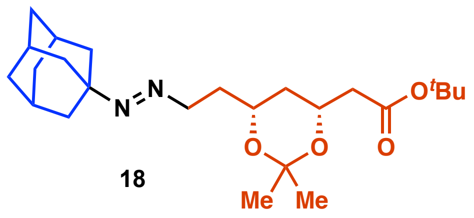

18

18 was prepared from **S18** (146 mg, 0.3 mmol) following **general procedure D**. Column chromatography (SiO_2, 5:95 EtOAc:hexanes) afforded **18** as a light yellow solid (73 mg, 58%).

R_f = 0.20 (5:95 EtOAc:hexanes)
^{1}H NMR (500 MHz, $CDCl_3$) δ: 4.23 (dtd, J = 11.6, 6.6, 2.4 Hz, 1 H), 3.95–3.87 (m, 1 H), 3.82 (tt, J = 8.0, 3.6 Hz, 2 H), 2.42 (dd, J = 15.1, 7.0 Hz, 1 H), 2.29 (dd, J = 15.1, 6.1 Hz, 1 H), 2.17–2.12 (m, 3 H), 1.99–1.85 (m, 2 H), 1.78–1.63 (m, 12 H), 1.59 (dt, J = 12.7, 2.5 Hz, 1 H), 1.44 (s, 9 H), 1.41 (s, 3 H), 1.36 (s, 3 H), 1.22 (dd, J = 11.9, 11.8 Hz, 1 H) ppm.
^{13}C NMR (126 MHz, $CDCl_3$) δ: 170.5, 98.9, 80.7, 67.7, 66.9, 66.4, 65.0, 42.9, 40.5, 36.7, 34.4, 30.2, 29.4, 28.3, 19.8 ppm.
HRMS(+ESI) calc'd for $C_{24}H_{40}N_2O_4$ $[M+H]^+$ 421.3061, found 421.3052.
Optical rotation: $[\alpha]_D^{23}$(c = 15 mg/mL, CCl_4) = +5°.

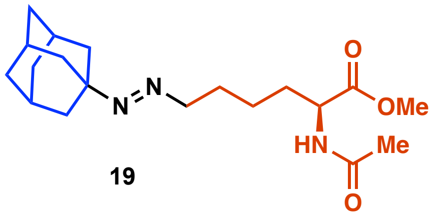

19

19 was prepared from **S19** (112.3 mg, 0.3 mmol) following **general procedure D**. Column chromatography (SiO_2, 40:60 EtOAc:hexanes) afforded **19** as a yellow oil (85 mg, 81%).

R_f= 0.42 (5:95 MeOH:DCM)
^{1}H NMR (500 MHz, $CDCl_3$) δ: 6.00 (d, J = 8.1 Hz, 1 H), 4.61 (td, J = 7.6, 5.4 Hz, 1 H), 3.78–3.68 (m, 5 H), 2.14 (br, 3 H), 2.00 (s, 3 H), 1.90–1.82 (m, 1 H), 1.81–1.62 (m, 15 H), 1.43–1.26 (m, 2 H) ppm.
^{13}C NMR (126 MHz, $CDCl_3$) δ: 173.2, 169.8, 68.7, 67.7, 52.5, 52.2, 40.5, 36.7, 32.4, 29.4, 27.4, 23.3, 22.9 ppm.
HRMS(+ESI) calc'd for $C_{19}H_{31}N_3O_3$ $[M+H]^+$ 350.2438, found 350.2426.
Optical rotation: $[\alpha]_D^{23}$(c = 20 mg/mL, CCl_4) = +7°.

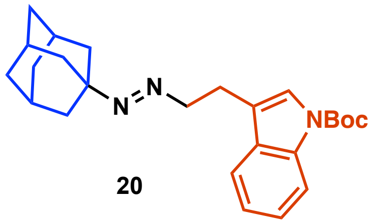

20

20 was prepared from **S20** (100.2 mg, 0.3 mmol) following **general procedure D**. Column chromatography (SiO_2, 2:98 EtOAc:hexanes) afforded **20** as a yellow oil (41.5 mg, 34%).

R_f= 0.60 (5:95 EtOAc:hexanes)
^{1}H NMR (500 MHz, $CDCl_3$) δ: 8.14 (d, J = 8.2 Hz, 1 H), 7.57 (d, J = 7.4 Hz, 1 H), 7.37 (br, 1 H), 7.31 (t, J = 7.4 Hz, 1 H), 7.24 (t, J = 7.3 Hz, 1 H), 4.11 (t, J = 7.3 Hz, 2 H), 3.17 (t, J = 7.3 Hz, 2 H), 2.16–2.15 (m, 3 H), 1.73 (d, J = 41.9 Hz, 12 H), 1.66 (s, 9 H) ppm.
^{13}C NMR (126 MHz, $CDCl_3$) δ: 149.9, 135.6, 130.8, 124.4, 123.4, 122.5, 119.2, 118.5, 115.3, 83.4, 68.6, 67.9, 40.4, 36.7, 29.4, 28.4, 23.4 ppm.

HRMS(+ESI) calc'd for $C_{25}H_{33}N_3O_2$ $[M+H]^+$ 408.2646, found 408.2635.

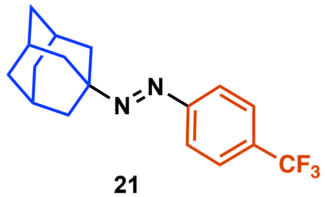

21

21 was prepared from **S21** (112.3 mg, 0.3 mmol) following **general procedure D**. Column chromatography (SiO_2, 1:99 EtOAc:hexanes) afforded **21** as a yellow solid (65 mg, 70%).

R_f = 0.83 (5:95 EtOAc:hexanes)
^{1}H NMR ($CDCl_3$, 400 MHz) δ: 7.71 (s, 4 H), 2.23 (br, 3 H), 1.93 (d, *J* = 2.9 Hz, 6 H), 1.85–1.70 (m, 6 H) ppm.
^{13}C NMR (126 MHz, $CDCl_3$) δ: 154.8, 131.6 (q, *J* = 32.3 Hz), 126.3 (q, *J* = 3.8 Hz), 124.2 (q, *J* = 272.2 Hz), 122.2, 69.6, 40.5, 36.7, 29.4 ppm.
^{19}F NMR ($CDCl_3$, 470 MHz) δ: 62.4 ppm.
HRMS(+APCI) calc'd for $C_{17}H_{19}F_3N_2$ $[M+H]^+$ 309.1573, found 309.1566.

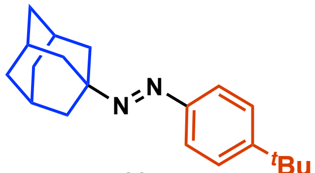

22

22 was prepared from **S22** (108.7 mg, 0.3 mmol) following **general procedure D**. Column chromatography (SiO_2, hexanes) afforded **22** as a yellow solid (46 mg, 52%).

R_f = 0.47 (5:95 EtOAc:hexanes)
^{1}H NMR (400 MHz, $CDCl_3$) δ 7.59 (d, *J* = 8.5 Hz, 2 H), 7.45 (d, *J* = 8.6 Hz, 2 H), 2.21–2.19 (m, 3 H), 1.92 (d, *J* = 2.9 Hz, 5H), 1.81–1.71 (m, 6H), 1.43 (s, 9 H) ppm.
^{13}C NMR (101 MHz, $CDCl_3$) δ 153.3, 150.7, 125.9, 121.6, 68.3, 40.6, 36.8, 35.0, 31.5, 29.5 ppm.
HRMS(+ESI) calc'd for $C_{20}H_{28}N_2$ $[M+H]^+$ 297.2325, found 297.2321.

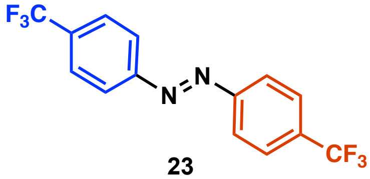

23

23 was prepared from **27** (3x38 mg, 0.1 mmol) following **general procedure E**. Column chromatography (SiO_2, 5:95 EtOAc:hexanes) afforded **23** as a red solid (39 mg, 41%).

The spectroscopic data for this compound were identical to those reported in the literature (*Angew. Chem. Int. Ed.* **2017**, *56*, 870–873).

^{1}H NMR ($CDCl_3$, 400 MHz) δ: 8.04 (d, *J* = 8.2 Hz, 4 H), 7.81 (d, *J* = 8.2 Hz, 4 H) ppm.

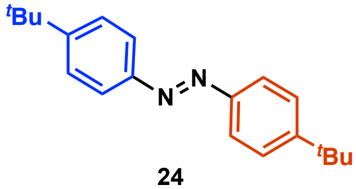

24

24 was prepared from **S23** (3x36 mg, 0.1 mmol) following **general procedure E**. Column chromatography (SiO_2, 5:95 EtOAc:hexanes) afforded **24** as an orange solid (13 mg, 15%).

The spectroscopic data for this compound were identical to those reported in the literature (*Green Chem.* **2019**, *21*, 4055–4061).

^{1}H NMR ($CDCl_3$, 400 MHz) δ: 7.86 (d, J = 8.4 Hz, 4 H), 7.54 (d, J = 8.4 Hz, 4 H), 1.39 (s, 18 H) ppm.

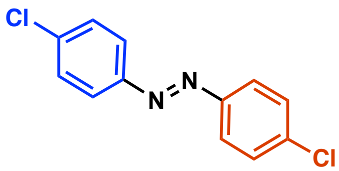

25

25 was prepared from **S24** (3x32 mg, 0.3 mmol) following **general procedure E**. Column chromatography (SiO_2, 5:95 EtOAc:hexanes) afforded **25** as a yellow solid (21 mg, 28%).

The spectroscopic data for this compound were identical to those reported in the literature (*Angew.Chem.Int.Ed.* **2017***, 56,* 870–873).

^{1}H NMR ($CDCl_3$, 400 MHz) δ: 7.87 (d, J = 8.4 Hz, 4 H), 7.49 (d, J = 8.4 Hz, 4 H) ppm.

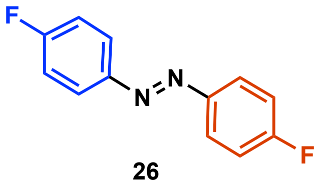

26

26 was prepared from **S25** (3x28 mg, 0.3 mmol) following **general procedure C**. Column chromatography (SiO_2, 5:95 EtOAc:hexanes) afforded **26** as a brown solid (21 mg, 32%).

The spectroscopic data for this compound were identical to those reported in the literature (*Angew. . Int. Ed* .**2017**, *56*, 870–873).

^{1}H NMR ($CDCl_3$, 400 MHz) δ: 7.89–7.94 (m, 4 H), 7.15–7.24 (m, 4 H) ppm.

<u>Optimization of the Synthesis of **23**</u>

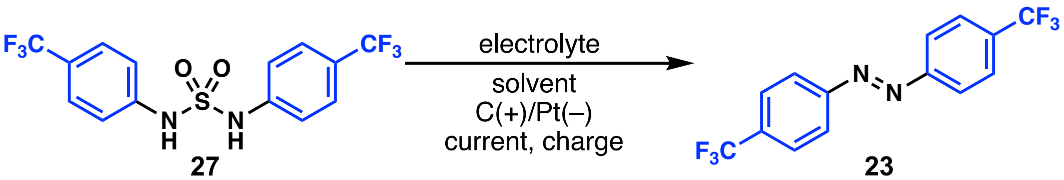

An oven dried ElectraSyn vial was equipped with a magnetic stir bar and vial was charged with sulfamide (1.0 equiv, 0.10 mmol) and corresponding solids.The ElectraSyn vial was then sealed with a cap equipped with anode and cathode. Solvent (5 mL, C = 0.02 M) was then added, and stirring was applied for 20 min (or until the sulfamide was fully dissolved). The reaction mixture was electrolyzed under 3 mA constant current and with 2–6 F/mol total charge. The solvent was then removed and yields were calculated by ^{1}H-NMR using 1,2,4,5-tetramethylbenzene as internal standard.

entry	electrolyte	solvent	current and charge	base	yield
**1**	NBu_4PF_6 (0.03 equiv)	HFIP (0.04 M)	0.5 mA and 2 F/mol	–	traces
**2**	NBu_4PF_6 (0.03 equiv)	MeOH (0.04 M)	0.5 mA and 2 F/mol	–	traces
**3**	LiCl (2.0 equiv)	MeOH (0.02 M)	3 mA and 2 F/mol	–	9%
**3**	LiCl (2.0 equiv)	MeOH (0.02 M)	3 mA and 6 F/mol	–	13%
**4**[a]	LiCl (2.0 equiv)	MeOH (0.02 M)	3 mA and 6 F/mol	–	23%
**5**[a]	LiCl (2.0 equiv)	MeOH (0.02 M)	3 mA and 6 F/mol	Cs_2CO_3	32%
**6**[a,b]	LiCl (2.0 equiv)	MeOH (0.02 M)	3 mA and 6 F/mol	Cs_2CO_3	40%
**7**[a,c]	LiCl (2.0 equiv)	MeOH (0.02 M)	3 mA and 6 F/mol	Cs_2CO_3	39%
**7**[a,d]	LiCl (2.0 equiv)	MeOH (0.02 M)	3 mA and 6 F/mol	Cs_2CO_3	0%
**8**[a]	KPF_6 (2.0 equiv)	MeOH (0.02 M)	3 mA and 6 F/mol	Cs_2CO_3	38%

Table S1. [a]RVC(+) instead of C(+). [b]Without degassing. [c]Under O_2 atmosphere. [d]No electricity.

Isolation of phenazine **30** during the oxidation of *N,N'*-diarylsulfamide **28**

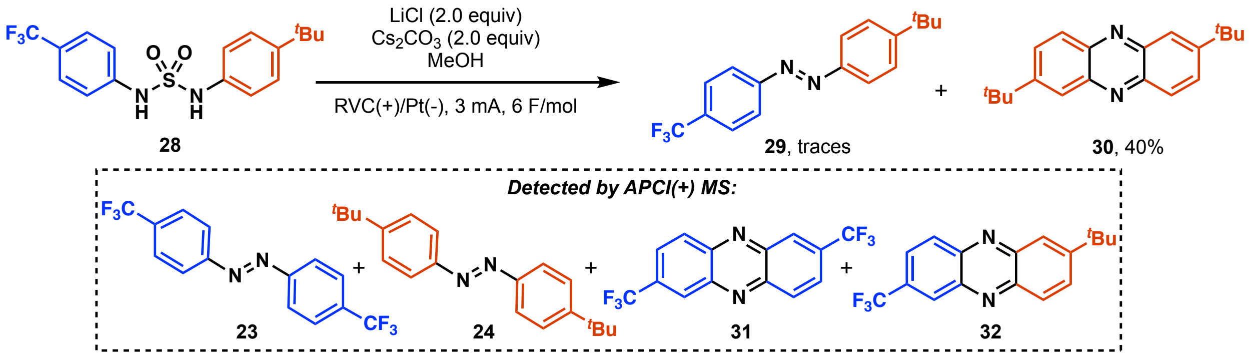

Figure S1. Isolation of phenazine **30** during the oxidation of *N,N'*-diarylsulfamide **28**

Three oven dried ElectraSyn vials equipped with a magnetic stir bar were charged with solids, sulfamide **28** (1.0 equiv, 0.1 mmol), Cs_2CO_3 (2.0 equiv, 0.20 mmol), and LiCl (2.0 equiv, 0.20 mmol). The ElectraSyn vial was then sealed with a cap equipped with anode (RVC) and cathode (Pt foil). MeOH (5 mL, C = 0.02 M) was then added, and stirring was applied for 20 min (or until the sulfamide was fully dissolved). The reaction mixture was electrolyzed under 3 mA constant current and with 6 F/mol total charge. After the reaction was stopped, the solvent was removed under vacuo and reaction by analyzed by APCI(+) MS. Phenazine **30** was observed as a major product and isolated in 40% yield. Diazene **29, 23, 24,** and phenazine **31, 32** were observed by APCI(+) MS.

Oxidation of unsymmetrical sulfamide **S21** (aryl-alkyl)

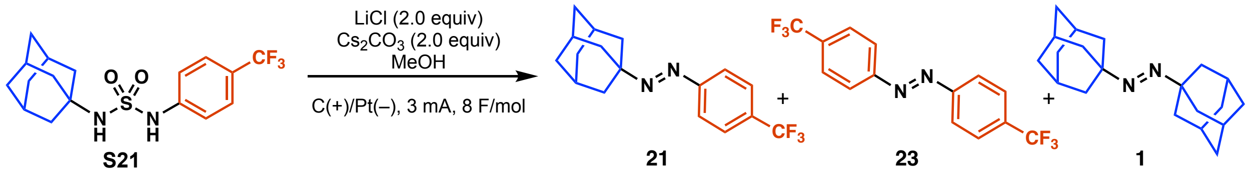

Figure S2. Oxidation of sulfamide **S21**.

An oven dried ElectraSyn vial equipped with a magnetic stir bar and was charged with solids, sulfamide **S21** (1.0 equiv, 0.1 mmol), Cs_2CO_3 (2.0 equiv, 0.20 mmol), and LiCl (2.0 equiv, 0.20 mmol). The ElectraSyn vial was then sealed with a cap equipped with selected anode (graphite) and cathode (Pt foil). MeOH (2.5 mL, C = 0.04 M), and stirring was applied for 20 min (or until the sulfamide was fully dissolved). The reaction mixture was electrolyzed under 3 mA constant current and with 8 F/mol total charge. After the reaction was stopped, the solvent was removed under vacuo and 1,2,4,6-tetramethylbenzene was added as an internal standard showing phenazine **21** as a major product in 71% (^{1}H NMR yield). Diazene **23** was also observed in 6% (^{1}H NMR yield) and diazene **1** was not detected.

Representative Synthesis of diazene **3** (1 mmol scale):

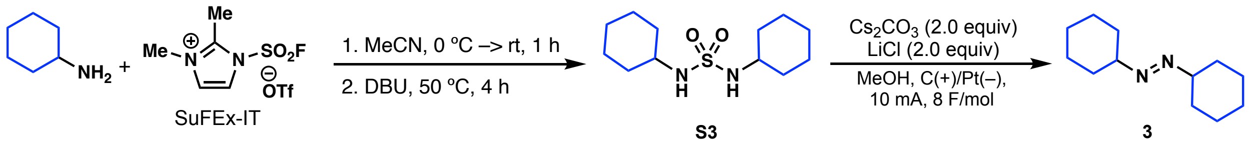

S3 was prepared from SuFEx-IT (1.06 g, 3.2 mmol) and cyclohexylamine (500 mg, 6.8 mmol) following **general procedure C**. Column chromatography (SiO_2, 20:80 EtOAc:hexanes) afforded **S3** as a white solid (620 mg, 73%).

An oven dried 25 mL two-neck round bottom flask equipped with a magnetic stir bar was charged with sulfamide **S4** (260.4 mg, 1.0 equiv, 1.0 mmol), Cs_2CO_3 (652 mg ,2.0 equiv, 2.0 mmol), and LiCl (76 mg, 2.0 equiv, 1.0 mmol). The flask was then sealed with a septum equipped with a graphite anode and a platinum foil cathode. MeOH (25 mL, C = 0.04 M). was then added, and the mixture was stirred for 20 min. The reaction mixture was electrolyzed under a constant current of 10 mA using DC Power Supply (Agilent, E3612A) for a total charge of 8 F/mol (~22 h). The progression of the reaction was monitored by TLC. Upon completion, the solvent was removed under vacuo and the reaction mixture was filtered through a celite plug using EtOAc (~20 mL). The solvent was removed, and the product was directly purified by column chromatography to afford the desired product (96 mg, 49%).

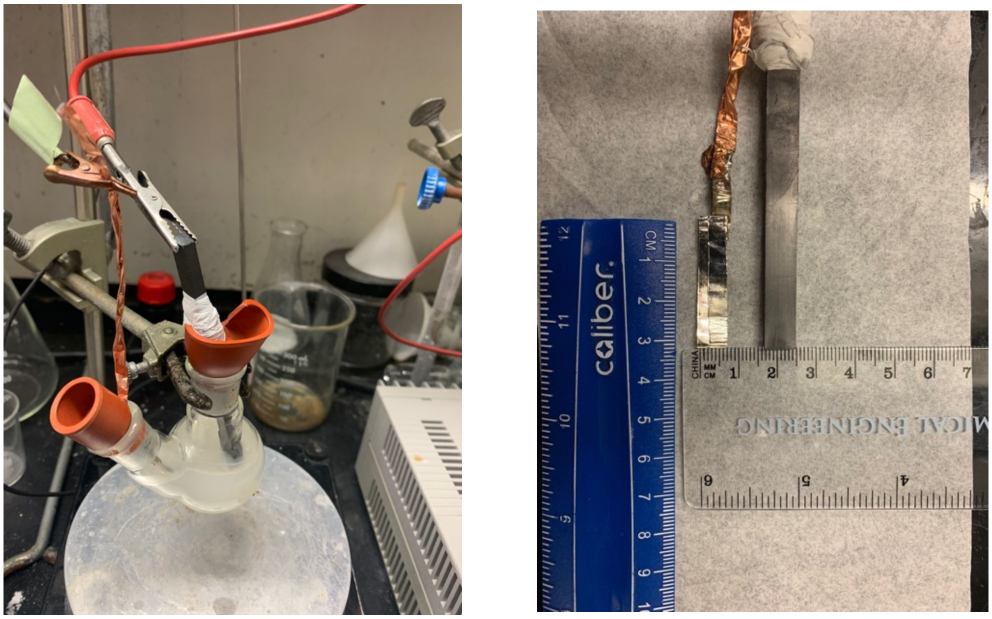

Figure S3. *Left:* Mixture of **S3,** Cs_2CO_3, LiCl, and MeOH under 10 mA constant current. *Right:* Graphite and platinum foil used in the scale-up electrolysis.

Characterization of isolated byproducts

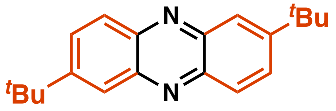

30

30 was isolated as a byproduct from the reaction with **29** (3x37.2 mg, 0.3 mmol) following **general procedure E**. Column chromatography (SiO_2, 1:99 EtOAc:hexanes) afforded **30** as a brown solid (17.5 mg, 40%).

The spectroscopic data for this compound were identical to those reported in the literature (*J. Org. Chem.* **2020**, 85, 23, 15154–15166).

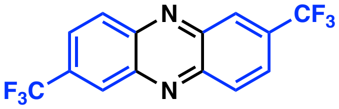

31

31 was isolated as a byproduct from the reaction with **27** (3x38 mg, 0.3 mmol) following **general procedure E**. Column chromatography (SiO_2, 5:95 EtOAc:hexanes) afforded **31** as a brown solid (8.5 mg, >1%).

The spectroscopic data for this compound were identical to those reported in the literature (*J. Org. Chem.* **2020**, 85, 23, 15154–15166).

^{1}H NMR ($CDCl_3$, 400 MHz) δ: 8.63 (s, 2 H), 8.43 (d, J = 8.9 Hz, 2 H), 8.04 (dd, J = 1.4, 8.9 Hz, 2 H) ppm.

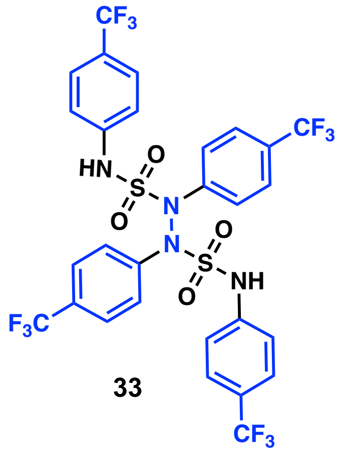

33

33 was isolated as a byproduct from the reaction with **27** (3x38 mg, 0.3 mmol) following **general procedure D** *(KI was used instead of LiCl)*. Column chromatography (SiO_2, 30:70 EtOAc:hexanes) afforded **33** as a yellow solid (39 mg, 33%).

R_f = 0.40 (50:50 EtOAc:hexanes)
^{1}H NMR (400 MHz, $CDCl_3$) δ: 7.52–7.43 (m, 12 H), 7.16 (d, J = 8.3 Hz, 4 H) ppm.
^{13}C NMR (126 MHz, $CDCl_3$) δ: 142.0, 139.3, 129.6 (q, J = 33.4 Hz), 128.54 (q, J = 33.1 Hz), 126.55 (q, J = 3.2 Hz), 126.47 (q, J = 3.2 Hz), 123.6 (q, J = 272.0 Hz), 123.3 (q, J = 273.0 Hz), 122.8, 122.6 ppm.

HRMS(–ESI) calc'd for $C_{28}H_{18}F_{12}N_4O_4S_2$ $[M–H]^-$ 765.0505, found 765.0515.

Postulated mechanism for the formation of phenazines:

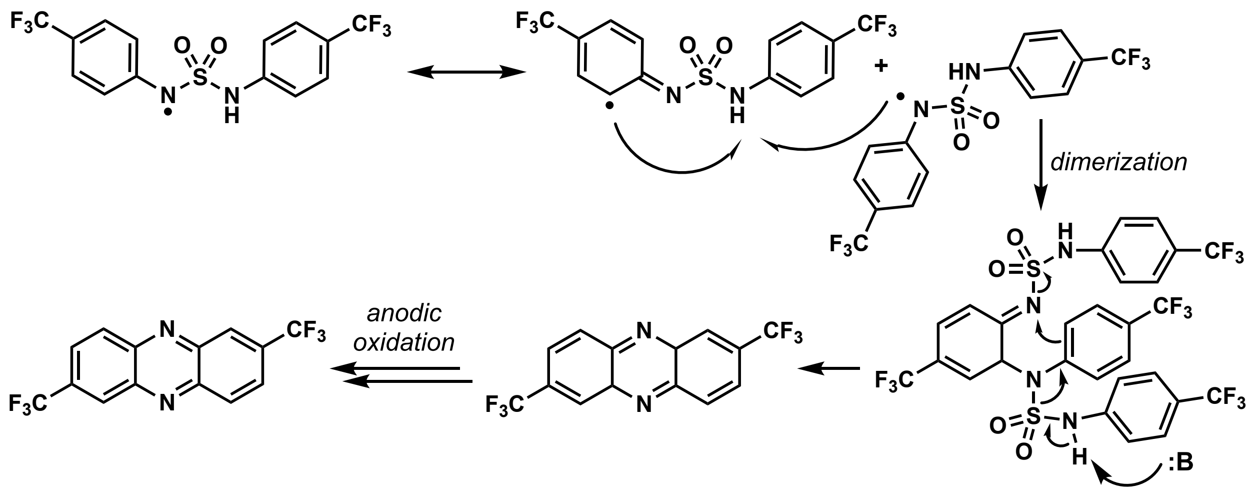

Figure S4. Postulated mechanism for the formation of phenazines during the electro-oxidation of *N,N'*-diarylsulfamides.

CV of sulfamide **1** in MeOH with and without carbonate base:

The solutions used for the CV were 10 mM of **1**, 10 mM of **1** and 20 mM of Cs_2CO_3, 20 mM of Cs_2CO_3 with the supporting electrolyte ^{n}BuNPF$_6$ (C = 100 mM in MeOH (10 mL), using the voltage scan rate of 100 mV/s. Solutions were thoroughly bubbled with dry argon for 15 minutes to remove oxygen before any experiment and kept under positive pressure of argon.

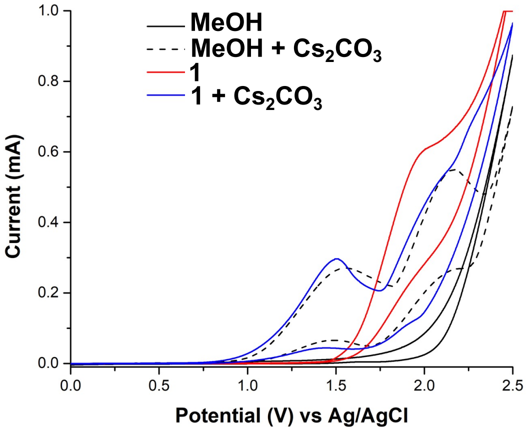

Figure S5. CV of **1** (C = 10 mM) in MeOH in the presence or absence of Cs_2CO_3 (2 equiv) using ^{n}BuNPF$_6$ (C = 100 mM) as supporting electrolyte, a glassy carbon electrode, a platinum wire auxiliary electrode, and a Ag/AgCl reference. Scan rate of 100 mV/s and direction of the oxidative scan from 0 to +2.5 V (IUPAC convention).

CV of sulfamide **27** in MeOH with and without carbonate base:

The solutions used for the CV were 10 mM of **27**, 10 mM of **27** and 20 mM of Cs_2CO_3, 20 mM of Cs_2CO_3 with the supporting electrolyte TBAPF$_6$ (c = 0.1 M) in MeOH (10 mL), using the voltage scan rate of 100 mV/s. Solutions were thoroughly bubbled with dry argon for 15 minutes to remove oxygen before any experiment and kept under positive pressure of argon.

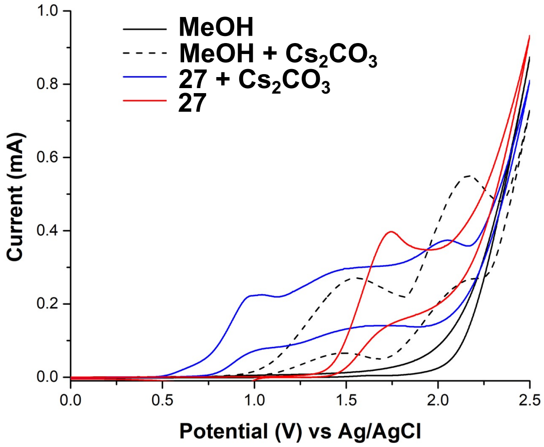

Figure S6. CV of **27** (C = 10 mM) in MeOH in the presence or absence of Cs_2CO_3 (2 equiv) using ^{n}BuNPF$_6$ (C = 100 mM) as supporting electrolyte, a glassy carbon electrode, a platinum wire auxiliary electrode, and a Ag/AgCl reference. Scan rate of 100 mV/s and direction of the oxidative scan from 0 to +2.5 V (IUPAC convention).

CV of LiCl in MeOH:

The solutions used for the CV were 20 mM of LiCl with the supporting electrolyte ^{n}BuNPF$_6$ (C = 100 mM in MeOH (10 mL), using the voltage scan rate of 100 mV/s. Solutions were thoroughly bubbled with dry argon for 15 minutes to remove oxygen before any experiment and kept under positive pressure of argon.

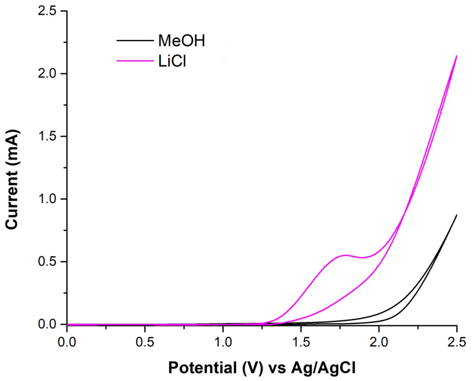

Figure S7. CV of LiCl (C = 20 mM) in MeOH using ^{n}BuNPF$_6$ (C = 100 mM) as supporting electrolyte, a glassy carbon electrode, a platinum wire auxiliary electrode, and a Ag/AgCl reference. Scan rate of 100 mV/s and direction of the oxidative scan from 0 to +2.5 V (IUPAC convention).

CV of sulfamide **1** LiCl, and base in MeOH:

The solutions used for the CV were 10 mM of **1**; 10 mM of **1** and LiCl 20 mM, and 20 mM of Cs_2CO_3; 20 mM of LiCl and 20 mM of Cs_2CO_3 with the supporting electrolyte TBAPF$_6$ (c = 0.1 M) in MeOH (10 mL), using the voltage scan rate of 100 mV/s. Solutions were thoroughly bubbled with dry argon for 15 minutes to remove oxygen before any experiment and kept under positive pressure of argon.

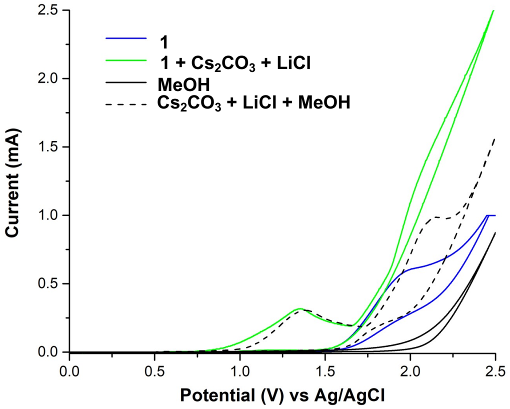

Figure S8. CV of **1** (C = 10 mM) in MeOH in the presence or absence of Cs_2CO_3 (2 equiv) using LiCl (C = 20 mM) and ^{n}BuNPF$_6$ (C = 100 mM) as supporting electrolyte, a glassy carbon electrode, a platinum wire auxiliary electrode, and a Ag/AgCl reference. Scan rate of 100 mV/s and direction of the oxidative scan from 0 to +2.5 V (IUPAC convention).

CV of sulfamide **27** LiCl, and base in MeOH:

The solutions used for the CV were 10 mM of **27**; 10 mM of **27** and LiCl 20 mM, and 20 mM of Cs_2CO_3; 20 mM of LiCl and 20 mM of Cs_2CO_3 with the supporting electrolyte $TBAPF_6$ (c = 0.1 M) in MeOH (10 mL), using the voltage scan rate of 100 mV/s. Solutions were thoroughly bubbled with dry argon for 15 minutes to remove oxygen before any experiment and kept under positive pressure of argon.

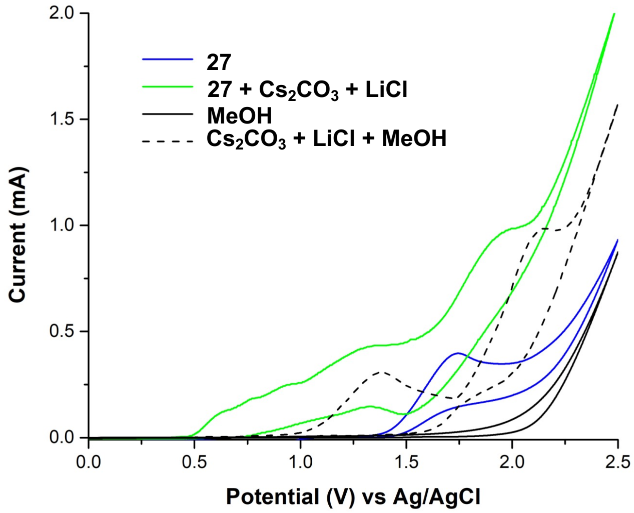

Figure S9. CV of **27** (C = 10 mM) in MeOH in the presence or absence of Cs_2CO_3 (2 equiv) using LiCl (C = 20 mM) and $^nBuNPF_6$ (C = 100 mM) as supporting electrolyte, a glassy carbon electrode, a platinum wire auxiliary electrode, and a Ag/AgCl reference. Scan rate of 100 mV/s and direction of the oxidative scan from 0 to +2.5 V (IUPAC convention).

Coulometric experiment with **27**:

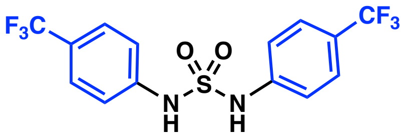

27

An oven dried ElectraSyn vials equipped with a magnetic stir bar was charged with sulfamide **27** (73.0 mg, 0.2 mmol), $TBAPF_6$ (c = 0.1 M), and MeCN (7 mL c = 0.03 M). The ElectraSyn vial was then sealed with a cap equipped with anode (graphite), cathode (Pt foil), and reference electrode Ag/Ag^+ (1 mM $AgNO_3$ + $^nBuNPF_6$ (C = 100 mM)) system in acetonitrile as the reference electrode. The reaction mixture was electrolyzed at a constant potential of 1.95 V using a CH Instruments Electrochemical Analyzer. The initial current was noted around 50 mA and the reaction was stopped after the current has stabilized around 5 mA (~12 F/mol) without further evolution. This experiment suggests that competitive pathways are taking place during the electrolysis.

X-ray crystallographic data

X-ray crystallographic data for **30**:

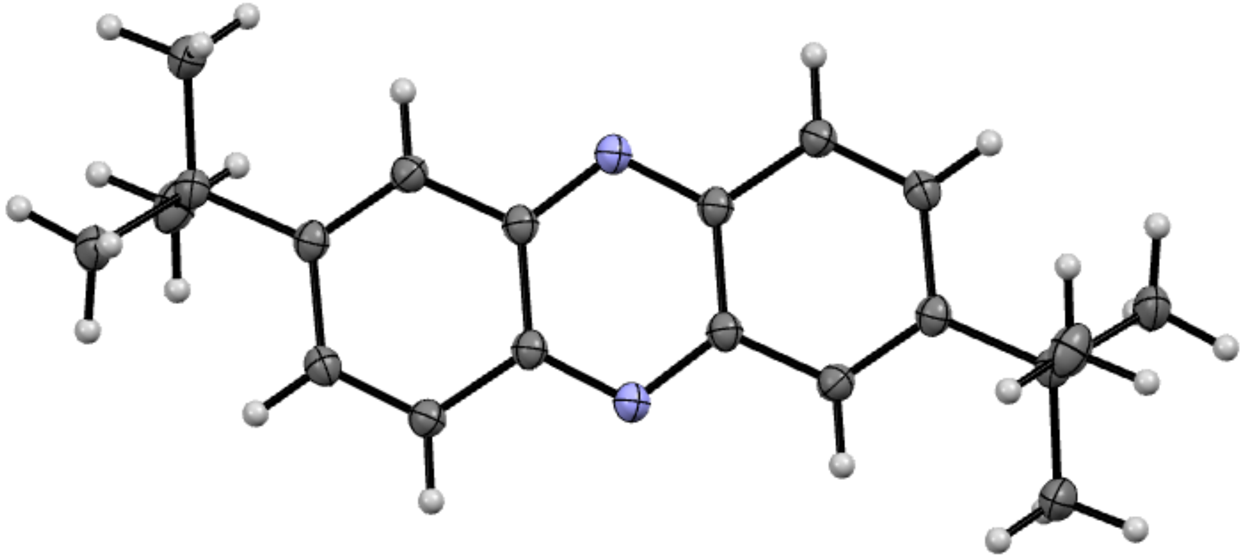

Figure S10. ORTEP drawing of **30** showing thermal ellipsoids at the 50% probability level.

Table S2 Crystal data and structure refinement for 30.

Identification code	30
Empirical formula	$C_{20}H_{24}N_2$
Formula weight	292.41
Temperature/K	110.00(10)
Crystal system	monoclinic
Space group	$P2_1/c$
a/Å	12.2978(2)
b/Å	6.13330(10)
c/Å	10.84910(10)
α/°	90
β/°	95.4420(10)
γ/°	90
Volume/Å^3	814.62(2)
Z	2
ρ_{calc}g/cm^3	1.192
μ/mm^{-1}	0.530
F(000)	316.0
Crystal size/mm^3	0.1 × 0.1 × 0.01
Radiation	Cu Kα (λ = 1.54184)
2Θ range for data collection/°	14.47 to 156.27
Index ranges	$-15 \le h \le 15, -7 \le k \le 7, -13 \le l \le 9$

Reflections collected	12018
Independent reflections	1714 [R_{int} = 0.0470, R_{sigma} = 0.0199]
Data/restraints/parameters	1714/0/103
Goodness-of-fit on F^2	1.080
Final R indexes [I>=2σ (I)]	R_1 = 0.0448, wR_2 = 0.1237
Final R indexes [all data]	R_1 = 0.0472, wR_2 = 0.1272
Largest diff. peak/hole / e Å^{-3}	0.30/-0.23

Table S3 Fractional Atomic Coordinates (×10^4) and Equivalent Isotropic Displacement Parameters (Å^2×10^3) for 30. U_{eq} is defined as 1/3 of the trace of the orthogonalised U_{IJ} tensor.

Atom	*x*	*y*	*z*	U(eq)
N1	5579.6(7)	7015.4(14)	4972.2(8)	20.3(2)
C1	5864.5(8)	5464.3(17)	5817.0(9)	19.4(3)
C2	4717.8(8)	6561.2(17)	4156.2(9)	19.3(3)
C3	4357.0(9)	8126.0(17)	3234.4(9)	20.9(3)
C4	3484.8(8)	7684.4(18)	2398.9(9)	21.1(3)
C5	2897.5(8)	5655.1(17)	2401.9(9)	19.9(3)
C6	3230.5(8)	4140.4(17)	3283.9(9)	20.8(3)
C7	1897.7(9)	5304.6(18)	1465.3(9)	21.4(3)
C8	2124.4(10)	6124(2)	174.9(10)	29.6(3)
C9	935.6(9)	6573(2)	1922.1(11)	31.8(3)
C10	1590.5(10)	2888.4(19)	1342.6(11)	29.1(3)

Table S4 Anisotropic Displacement Parameters (Å^2×10^3) for 30. The Anisotropic displacement factor exponent takes the form: $-2\pi^2[h^2a^{*2}U_{11}+2hka^*b^*U_{12}+\ldots]$.

Atom	U_{11}	U_{22}	U_{33}	U_{23}	U_{13}	U_{12}
N1	21.5(5)	21.0(5)	18.4(4)	-0.2(3)	1.2(3)	-0.5(3)
C1	20.3(5)	20.6(5)	17.3(5)	-0.4(4)	2.1(4)	0.4(4)
C2	20.2(5)	20.7(5)	17.0(5)	-1.2(4)	2.1(4)	0.5(4)
C3	23.4(5)	19.7(5)	19.6(5)	0.2(4)	2.2(4)	-1.2(4)
C4	23.2(5)	21.8(5)	18.3(5)	1.9(4)	1.4(4)	1.7(4)
C5	19.4(5)	22.9(5)	17.6(5)	-1.3(4)	1.9(4)	0.8(4)
C6	21.2(5)	21.3(5)	19.8(5)	-0.9(4)	1.4(4)	-2.2(4)
C7	21.3(5)	23.4(5)	18.8(5)	0.6(4)	-1.1(4)	-0.5(4)
C8	32.8(6)	35.4(6)	19.4(5)	2.9(4)	-3.4(4)	-7.3(5)
C9	22.6(6)	39.5(7)	32.4(6)	-6.3(5)	-1.9(4)	3.6(5)

Table S4 Anisotropic Displacement Parameters ($Å^2×10^3$) for 30. The Anisotropic displacement factor exponent takes the form: $-2\pi^2[h^2a^{*2}U_{11}+2hka^*b^*U_{12}+…]$.

Atom	U_{11}	U_{22}	U_{33}	U_{23}	U_{13}	U_{12}
C10	30.1(6)	27.0(6)	28.0(6)	1.9(4)	-8.5(4)	-3.9(4)

Table S5 Bond Lengths for 30.

Atom	Atom	Length/Å	Atom	Atom	Length/Å
N1	C1	1.3443(14)	C4	C5	1.4391(15)
N1	C2	1.3440(13)	C5	C6	1.3682(15)
C1	$C2^1$	1.4356(15)	C5	C7	1.5342(13)
C1	$C6^1$	1.4293(14)	C7	C8	1.5376(14)
C2	C3	1.4262(14)	C7	C9	1.5368(15)
C3	C4	1.3643(14)	C7	C10	1.5319(15)

^{1}1-X,1-Y,1-Z

Table S6 Bond Angles for 30.

Atom	Atom	Atom	Angle/°	Atom	Atom	Atom	Angle/°
C2	N1	C1	116.15(9)	C6	C5	C4	118.16(9)
N1	C1	$C2^1$	121.86(9)	C6	C5	C7	122.41(10)
N1	C1	$C6^1$	118.75(10)	C5	C6	$C1^1$	121.61(10)
$C6^1$	C1	$C2^1$	119.38(9)	C5	C7	C8	110.74(9)
N1	C2	$C1^1$	121.99(10)	C5	C7	C9	107.98(8)
N1	C2	C3	119.72(10)	C9	C7	C8	109.86(9)
C3	C2	$C1^1$	118.28(9)	C10	C7	C5	111.63(9)
C4	C3	C2	120.33(10)	C10	C7	C8	107.55(9)
C3	C4	C5	122.23(9)	C10	C7	C9	109.07(9)
C4	C5	C7	119.38(9)				

^{1}1-X,1-Y,1-Z

Table S7 Torsion Angles for 30.

A	B	C	D	Angle/°	A	B	C	D	Angle/°
N1	C2	C3	C4	179.87(9)	C4	C5	C6	$C1^1$	-0.08(15)
C1	N1	C2	$C1^1$	-0.09(16)	C4	C5	C7	C8	43.34(13)
C1	N1	C2	C3	179.56(9)	C4	C5	C7	C9	-76.98(11)
$C1^1$	C2	C3	C4	-0.47(15)	C4	C5	C7	C10	163.13(9)

Table S7 Torsion Angles for 30.

A	B	C	D	Angle/°	A	B	C	D	Angle/°
C2	N1	C1	C2^1	0.09(16)	C6	C5	C7	C8	-139.55(10)
C2	N1	C1	C6^1	-179.51(9)	C6	C5	C7	C9	100.13(12)
C2	C3	C4	C5	-0.19(16)	C6	C5	C7	C10	-19.75(14)
C3	C4	C5	C6	0.47(15)	C7	C5	C6	C1^1	-177.22(9)
C3	C4	C5	C7	177.71(9)					

^{1}1-X,1-Y,1-Z

Table S8 Hydrogen Atom Coordinates (Å×10^4) and Isotropic Displacement Parameters (Å^2×10^3) for 30.

Atom	*x*	*y*	*z*	U(eq)
H3	4725.94	9482.78	3202.06	25
H4	3257.31	8752.62	1794.62	25
H6	2850.88	2793.09	3299.1	25
H8A	2224.08	7709.06	198.65	44
H8B	1505.43	5755.84	-425.37	44
H8C	2788.06	5428.8	-71	44
H9A	780.53	6012.9	2733.11	48
H9B	288.75	6392.21	1330.45	48
H9C	1124.2	8123.34	1994.16	48
H10A	2209.54	2062.96	1077.04	44
H10B	959.03	2721.22	727.7	44
H10C	1406.59	2335.52	2144.48	44

Experimental

Single crystals of $C_{20}H_{24}N_2$ **30** were grown using slow evaporation of chloroform. A suitable crystal was selected and analyzed on a **Bruker Photon 2 kappa microsource** diffractometer. The crystal was kept at 110.00 K during data collection. Using Olex2,[5] the structure was solved with the SHELXT[6] structure solution program using Intrinsic Phasing and refined with the SHELXL[7] refinement package using Least Squares minimisation.

Crystal structure determination of 30

Crystal Data for $C_{20}H_{24}N_2$ (M =292.41 g/mol): monoclinic, space group $P2_1/c$ (no. 14), a = 12.2978(2) Å, b = 6.13330(10) Å, c = 10.84910(10) Å, β = 95.4420(10)°, V = 814.62(2) Å^3, Z = 2, T = 110.00(10) K, μ(Cu Kα) = 0.530 mm^{-1}, $Dcalc$ = 1.192 g/cm^3, 12018 reflections measured (14.47° ≤ 2Θ ≤ 156.27°), 1714 unique (R_{int} = 0.0470, R_{sigma} = 0.0199) which were used in all calculations. The final R_1 was 0.0448 (I > 2σ(I)) and wR_2 was 0.1272 (all data).

Refinement model description

Number of restraints - 0, number of constraints - unknown.

Details:

1. Fixed Uiso
 At 1.2 times of:
  All C(H) groups
 At 1.5 times of:
  All C(H,H,H) groups

2.a Aromatic/amide H refined with riding coordinates:
 C3(H3), C4(H4), C6(H6)

2.b Idealised Me refined as rotating group:
 C8(H8A,H8B,H8C), C9(H9A,H9B,H9C), C10(H10A,H10B,H10C)

<u>X-ray crystallographic data for **31**</u>

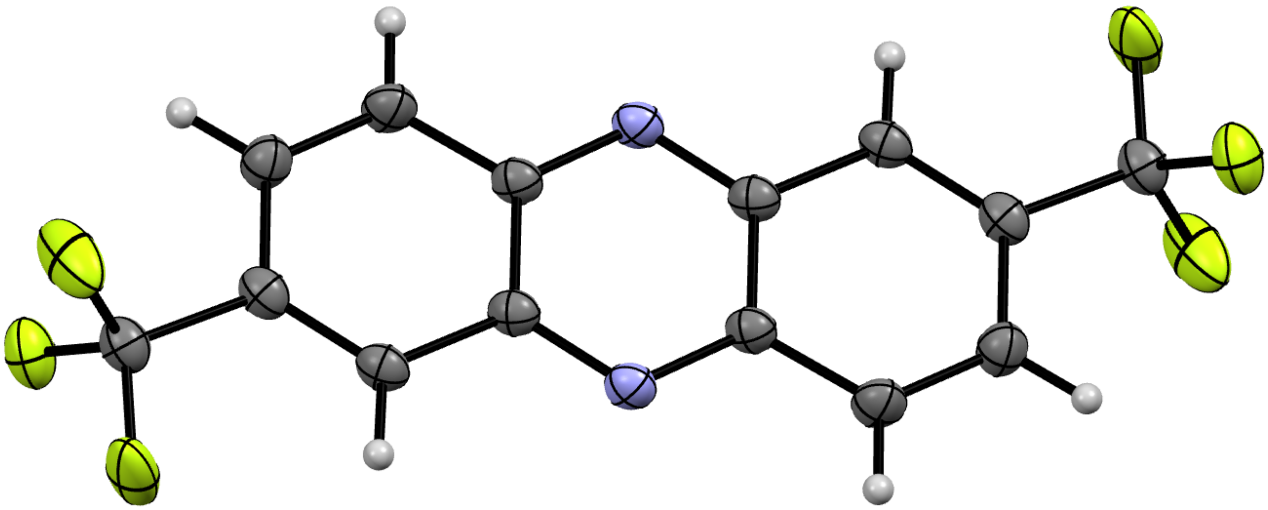

Figure S11. ORTEP drawing of **31** showing thermal ellipsoids at the 50% probability level.

Table S9 Crystal data and structure refinement for 31.

Identification code	**31**
Empirical formula	$C_{14}H_6F_6N_2$
Formula weight	316.21
Temperature/K	110.00(10)
Crystal system	triclinic
Space group	P-1
a/Å	7.19230(10)
b/Å	11.2284(3)
c/Å	12.9596(3)
α/°	66.282(2)

β/°	79.6040(10)
γ/°	88.7040(10)
Volume/Å^3	941.01(4)
Z	3
ρ_{calc}g/cm^3	1.674
μ/mm^{-1}	1.465
F(000)	474.0
Crystal size/mm^3	0.1 × 0.1 × 0.01
Radiation	Cu Kα (λ = 1.54184)
2Θ range for data collection/°	7.586 to 156.388
Index ranges	-7 ≤ h ≤ 8, -14 ≤ k ≤ 14, -16 ≤ l ≤ 16
Reflections collected	28316
Independent reflections	3936 [R_{int} = 0.0254, R_{sigma} = 0.0103]
Data/restraints/parameters	3936/0/298
Goodness-of-fit on F^2	1.066
Final R indexes [I>=2σ (I)]	R_1 = 0.0391, wR_2 = 0.1008
Final R indexes [all data]	R_1 = 0.0400, wR_2 = 0.1015
Largest diff. peak/hole / e Å^{-3}	0.69/-0.49

Table S10 Fractional Atomic Coordinates (×10^4) and Equivalent Isotropic Displacement Parameters (Å^2×10^3) for 31. U_{eq} is defined as 1/3 of the trace of the orthogonalised U_{IJ} tensor.

Atom	*x*	*y*	*z*	U(eq)
F1A	17741.3(13)	7234.8(10)	3878.1(8)	35.4(2)
F2A	16213.8(15)	8867.5(9)	2995.4(11)	49.6(3)
F3A	16811.0(14)	7390.4(12)	2350.5(8)	43.6(3)
N1A	9369.7(17)	5071.0(12)	6079.5(10)	23.5(2)
C1A	8343(2)	4430.5(13)	5679.6(11)	22.0(3)
C2A	11028(2)	5644.5(13)	5407.8(11)	22.3(3)
C3A	12193(2)	6335.5(15)	5789.3(12)	27.4(3)
C4A	13862(2)	6938.9(15)	5112.8(12)	27.0(3)
C5A	14472(2)	6868.8(13)	4026.3(12)	23.4(3)
C6A	13429(2)	6202.1(13)	3637.0(11)	23.1(3)
C7A	16310(2)	7584.3(14)	3309.6(12)	25.9(3)
F1B	11941.2(18)	1715.1(11)	4440.6(8)	49.4(3)
F2B	10165.5(16)	867.6(13)	3752.9(9)	56.4(3)
F3B	12872(2)	143.6(12)	3993.0(10)	67.7(4)
N1B	14963.4(15)	5424.6(11)	906.5(10)	18.8(2)
C1B	15699.9(17)	6190.7(12)	-191.0(11)	17.8(3)

Table S10 Fractional Atomic Coordinates (×10⁴) and Equivalent Isotropic Displacement Parameters (Å²×10³) for 31. U_{eq} is defined as 1/3 of the trace of the orthogonalised U_{IJ} tensor.

Atom	*x*	*y*	*z*	U(eq)
C2B	14268.7(17)	4234.5(12)	1102.0(11)	18.2(3)
C3B	13475.4(19)	3375.2(13)	2250.1(11)	20.7(3)
C4B	12791.8(18)	2157.9(13)	2466.2(12)	21.2(3)
C5B	12814.8(19)	1729.6(13)	1564.4(12)	21.5(3)
C6B	13533.2(18)	2534.9(13)	457.5(12)	20.3(3)
C7B	11958(2)	1222.2(14)	3666.8(12)	26.2(3)
F1C	11938.9(14)	10059.3(8)	81.6(8)	34.8(2)
F2C	10734.1(14)	8659.8(10)	1768.4(9)	38.3(2)
F3C	13669.5(12)	8659.0(9)	1085.0(8)	32.2(2)
N1C	9459.0(15)	4615.5(10)	1209.5(9)	17.9(2)
C1C	7963.0(18)	1721.6(13)	908.3(12)	20.2(3)
C2C	8276.1(18)	2550.7(13)	1397.9(11)	19.7(3)
C3C	9160.1(17)	3818.4(12)	704.9(11)	16.9(2)
C4C	10294.9(17)	5793.9(12)	512.7(11)	17.0(2)
C5C	10653.2(18)	6683.5(12)	1000.8(11)	19.0(3)
C6C	11495.2(18)	7884.0(13)	302.1(12)	19.4(3)
C7C	11945.7(19)	8808.7(13)	812.4(12)	22.2(3)

Table S11 Anisotropic Displacement Parameters (Å²×10³) for 31. The Anisotropic displacement factor exponent takes the form: $-2\pi^2[h^2a^{*2}U_{11}+2hka^*b^*U_{12}+\ldots]$.

Atom	U_{11}	U_{22}	U_{33}	U_{23}	U_{13}	U_{12}
F1A	25.3(5)	43.1(5)	32.0(5)	-8.1(4)	-7.7(4)	-2.8(4)
F2A	38.8(6)	22.9(5)	60.5(7)	1.7(5)	12.0(5)	-2.9(4)
F3A	28.5(5)	73.8(8)	27.6(5)	-23.8(5)	6.6(4)	-14.5(5)
N1A	26.5(6)	25.0(6)	17.0(5)	-8.1(5)	0.4(4)	-1.7(5)
C1A	25.3(7)	20.8(6)	16.8(6)	-5.7(5)	-1.4(5)	1.0(5)
C2A	25.5(7)	21.8(6)	16.9(6)	-6.4(5)	-0.7(5)	-0.1(5)
C3A	32.0(8)	30.9(7)	19.1(6)	-11.8(6)	0.1(5)	-3.7(6)
C4A	29.1(7)	28.5(7)	23.0(7)	-10.1(6)	-3.6(6)	-4.0(6)
C5A	23.4(7)	22.0(6)	19.1(6)	-3.6(5)	-1.7(5)	1.0(5)
C6A	25.2(7)	23.8(7)	16.5(6)	-5.6(5)	-0.5(5)	1.4(5)
C7A	24.4(7)	25.8(7)	22.2(7)	-4.9(6)	-2.8(5)	0.2(5)
F1B	78.7(8)	41.7(6)	21.6(5)	-11.0(4)	5.0(5)	-24.2(5)
F2B	44.3(6)	74.7(8)	28.3(5)	1.1(5)	-1.5(4)	-32.3(6)
F3B	87.7(10)	41.4(6)	38.7(6)	9.3(5)	10.4(6)	29.5(6)
N1B	17.2(5)	19.1(5)	20.3(5)	-8.5(4)	-2.3(4)	0.7(4)

Table S11 Anisotropic Displacement Parameters ($Å^2×10^3$) for 31. The Anisotropic displacement factor exponent takes the form: $-2\pi^2[h^2a^{*2}U_{11}+2hka^*b^*U_{12}+…]$.

Atom	U_{11}	U_{22}	U_{33}	U_{23}	U_{13}	U_{12}
C1B	14.5(6)	18.6(6)	20.4(6)	-8.2(5)	-2.7(5)	2.0(4)
C2B	15.2(6)	18.5(6)	21.0(6)	-8.4(5)	-2.9(5)	2.1(5)
C3B	21.0(6)	21.5(6)	19.6(6)	-8.7(5)	-2.8(5)	0.7(5)
C4B	19.2(6)	20.6(6)	21.2(7)	-6.3(5)	-2.2(5)	0.3(5)
C5B	20.4(6)	17.5(6)	25.7(7)	-8.5(5)	-2.2(5)	-1.3(5)
C6B	18.9(6)	20.2(6)	23.7(6)	-11.1(5)	-3.2(5)	0.6(5)
C7B	31.0(7)	21.9(7)	22.0(7)	-5.8(6)	-3.1(5)	-2.8(5)
F1C	49.3(6)	18.2(4)	41.5(5)	-13.0(4)	-17.0(4)	0.7(4)
F2C	37.4(5)	41.8(5)	42.6(5)	-31.6(5)	12.8(4)	-14.4(4)
F3C	27.2(4)	37.9(5)	43.5(5)	-25.8(4)	-14.4(4)	4.7(4)
N1C	17.2(5)	17.9(5)	17.3(5)	-6.1(4)	-2.3(4)	0.1(4)
C1C	18.6(6)	16.1(6)	23.0(6)	-5.4(5)	-2.1(5)	-1.2(5)
C2C	19.8(6)	18.9(6)	17.5(6)	-4.9(5)	-2.0(5)	-0.3(5)
C3C	14.9(6)	17.3(6)	17.6(6)	-6.2(5)	-3.1(4)	1.2(4)
C4C	14.9(6)	17.4(6)	17.7(6)	-6.1(5)	-2.8(4)	1.3(4)
C5C	18.7(6)	20.1(6)	18.7(6)	-8.5(5)	-2.8(5)	0.9(5)
C6C	17.0(6)	18.2(6)	24.2(7)	-9.9(5)	-4.0(5)	1.5(5)
C7C	20.7(6)	20.6(6)	26.0(7)	-10.9(5)	-1.8(5)	-1.0(5)

Table S12 Bond Lengths for 31.

Atom	Atom	Length/Å	Atom	Atom	Length/Å
F1A	C7A	1.3342(17)	C1B	C6B[2]	1.4285(18)
F2A	C7A	1.3343(18)	C2B	C3B	1.4262(18)
F3A	C7A	1.3323(18)	C3B	C4B	1.3642(19)
N1A	C1A	1.3391(19)	C4B	C5B	1.4292(19)
N1A	C2A	1.3433(18)	C4B	C7B	1.5036(19)
C1A	C2A[1]	1.4357(18)	C5B	C6B	1.3611(19)
C1A	C6A[1]	1.4286(19)	F1C	C7C	1.3418(16)
C2A	C3A	1.427(2)	F2C	C7C	1.3314(16)
C3A	C4A	1.359(2)	F3C	C7C	1.3383(16)
C4A	C5A	1.430(2)	N1C	C3C	1.3435(17)
C5A	C6A	1.358(2)	N1C	C4C	1.3441(17)
C5A	C7A	1.5027(19)	C1C	C2C	1.3624(19)
F1B	C7B	1.3243(18)	C1C	C6C[3]	1.4273(19)
F2B	C7B	1.3301(18)	C2C	C3C	1.4294(18)
F3B	C7B	1.3152(19)	C3C	C4C[3]	1.4370(17)
N1B	C1B	1.3446(17)	C4C	C5C	1.4294(18)

Table S12 Bond Lengths for 31.

Atom	Atom	Length/Å	Atom	Atom	Length/Å
N1B	C2B	1.3460(17)	C5C	C6C	1.3641(18)
C1B	C2B[2]	1.4372(18)	C6C	C7C	1.5051(18)

[1]2-X,1-Y,1-Z; [2]3-X,1-Y,-Z; [3]2-X,1-Y,-Z

Table S13 Bond Angles for 31.

Atom	Atom	Atom	Angle/°	Atom	Atom	Atom	Angle/°
C1A	N1A	C2A	116.44(12)	C5B	C4B	C7B	117.52(12)
N1A	C1A	C2A[1]	122.10(13)	C6B	C5B	C4B	120.39(12)
N1A	C1A	C6A[1]	118.70(12)	C5B	C6B	C1B[2]	120.14(12)
C6A[1]	C1A	C2A[1]	119.20(13)	F1B	C7B	F2B	105.74(13)
N1A	C2A	C1A[1]	121.45(13)	F1B	C7B	C4B	113.36(12)
N1A	C2A	C3A	119.13(12)	F2B	C7B	C4B	111.43(12)
C3A	C2A	C1A[1]	119.41(13)	F3B	C7B	F1B	107.45(14)
C4A	C3A	C2A	119.92(13)	F3B	C7B	F2B	106.47(14)
C3A	C4A	C5A	120.37(14)	F3B	C7B	C4B	111.95(12)
C4A	C5A	C7A	117.60(13)	C3C	N1C	C4C	116.31(11)
C6A	C5A	C4A	121.81(13)	C2C	C1C	C6C[3]	120.21(12)
C6A	C5A	C7A	120.58(13)	C1C	C2C	C3C	120.29(12)
C5A	C6A	C1A[1]	119.27(13)	N1C	C3C	C2C	119.11(11)
F1A	C7A	F2A	106.17(13)	N1C	C3C	C4C[3]	121.77(12)
F1A	C7A	C5A	112.13(12)	C2C	C3C	C4C[3]	119.12(12)
F2A	C7A	C5A	111.38(12)	N1C	C4C	C3C[3]	121.92(12)
F3A	C7A	F1A	106.97(12)	N1C	C4C	C5C	118.85(12)
F3A	C7A	F2A	106.88(12)	C5C	C4C	C3C[3]	119.22(12)
F3A	C7A	C5A	112.90(12)	C6C	C5C	C4C	119.41(12)
C1B	N1B	C2B	116.08(11)	C1C[3]	C6C	C7C	118.39(12)
N1B	C1B	C2B[2]	121.90(12)	C5C	C6C	C1C[3]	121.75(12)
N1B	C1B	C6B[2]	118.94(12)	C5C	C6C	C7C	119.81(12)
C6B[2]	C1B	C2B[2]	119.16(12)	F1C	C7C	C6C	112.18(11)
N1B	C2B	C1B[2]	122.02(12)	F2C	C7C	F1C	106.93(11)
N1B	C2B	C3B	118.68(12)	F2C	C7C	F3C	106.36(12)
C3B	C2B	C1B[2]	119.30(12)	F2C	C7C	C6C	113.00(11)
C4B	C3B	C2B	119.46(12)	F3C	C7C	F1C	105.70(11)
C3B	C4B	C5B	121.54(12)	F3C	C7C	C6C	112.17(11)
C3B	C4B	C7B	120.94(13)				

[1]2-X,1-Y,1-Z; [2]3-X,1-Y,-Z; [3]2-X,1-Y,-Z

Table S14 Torsion Angles for 31.

A	B	C	D	Angle/°	A	B	C	D	Angle/°
N1A	C2A	C3A	C4A	-178.96(14)	C3B	C4B	C7B	F1B	1.2(2)
C1A	N1A	C2A	C1A[1]	0.0(2)	C3B	C4B	C7B	F2B	120.33(15)
C1A	N1A	C2A	C3A	-179.62(13)	C3B	C4B	C7B	F3B	-120.56(16)
C1A[1]	C2A	C3A	C4A	1.4(2)	C4B	C5B	C6B	C1B[2]	1.1(2)
C2A	N1A	C1A	C2A[1]	0.0(2)	C5B	C4B	C7B	F1B	-177.97(13)
C2A	N1A	C1A	C6A[1]	179.71(12)	C5B	C4B	C7B	F2B	-58.83(17)
C2A	C3A	C4A	C5A	-1.0(2)	C5B	C4B	C7B	F3B	60.28(18)
C3A	C4A	C5A	C6A	-0.3(2)	C7B	C4B	C5B	C6B	179.36(13)
C3A	C4A	C5A	C7A	178.60(14)	N1C	C4C	C5C	C6C	-179.78(12)
C4A	C5A	C6A	C1A[1]	1.0(2)	C1C	C2C	C3C	N1C	-179.76(12)
C4A	C5A	C7A	F1A	55.06(18)	C1C	C2C	C3C	C4C[3]	0.25(19)
C4A	C5A	C7A	F2A	-63.76(17)	C1C[3]	C6C	C7C	F1C	30.68(17)
C4A	C5A	C7A	F3A	175.98(13)	C1C[3]	C6C	C7C	F2C	151.65(12)
C6A	C5A	C7A	F1A	-126.04(14)	C1C[3]	C6C	C7C	F3C	-88.12(15)
C6A	C5A	C7A	F2A	115.13(16)	C3C	N1C	C4C	C3C[3]	0.00(19)
C6A	C5A	C7A	F3A	-5.12(19)	C3C	N1C	C4C	C5C	-179.87(11)
C7A	C5A	C6A	C1A[1]	-177.83(12)	C3C[3]	C4C	C5C	C6C	0.34(18)
N1B	C2B	C3B	C4B	-178.98(12)	C4C	N1C	C3C	C2C	-179.99(11)
C1B	N1B	C2B	C1B[2]	-0.39(19)	C4C	N1C	C3C	C4C[3]	0.00(19)
C1B	N1B	C2B	C3B	179.75(11)	C4C	C5C	C6C	C1C[3]	-0.20(19)
C1B[2]	C2B	C3B	C4B	1.16(19)	C4C	C5C	C6C	C7C	-177.69(11)
C2B	N1B	C1B	C2B[2]	0.39(19)	C5C	C6C	C7C	F1C	-151.75(12)
C2B	N1B	C1B	C6B[2]	-179.82(11)	C5C	C6C	C7C	F2C	-30.77(18)
C2B	C3B	C4B	C5B	-1.3(2)	C5C	C6C	C7C	F3C	89.45(15)
C2B	C3B	C4B	C7B	179.56(12)	C6C[3]	C1C	C2C	C3C	-0.40(19)
C3B	C4B	C5B	C6B	0.2(2)					

[1]2-X,1-Y,1-Z; [2]3-X,1-Y,-Z; [3]2-X,1-Y,-Z

Table S15 Hydrogen Atom Coordinates (Å×10^{4}) and Isotropic Displacement Parameters (Å^{2}×10^{3}) for 31.

Atom	*x*	*y*	*z*	U(eq)
H3A	11803.28	6373.61	6515.36	33
H4A	14626.37	7409.96	5362.84	32
H6A	13872.49	6156.97	2917.99	28
H3B	13423.5	3648.25	2859.44	25
H5B	12325.82	877.66	1738.51	26

Table S15 Hydrogen Atom Coordinates (Å×10^4) and Isotropic Displacement Parameters (Å^2×10^3) for 31.

Atom	*x*	*y*	*z*	U(eq)
H6B	13524.62	2248.94	-139.12	24
H1C	7382.27	878.34	1374.11	24
H2C	7905.22	2285.23	2203.44	24
H5C	10308.13	6440.8	1804.67	23

Experimental

Single crystals of $C_{14}H_6F_6N_2$ **31** were grown using slow evaporation of chloroform A suitable crystal was selected and analyzed on a **Bruker Photon 2 kappa microsource** diffractometer. The crystal was kept at 110.00(10) K during data collection. Using Olex2,[5] the structure was solved with the SHELXT[6] structure solution program using Intrinsic Phasing and refined with the XL[7] refinement package using Least Squares minimisation.

Crystal structure determination of 31

Crystal Data for $C_{14}H_6F_6N_2$ (M=316.21 g/mol): triclinic, space group P-1 (no. 2), a = 7.19230(10) Å, b = 11.2284(3) Å, c = 12.9596(3) Å, α = 66.282(2)°, β = 79.6040(10)°, γ = 88.7040(10)°, V = 941.01(4) Å^3, Z = 3, T = 110.00(10) K, μ(Cu Kα) = 1.465 mm^{-1}, $Dcalc$ = 1.674 g/cm^3, 28316 reflections measured (7.586° ≤ 2Θ ≤ 156.388°), 3936 unique (R_{int} = 0.0254, R_{sigma} = 0.0103) which were used in all calculations. The final R_1 was 0.0391 (I > 2σ(I)) and wR_2 was 0.1015 (all data).

Refinement model description

Number of restraints - 0, number of constraints - unknown.
Details:
1. Fixed Uiso
At 1.2 times of:
All C(H) groups
2.a Aromatic/amide H refined with riding coordinates:
C3A(H3A), C4A(H4A), C6A(H6A), C3B(H3B), C5B(H5B), C6B(H6B), C1C(H1C), C2C(H2C), C5C(H5C)

X-ray crystallographic data for **33**

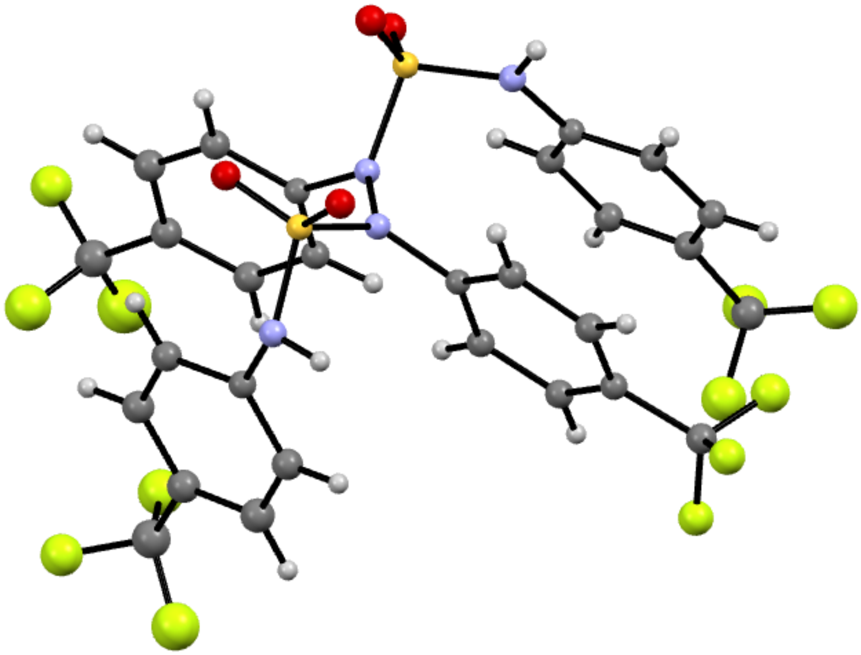

Figure S12. ORTEP drawing of **33** showing thermal ellipsoids at the 50% probability level.

Table S16.
Crystal data and structure refinement for 33.

Identification code	**33**	
Empirical formula	C28 H18 F12 N4 O4 S2	
Formula weight	766.58	
Temperature	110.00 K	
Wavelength	0.71073 Å	
Crystal system	Monoclinic	
Space group	P 1 21/c 1	
Unit cell dimensions	a = 8.1831(6) Å	α
	b = 31.960(2) Å	β = 104.917(2)°.
	c = 11.6634(9) Å	γ = 90°.
Volume	2947.5(4) $Å^3$	
Z	4	
Density (calculated)	1.727 Mg/m^3	
Absorption coefficient	0.302 mm^{-1}	
F(000)	1544	
Crystal size	0.132 x 0.124 x 0.087 mm^3	
Theta range for data collection	1.916 to 28.304°.	
Index ranges	-10<=h<=10, -42<=k<=42, -15<=l<=15	

Reflections collected	70286
Independent reflections	7317 [R(int) = 0.0369]
Completeness to theta = 25.242°	99.9 %
Absorption correction	Semi-empirical from equivalents
Max. and min. transmission	0.7457 and 0.7196
Refinement method	Full-matrix least-squares on F^2
Data / restraints / parameters	7317 / 138 / 479
Goodness-of-fit on F^2	1.042
Final R indices [I>2sigma(I)]	R1 = 0.0366, wR2 = 0.0917
R indices (all data)	R1 = 0.0447, wR2 = 0.0946
Extinction coefficient	n/a
Largest diff. peak and hole	0.443 and -0.497 e.Å^{-3}

Table S17. Atomic coordinates (x 10^4) and equivalent isotropic displacement parameters (Å^{2}x 10^3) for 33. U(eq) is defined as one third of the trace of the orthogonalized U^{ij} tensor.

	x	y	z	U(eq)
S(1)	4164(1)	4207(1)	3996(1)	15(1)
S(2)	8622(1)	4407(1)	5181(1)	15(1)
F(4)	9024(2)	2111(1)	4429(1)	63(1)
F(5)	7073(2)	2238(1)	2843(1)	46(1)
F(6)	9641(1)	2381(1)	2919(1)	37(1)
F(7)	3928(1)	3976(1)	10260(1)	27(1)
F(8)	3057(1)	4605(1)	9801(1)	28(1)
F(9)	5693(1)	4487(1)	10564(1)	33(1)
F(10)	8274(2)	3718(1)	10968(1)	44(1)
F(11)	10952(2)	3671(1)	11298(1)	44(1)
F(12)	9826(2)	4219(1)	11841(1)	43(1)
O(1)	4885(1)	4083(1)	3067(1)	19(1)
O(2)	3486(1)	4619(1)	3999(1)	20(1)
O(3)	10190(1)	4200(1)	5235(1)	21(1)
O(4)	7928(2)	4693(1)	4247(1)	21(1)
N(1)	2642(2)	3898(1)	4102(1)	17(1)
N(2)	5668(2)	4121(1)	5256(1)	15(1)
N(3)	7315(2)	4010(1)	5231(1)	14(1)
N(4)	8724(2)	4656(1)	6397(1)	16(1)
C(1)	2921(2)	3461(1)	4297(1)	17(1)
C(2)	3567(2)	3222(1)	3514(1)	20(1)
C(3)	3828(2)	2798(1)	3722(2)	23(1)
C(4)	3415(2)	2612(1)	4686(2)	24(1)
C(5)	2738(2)	2847(1)	5451(2)	26(1)
C(6)	2496(2)	3274(1)	5260(2)	22(1)
C(7)	3731(3)	2152(1)	4886(2)	33(1)

F(1)	2620(20)	2012(5)	5485(15)	50(3)
F(2)	5225(18)	2082(5)	5600(20)	67(4)
F(3)	3490(30)	1921(8)	3937(14)	48(3)
F(1A)	3047(8)	1932(2)	3897(4)	39(1)
F(2A)	3126(10)	1990(2)	5736(5)	53(1)
F(3A)	5396(4)	2056(1)	5139(6)	46(1)
C(8)	7650(2)	3602(1)	4794(1)	15(1)
C(9)	7878(2)	3265(1)	5566(1)	18(1)
C(10)	8152(2)	2868(1)	5167(1)	21(1)
C(11)	8256(2)	2817(1)	4011(1)	21(1)
C(12)	8109(2)	3158(1)	3251(1)	22(1)
C(13)	7799(2)	3552(1)	3641(1)	19(1)
C(14)	8505(3)	2387(1)	3564(2)	30(1)
C(15)	5270(2)	4172(1)	6389(1)	14(1)
C(16)	4572(2)	4545(1)	6653(1)	18(1)
C(17)	4202(2)	4594(1)	7744(1)	19(1)
C(18)	4568(2)	4275(1)	8575(1)	16(1)
C(19)	5260(2)	3900(1)	8311(1)	18(1)
C(20)	5578(2)	3847(1)	7209(1)	17(1)
C(21)	4305(2)	4335(1)	9795(1)	21(1)
C(22)	9098(2)	4476(1)	7543(1)	15(1)
C(23)	9865(2)	4085(1)	7796(1)	18(1)
C(24)	10072(2)	3914(1)	8918(1)	19(1)
C(25)	9562(2)	4136(1)	9789(1)	18(1)
C(26)	8881(2)	4534(1)	9551(1)	18(1)
C(27)	8641(2)	4704(1)	8431(1)	16(1)
C(28)	9678(2)	3940(1)	10970(2)	26(1)

Table S19. Bond lengths [Å] and angles [°] for 33.

S(1)-O(1)	1.4165(11)
S(1)-O(2)	1.4293(11)
S(1)-N(1)	1.6197(13)
S(1)-N(2)	1.6789(13)
S(2)-O(3)	1.4306(12)
S(2)-O(4)	1.4214(11)
S(2)-N(3)	1.6709(13)
S(2)-N(4)	1.6095(13)
F(4)-C(14)	1.325(2)
F(5)-C(14)	1.342(2)
F(6)-C(14)	1.338(2)
F(7)-C(21)	1.3374(19)
F(8)-C(21)	1.3396(19)
F(9)-C(21)	1.3438(19)
F(10)-C(28)	1.349(2)

F(11)-C(28)	1.330(2)
F(12)-C(28)	1.334(2)
N(1)-H(1)	0.9130
N(1)-C(1)	1.4235(19)
N(2)-N(3)	1.4011(17)
N(2)-C(15)	1.4496(18)
N(3)-C(8)	1.4515(18)
N(4)-H(4)	0.8800
N(4)-C(22)	1.4148(19)
C(1)-C(2)	1.395(2)
C(1)-C(6)	1.393(2)
C(2)-H(2)	0.9500
C(2)-C(3)	1.384(2)
C(3)-H(3)	0.9500
C(3)-C(4)	1.388(2)
C(4)-C(5)	1.387(2)
C(4)-C(7)	1.500(2)
C(5)-H(5)	0.9500
C(5)-C(6)	1.388(2)
C(6)-H(6)	0.9500
C(7)-F(1)	1.357(10)
C(7)-F(2)	1.310(9)
C(7)-F(3)	1.302(11)
C(7)-F(1A)	1.344(4)
C(7)-F(2A)	1.322(4)
C(7)-F(3A)	1.353(4)
C(8)-C(9)	1.386(2)
C(8)-C(13)	1.391(2)
C(9)-H(9)	0.9500
C(9)-C(10)	1.388(2)
C(10)-H(10)	0.9500
C(10)-C(11)	1.383(2)
C(11)-C(12)	1.391(2)
C(11)-C(14)	1.499(2)
C(12)-H(12)	0.9500
C(12)-C(13)	1.385(2)
C(13)-H(13)	0.9500
C(15)-C(16)	1.389(2)
C(15)-C(20)	1.391(2)
C(16)-H(16)	0.9500
C(16)-C(17)	1.391(2)
C(17)-H(17)	0.9500
C(17)-C(18)	1.386(2)
C(18)-C(19)	1.393(2)
C(18)-C(21)	1.504(2)
C(19)-H(19)	0.9500

C(19)-C(20)	1.386(2)
C(20)-H(20)	0.9500
C(22)-C(23)	1.397(2)
C(22)-C(27)	1.394(2)
C(23)-H(23)	0.9500
C(23)-C(24)	1.387(2)
C(24)-H(24)	0.9500
C(24)-C(25)	1.388(2)
C(25)-C(26)	1.390(2)
C(25)-C(28)	1.493(2)
C(26)-H(26)	0.9500
C(26)-C(27)	1.380(2)
C(27)-H(27)	0.9500

O(1)-S(1)-O(2)	119.90(7)
O(1)-S(1)-N(1)	111.54(7)
O(1)-S(1)-N(2)	105.53(7)
O(2)-S(1)-N(1)	104.82(7)
O(2)-S(1)-N(2)	110.21(7)
N(1)-S(1)-N(2)	103.78(7)
O(3)-S(2)-N(3)	102.86(6)
O(3)-S(2)-N(4)	110.31(7)
O(4)-S(2)-O(3)	120.45(7)
O(4)-S(2)-N(3)	112.39(7)
O(4)-S(2)-N(4)	106.14(7)
N(4)-S(2)-N(3)	103.52(6)
S(1)-N(1)-H(1)	107.3
C(1)-N(1)-S(1)	121.07(11)
C(1)-N(1)-H(1)	111.6
N(3)-N(2)-S(1)	121.02(9)
N(3)-N(2)-C(15)	119.37(12)
C(15)-N(2)-S(1)	119.60(10)
N(2)-N(3)-S(2)	115.88(9)
N(2)-N(3)-C(8)	120.30(12)
C(8)-N(3)-S(2)	119.31(10)
S(2)-N(4)-H(4)	117.3
C(22)-N(4)-S(2)	125.46(10)
C(22)-N(4)-H(4)	117.3
C(2)-C(1)-N(1)	120.30(14)
C(6)-C(1)-N(1)	119.12(14)
C(6)-C(1)-C(2)	120.56(14)
C(1)-C(2)-H(2)	120.4
C(3)-C(2)-C(1)	119.26(15)
C(3)-C(2)-H(2)	120.4
C(2)-C(3)-H(3)	119.9
C(2)-C(3)-C(4)	120.16(15)

C(4)-C(3)-H(3)	119.9
C(3)-C(4)-C(7)	118.50(16)
C(5)-C(4)-C(3)	120.66(15)
C(5)-C(4)-C(7)	120.84(16)
C(4)-C(5)-H(5)	120.2
C(4)-C(5)-C(6)	119.59(16)
C(6)-C(5)-H(5)	120.2
C(1)-C(6)-H(6)	120.1
C(5)-C(6)-C(1)	119.75(15)
C(5)-C(6)-H(6)	120.1
F(1)-C(7)-C(4)	106.9(7)
F(2)-C(7)-C(4)	111.2(7)
F(2)-C(7)-F(1)	104.8(8)
F(3)-C(7)-C(4)	116.1(12)
F(3)-C(7)-F(1)	106.0(10)
F(3)-C(7)-F(2)	111.0(9)
F(1A)-C(7)-C(4)	111.2(3)
F(1A)-C(7)-F(3A)	104.0(3)
F(2A)-C(7)-C(4)	114.7(3)
F(2A)-C(7)-F(1A)	106.6(4)
F(2A)-C(7)-F(3A)	107.3(3)
F(3A)-C(7)-C(4)	112.3(2)
C(9)-C(8)-N(3)	118.47(13)
C(9)-C(8)-C(13)	120.54(13)
C(13)-C(8)-N(3)	120.96(13)
C(8)-C(9)-H(9)	120.0
C(8)-C(9)-C(10)	119.91(14)
C(10)-C(9)-H(9)	120.0
C(9)-C(10)-H(10)	120.3
C(11)-C(10)-C(9)	119.49(15)
C(11)-C(10)-H(10)	120.3
C(10)-C(11)-C(12)	120.69(14)
C(10)-C(11)-C(14)	119.77(15)
C(12)-C(11)-C(14)	119.53(15)
C(11)-C(12)-H(12)	120.1
C(13)-C(12)-C(11)	119.83(14)
C(13)-C(12)-H(12)	120.1
C(8)-C(13)-H(13)	120.3
C(12)-C(13)-C(8)	119.42(14)
C(12)-C(13)-H(13)	120.3
F(4)-C(14)-F(5)	107.26(17)
F(4)-C(14)-F(6)	106.52(16)
F(4)-C(14)-C(11)	113.00(15)
F(5)-C(14)-C(11)	111.67(15)
F(6)-C(14)-F(5)	105.26(14)
F(6)-C(14)-C(11)	112.63(16)

C(16)-C(15)-N(2)	120.03(13)
C(16)-C(15)-C(20)	120.05(13)
C(20)-C(15)-N(2)	119.92(13)
C(15)-C(16)-H(16)	120.1
C(15)-C(16)-C(17)	119.88(14)
C(17)-C(16)-H(16)	120.1
C(16)-C(17)-H(17)	120.1
C(18)-C(17)-C(16)	119.87(14)
C(18)-C(17)-H(17)	120.1
C(17)-C(18)-C(19)	120.34(14)
C(17)-C(18)-C(21)	120.68(14)
C(19)-C(18)-C(21)	118.92(14)
C(18)-C(19)-H(19)	120.2
C(20)-C(19)-C(18)	119.65(14)
C(20)-C(19)-H(19)	120.2
C(15)-C(20)-H(20)	119.9
C(19)-C(20)-C(15)	120.14(14)
C(19)-C(20)-H(20)	119.9
F(7)-C(21)-F(8)	107.09(13)
F(7)-C(21)-F(9)	106.42(13)
F(7)-C(21)-C(18)	112.29(13)
F(8)-C(21)-F(9)	106.14(13)
F(8)-C(21)-C(18)	112.45(14)
F(9)-C(21)-C(18)	112.01(13)
C(23)-C(22)-N(4)	122.73(13)
C(27)-C(22)-N(4)	117.09(13)
C(27)-C(22)-C(23)	120.16(14)
C(22)-C(23)-H(23)	120.3
C(24)-C(23)-C(22)	119.33(14)
C(24)-C(23)-H(23)	120.3
C(23)-C(24)-H(24)	119.9
C(23)-C(24)-C(25)	120.26(14)
C(25)-C(24)-H(24)	119.9
C(24)-C(25)-C(26)	120.15(14)
C(24)-C(25)-C(28)	120.12(14)
C(26)-C(25)-C(28)	119.69(15)
C(25)-C(26)-H(26)	120.0
C(27)-C(26)-C(25)	120.02(14)
C(27)-C(26)-H(26)	120.0
C(22)-C(27)-H(27)	120.0
C(26)-C(27)-C(22)	119.93(14)
C(26)-C(27)-H(27)	120.0
F(10)-C(28)-C(25)	111.54(14)
F(11)-C(28)-F(10)	105.30(15)
F(11)-C(28)-F(12)	107.63(14)
F(11)-C(28)-C(25)	113.28(14)

F(12)-C(28)-F(10)	105.25(15)
F(12)-C(28)-C(25)	113.21(14)

Symmetry transformations used to generate equivalent atoms:

Table S20. Anisotropic displacement parameters ($Å^2$x 10^3) for 33. The anisotropic displacement factor exponent takes the form: -2□^{2}[h^2 $a^{*2}U^{11}$ + ... + 2 h k a* b* U^{12}]

	U^{11}	U^{22}	U^{33}	U^{23}	U^{13}	U^{12}
S(1)	18(1)	13(1)	14(1)	2(1)	5(1)	4(1)
S(2)	18(1)	13(1)	18(1)	1(1)	9(1)	1(1)
F(4)	130(1)	23(1)	39(1)	3(1)	25(1)	33(1)
F(5)	44(1)	34(1)	64(1)	-30(1)	19(1)	-8(1)
F(6)	36(1)	37(1)	39(1)	-17(1)	12(1)	12(1)
F(7)	37(1)	29(1)	21(1)	1(1)	15(1)	-3(1)
F(8)	32(1)	30(1)	29(1)	-5(1)	18(1)	4(1)
F(9)	28(1)	52(1)	21(1)	-14(1)	8(1)	-14(1)
F(10)	44(1)	55(1)	32(1)	17(1)	11(1)	-12(1)
F(11)	50(1)	52(1)	30(1)	19(1)	10(1)	27(1)
F(12)	71(1)	38(1)	18(1)	0(1)	6(1)	7(1)
O(1)	23(1)	21(1)	14(1)	2(1)	7(1)	4(1)
O(2)	24(1)	15(1)	20(1)	3(1)	5(1)	6(1)
O(3)	20(1)	20(1)	27(1)	0(1)	13(1)	2(1)
O(4)	29(1)	16(1)	20(1)	5(1)	10(1)	2(1)
N(1)	19(1)	15(1)	18(1)	0(1)	6(1)	2(1)
N(2)	15(1)	18(1)	14(1)	1(1)	6(1)	4(1)
N(3)	15(1)	12(1)	19(1)	0(1)	8(1)	2(1)
N(4)	22(1)	10(1)	18(1)	1(1)	6(1)	2(1)
C(1)	16(1)	14(1)	19(1)	-1(1)	2(1)	1(1)
C(2)	23(1)	18(1)	19(1)	-1(1)	5(1)	1(1)
C(3)	24(1)	18(1)	25(1)	-5(1)	5(1)	1(1)
C(4)	25(1)	15(1)	30(1)	1(1)	3(1)	1(1)
C(5)	29(1)	22(1)	26(1)	5(1)	8(1)	-2(1)
C(6)	22(1)	20(1)	23(1)	0(1)	7(1)	1(1)
C(7)	36(1)	19(1)	41(1)	4(1)	7(1)	1(1)
F(1)	63(5)	15(3)	71(6)	19(3)	15(5)	-2(4)
F(2)	55(5)	27(4)	93(8)	18(6)	-28(5)	9(4)
F(3)	57(8)	23(4)	63(4)	-3(3)	16(4)	15(6)
F(1A)	41(2)	16(1)	55(1)	-6(1)	5(1)	3(2)
F(2A)	92(3)	24(1)	55(2)	18(1)	40(2)	13(2)
F(3A)	35(1)	23(1)	73(2)	6(1)	-1(1)	9(1)
C(8)	16(1)	13(1)	16(1)	-2(1)	6(1)	3(1)
C(9)	23(1)	16(1)	15(1)	0(1)	8(1)	4(1)
C(10)	29(1)	14(1)	20(1)	1(1)	8(1)	4(1)
C(11)	26(1)	17(1)	21(1)	-4(1)	6(1)	5(1)

C(12)	29(1)	23(1)	15(1)	-2(1)	8(1)	6(1)
C(13)	25(1)	18(1)	17(1)	3(1)	9(1)	5(1)
C(14)	44(1)	21(1)	26(1)	-6(1)	9(1)	7(1)
C(15)	14(1)	16(1)	14(1)	-1(1)	6(1)	-1(1)
C(16)	21(1)	14(1)	18(1)	1(1)	5(1)	2(1)
C(17)	22(1)	16(1)	21(1)	-3(1)	8(1)	3(1)
C(18)	15(1)	20(1)	16(1)	-2(1)	6(1)	-3(1)
C(19)	22(1)	17(1)	17(1)	2(1)	8(1)	-1(1)
C(20)	21(1)	14(1)	19(1)	1(1)	9(1)	2(1)
C(21)	21(1)	24(1)	19(1)	-4(1)	8(1)	-5(1)
C(22)	13(1)	14(1)	19(1)	1(1)	4(1)	-1(1)
C(23)	18(1)	15(1)	21(1)	-1(1)	6(1)	2(1)
C(24)	17(1)	15(1)	23(1)	2(1)	2(1)	2(1)
C(25)	16(1)	20(1)	18(1)	2(1)	2(1)	-1(1)
C(26)	15(1)	20(1)	20(1)	-2(1)	4(1)	-1(1)
C(27)	15(1)	13(1)	21(1)	-1(1)	4(1)	0(1)
C(28)	28(1)	28(1)	21(1)	3(1)	5(1)	4(1)

Table S21. Hydrogen coordinates (x 10^4) and isotropic displacement parameters (Å^{2}x 10^3) for 33.

	x	y	z	U(eq)
H(1)	2139	4013	4640	25
H(4)	8531	4928	6344	19
H(2)	3826	3349	2845	24
H(3)	4291	2633	3204	27
H(5)	2441	2717	6101	31
H(6)	2043	3438	5784	26
H(9)	7847	3305	6368	21
H(10)	8267	2634	5684	25
H(12)	8221	3121	2467	26
H(13)	7688	3786	3125	23
H(16)	4347	4766	6089	21
H(17)	3699	4846	7919	23
H(19)	5512	3682	8883	22
H(20)	6008	3588	7014	21
H(23)	10241	3936	7205	21
H(24)	10566	3645	9091	23
H(26)	8580	4691	10159	22
H(27)	8166	4975	8266	20

Table S22. Torsion angles [°] for 33.

S(1)-N(1)-C(1)-C(2)	-56.82(19)
S(1)-N(1)-C(1)-C(6)	124.93(14)
S(1)-N(2)-N(3)-S(2)	86.42(12)
S(1)-N(2)-N(3)-C(8)	-69.60(16)
S(1)-N(2)-C(15)-C(16)	-53.83(18)
S(1)-N(2)-C(15)-C(20)	125.75(13)
S(2)-N(3)-C(8)-C(9)	121.45(13)
S(2)-N(3)-C(8)-C(13)	-56.38(18)
S(2)-N(4)-C(22)-C(23)	17.8(2)
S(2)-N(4)-C(22)-C(27)	-161.11(11)
O(1)-S(1)-N(1)-C(1)	57.94(13)
O(1)-S(1)-N(2)-N(3)	8.40(13)
O(1)-S(1)-N(2)-C(15)	-173.17(11)
O(2)-S(1)-N(1)-C(1)	-170.86(11)
O(2)-S(1)-N(2)-N(3)	-122.41(11)
O(2)-S(1)-N(2)-C(15)	56.02(13)
O(3)-S(2)-N(3)-N(2)	176.70(10)
O(3)-S(2)-N(3)-C(8)	-27.03(13)
O(3)-S(2)-N(4)-C(22)	-57.98(14)
O(4)-S(2)-N(3)-N(2)	-52.29(12)
O(4)-S(2)-N(3)-C(8)	103.98(12)
O(4)-S(2)-N(4)-C(22)	169.98(12)
N(1)-S(1)-N(2)-N(3)	125.82(11)
N(1)-S(1)-N(2)-C(15)	-55.75(12)
N(1)-C(1)-C(2)-C(3)	-179.99(14)
N(1)-C(1)-C(6)-C(5)	179.05(15)
N(2)-S(1)-N(1)-C(1)	-55.22(13)
N(2)-N(3)-C(8)-C(9)	-83.35(18)
N(2)-N(3)-C(8)-C(13)	98.82(17)
N(2)-C(15)-C(16)-C(17)	-179.60(14)
N(2)-C(15)-C(20)-C(19)	177.58(14)
N(3)-S(2)-N(4)-C(22)	51.47(14)
N(3)-N(2)-C(15)-C(16)	124.63(15)
N(3)-N(2)-C(15)-C(20)	-55.80(19)
N(3)-C(8)-C(9)-C(10)	178.10(14)
N(3)-C(8)-C(13)-C(12)	-179.59(15)
N(4)-S(2)-N(3)-N(2)	61.80(11)
N(4)-S(2)-N(3)-C(8)	-141.93(11)
N(4)-C(22)-C(23)-C(24)	-175.04(14)
N(4)-C(22)-C(27)-C(26)	176.21(14)
C(1)-C(2)-C(3)-C(4)	1.4(2)
C(2)-C(1)-C(6)-C(5)	0.8(2)
C(2)-C(3)-C(4)-C(5)	0.0(3)
C(2)-C(3)-C(4)-C(7)	-179.46(16)

C(3)-C(4)-C(5)-C(6)	-1.0(3)
C(3)-C(4)-C(7)-F(1)	-153.7(9)
C(3)-C(4)-C(7)-F(2)	92.4(15)
C(3)-C(4)-C(7)-F(3)	-35.7(11)
C(3)-C(4)-C(7)-F(1A)	-51.5(4)
C(3)-C(4)-C(7)-F(2A)	-172.5(4)
C(3)-C(4)-C(7)-F(3A)	64.6(4)
C(4)-C(5)-C(6)-C(1)	0.6(3)
C(5)-C(4)-C(7)-F(1)	26.8(9)
C(5)-C(4)-C(7)-F(2)	-87.0(15)
C(5)-C(4)-C(7)-F(3)	144.8(11)
C(5)-C(4)-C(7)-F(1A)	129.0(4)
C(5)-C(4)-C(7)-F(2A)	8.0(5)
C(5)-C(4)-C(7)-F(3A)	-114.9(4)
C(6)-C(1)-C(2)-C(3)	-1.8(2)
C(7)-C(4)-C(5)-C(6)	178.47(17)
C(8)-C(9)-C(10)-C(11)	2.4(2)
C(9)-C(8)-C(13)-C(12)	2.6(2)
C(9)-C(10)-C(11)-C(12)	0.7(3)
C(9)-C(10)-C(11)-C(14)	-178.34(16)
C(10)-C(11)-C(12)-C(13)	-2.2(3)
C(10)-C(11)-C(14)-F(4)	-16.1(3)
C(10)-C(11)-C(14)-F(5)	104.90(19)
C(10)-C(11)-C(14)-F(6)	-136.90(17)
C(11)-C(12)-C(13)-C(8)	0.5(3)
C(12)-C(11)-C(14)-F(4)	164.79(18)
C(12)-C(11)-C(14)-F(5)	-74.2(2)
C(12)-C(11)-C(14)-F(6)	44.0(2)
C(13)-C(8)-C(9)-C(10)	-4.1(2)
C(14)-C(11)-C(12)-C(13)	176.93(17)
C(15)-N(2)-N(3)-S(2)	-92.02(14)
C(15)-N(2)-N(3)-C(8)	111.97(15)
C(15)-C(16)-C(17)-C(18)	1.5(2)
C(16)-C(15)-C(20)-C(19)	-2.8(2)
C(16)-C(17)-C(18)-C(19)	-1.9(2)
C(16)-C(17)-C(18)-C(21)	175.15(15)
C(17)-C(18)-C(19)-C(20)	-0.1(2)
C(17)-C(18)-C(21)-F(7)	150.11(15)
C(17)-C(18)-C(21)-F(8)	29.2(2)
C(17)-C(18)-C(21)-F(9)	-90.20(19)
C(18)-C(19)-C(20)-C(15)	2.5(2)
C(19)-C(18)-C(21)-F(7)	-32.8(2)
C(19)-C(18)-C(21)-F(8)	-153.70(14)
C(19)-C(18)-C(21)-F(9)	86.86(18)
C(20)-C(15)-C(16)-C(17)	0.8(2)
C(21)-C(18)-C(19)-C(20)	-177.21(14)

C(22)-C(23)-C(24)-C(25)	-1.6(2)
C(23)-C(22)-C(27)-C(26)	-2.8(2)
C(23)-C(24)-C(25)-C(26)	-1.7(2)
C(23)-C(24)-C(25)-C(28)	175.79(15)
C(24)-C(25)-C(26)-C(27)	2.8(2)
C(24)-C(25)-C(28)-F(10)	-87.35(19)
C(24)-C(25)-C(28)-F(11)	31.2(2)
C(24)-C(25)-C(28)-F(12)	154.15(15)
C(25)-C(26)-C(27)-C(22)	-0.6(2)
C(26)-C(25)-C(28)-F(10)	90.16(19)
C(26)-C(25)-C(28)-F(11)	-151.25(16)
C(26)-C(25)-C(28)-F(12)	-28.3(2)
C(27)-C(22)-C(23)-C(24)	3.9(2)
C(28)-C(25)-C(26)-C(27)	-174.67(15)

Symmetry transformations used to generate equivalent atoms:

Note:

1. The crystal used had satellite crystals. To resolve the peaks, the data was collected at a crystal to detector distance of 100 mm.
2. Elongated ellipsoids and nearby residual electron density peaks on F1, F2, and F3 suggested disorder which was modeled between two positions each with an occupancy ratio of 0.75:0.25. Appropriate restraints and constraints were added to keep the bond distances, angles, and thermal ellipsoids meaningful.

Data Collection

A Leica MZ 75 microscope was used to identify a suitable colorless block with very well defined faces with dimensions (max, intermediate, and min) 0.132 x 0.124 x 0.087 mm^3 from a representative sample of crystals of the same habit. The crystal mounted on a nylon loop was then placed in a cold nitrogen stream (Oxford) maintained at 110 K.

A BRUKER Quest X-ray (fixed-Chi geometry) diffractometer with a PHOTON III detector was employed for crystal screening, unit cell determination, and data collection. The goniometer was controlled using the APEX3 software suite.[1] The sample was optically centered with the aid of a video camera such that no translations were observed as the crystal was rotated through all positions. The X-ray radiation employed was generated from a Mo-Iμs X-ray tube (K_α = 0.71073Å).

45 data frames were taken at widths of 1°. These reflections were used to determine the unit cell. The unit cell was verified by examination of the *h k l* overlays on several frames of data. No super-cell or erroneous reflections were observed.

After careful examination of the unit cell, an extended data collection procedure (9 sets) was initiated using omega and phi scans.

Data Reduction, Structure Solution, and Refinement

Integrated intensity information for each reflection was obtained by reduction of the data frames with the program APEX3.[8] The integration method employed a three dimensional profiling algorithm and all data were corrected for Lorentz and polarization factors, as well as for crystal

decay effects. Finally, the data was merged and scaled to produce a suitable data set. The absorption correction program SADABS[9] was employed to correct the data for absorption effects. Systematic reflection conditions and statistical tests of the data suggested the space group $P2_1/c$. A solution was obtained readily using XT/XS in APEX3.[6–8,10] Hydrogen atoms were placed in idealized positions and were set riding on the respective parent atoms. All non-hydrogen atoms were refined with anisotropic thermal parameters. Elongated ellipsoids and nearby residual electron density peaks on F1, F2, and F3 suggested disorder which was modeled between two positions each with an occupancy ratio of 0.75:0.25. Appropriate restraints and constraints were added to keep the bond distances, angles, and thermal ellipsoids meaningful. Absence of additional symmetry and voids were confirmed using PLATON (ADDSYM). The structure was refined (weighted least squares refinement on F^2) to convergence.[5–]
Olex2 was employed for the final data presentation and structure plots.[5]

References:

(1) Kulow, R. W.; Wu, J. W.; Kim, C.; Michaudel, Q. Synthesis of unsymmetrical sulfamides and polysulfamides via SuFEx click chemistry. *Chem. Sci.* **2020**, *11*, 7807–7812.
(2) Pavlishchuk, V. V.; Addison, A. W. Conversion constants for redox potentials measured versus different reference electrodes in acetonitrile solutions at 25°C. *Inorg. Chim. Acta.* **2000**, *298*, 97–102.
(3) Saveant, J. M. Electron transfer, bond breaking, and bond formation. *Acc. Chem. Res.* **1993**, *26*, 455–461.
(4) Allen J. Bard, L. R. F. Electrochemical Methods: Fundamentals and Applications. Vol. 2nd Edition. Eds John Wiley & Sons, Inc., **2000**.
(5) Dolomanov, O. V, Bourhis, L. J., Gildea, R. J., Howard, J. A. K., and Puschmann, H. "OLEX2: A Complete Structure Solution, Refinement and Analysis Program", *J. Appl. Cryst.* **2009***, 42*, 339–341.
(6) Sheldrick, G.M. SHELXT - integrated space-group and crystal-structure determination *Acta Cryst.* **2015**, *A71*, 3–8.
(7) Sheldrick, G.M. Crystal Structure Refinement with SHELXL. *Acta Cryst.* **2015**, *C71*, 3–8.
(8) APEX3 "Program for Data Collection on Area Detectors" BRUKER AXS Inc., 5465 East Cheryl Parkway, Madison, WI 53711-5373 USA
(9) SADABS, Sheldrick, G.M. "Program for Absorption Correction of Area Detector Frames", BRUKER AXS Inc., 5465 East Cheryl Parkway, Madison, WI 53711-5373 USA.
(10) Sheldrick, G.M. A Short History of SHELX. *Acta Cryst.* **2008**, *A64*, 112–122.

NMR Spectra

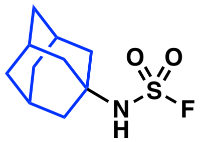

S1
(^{1}H, 400 MHz, $CDCl_3$)

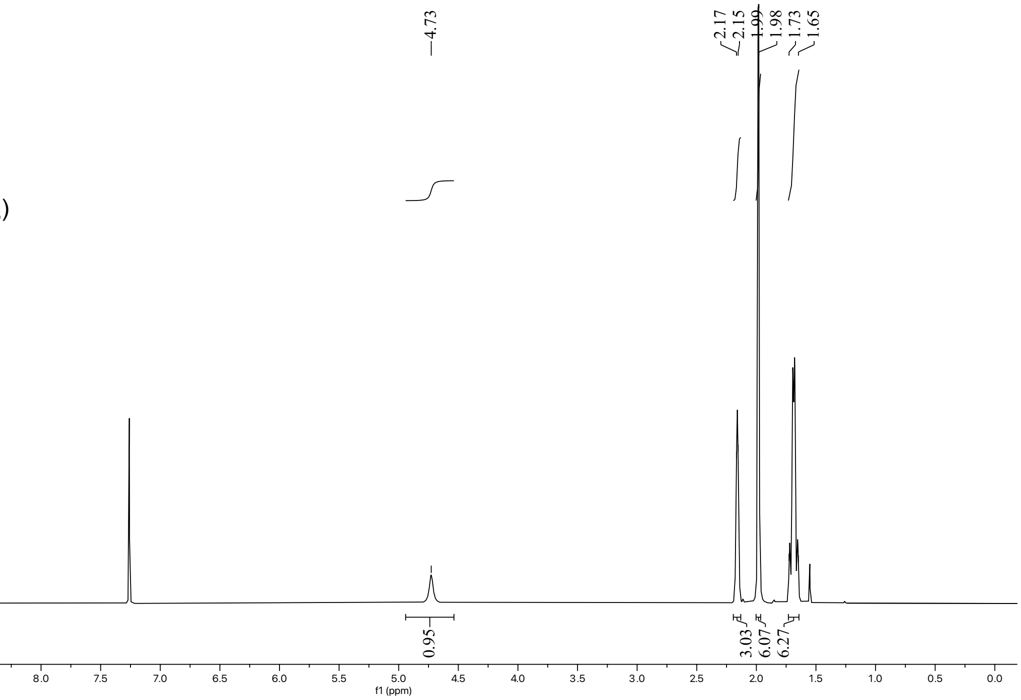

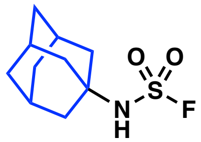

S1
(^{13}C, 126 MHz, $CDCl_3$)

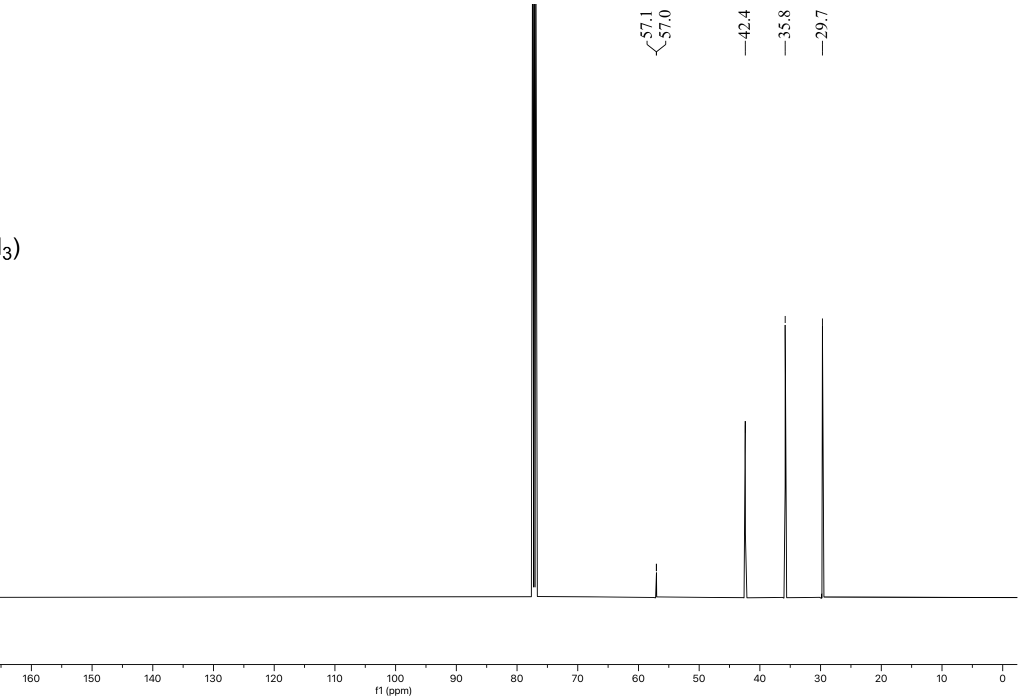

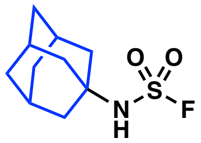

S1

(^{19}F, 470 MHz, $CDCl_3$)

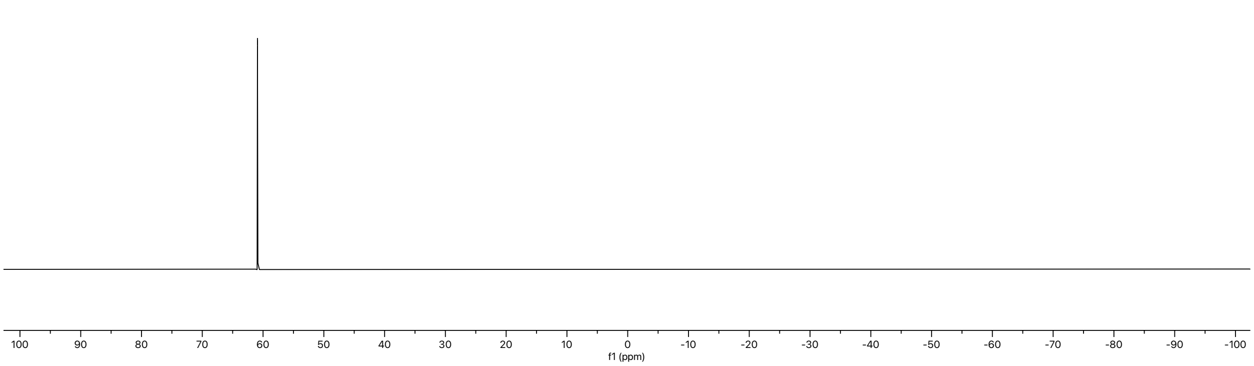

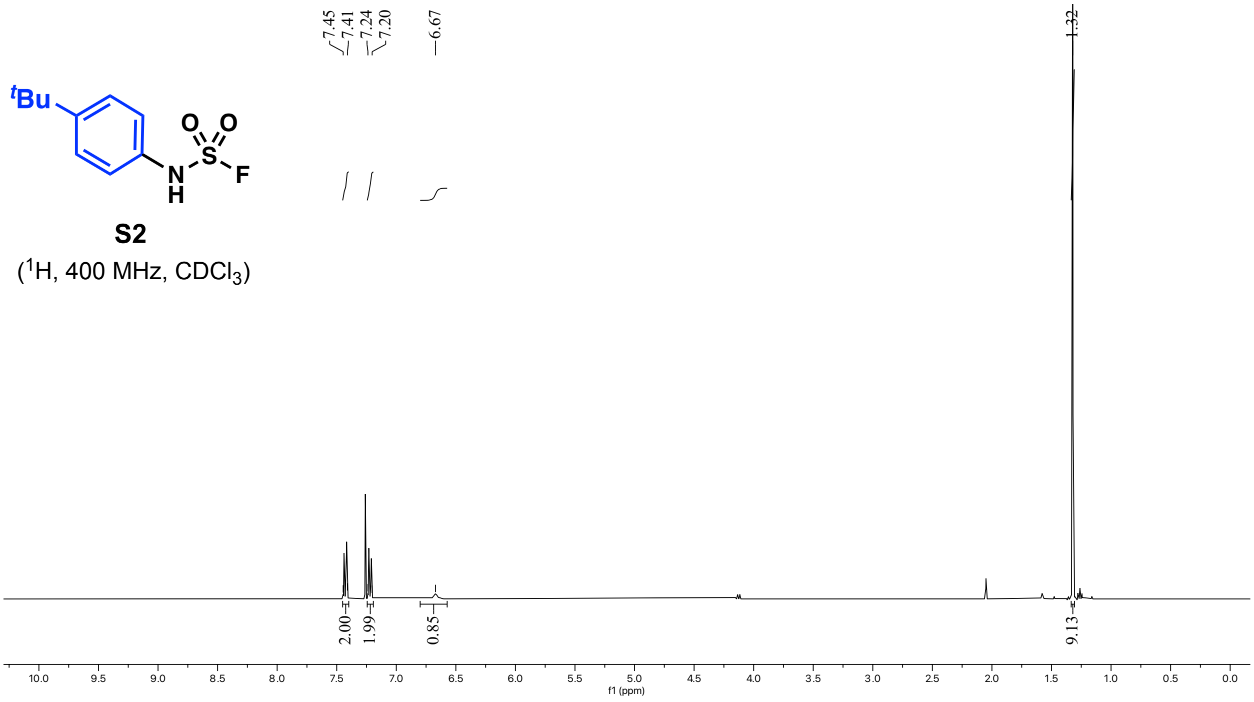

S2
(^{1}H, 400 MHz, $CDCl_3$)

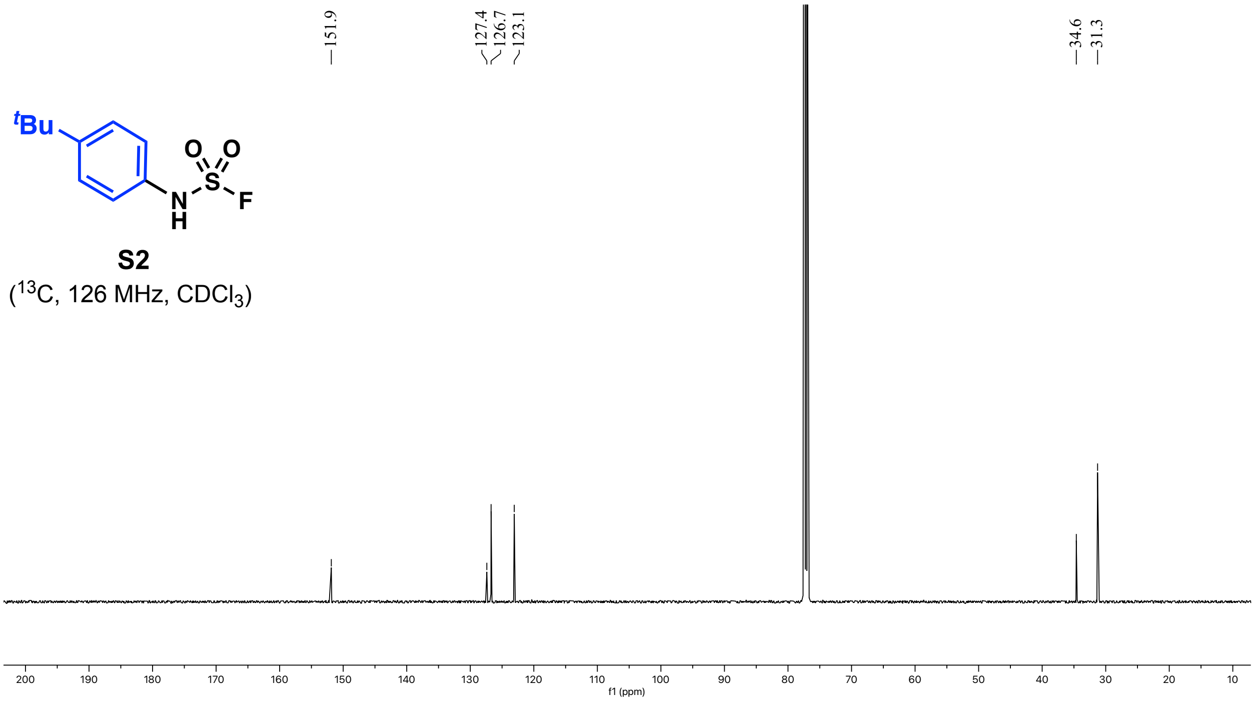

S2
(^{13}C, 126 MHz, $CDCl_3$)

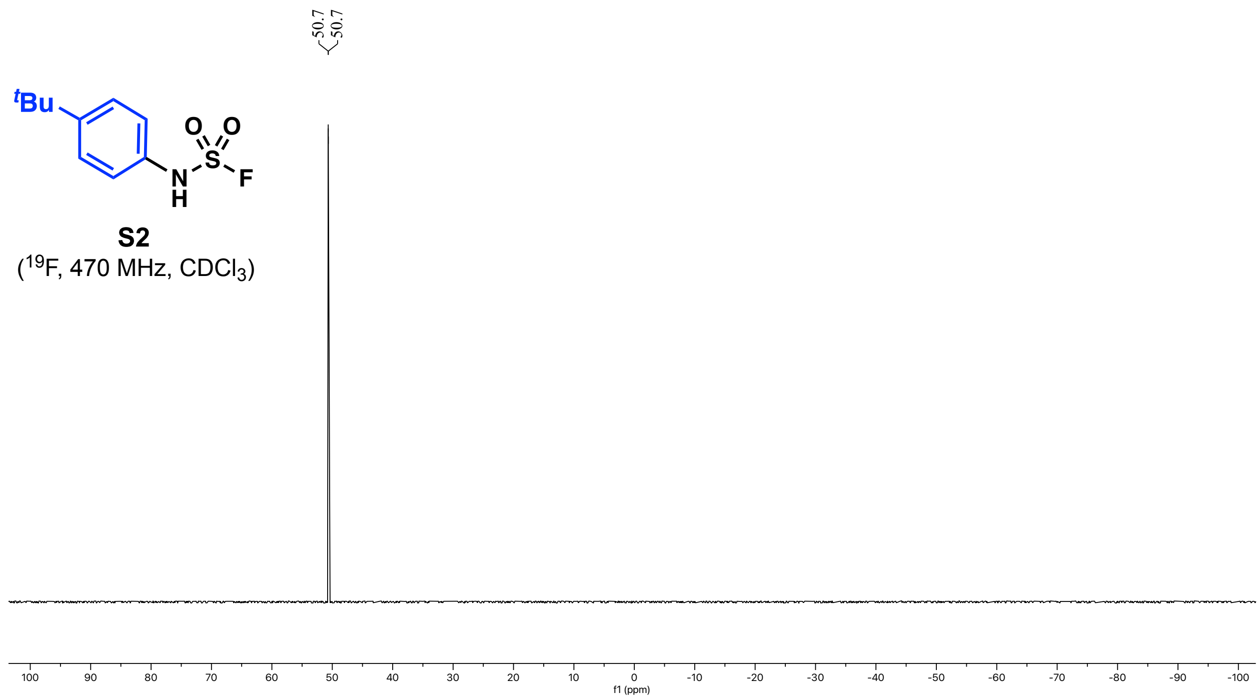
50.7
50.7
tBu
O O
S
N
H
F
S2
(19F, 470 MHz, CDCl3)
100 90 80 70 60 50 40 30 20 10 0 -10 -20 -30 -40 -50 -60 -70 -80 -90 -100
f1 (ppm)

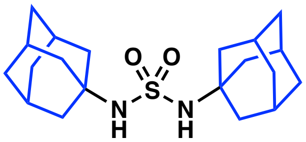

1
(^{1}H, 400 MHz, $CDCl_3$)

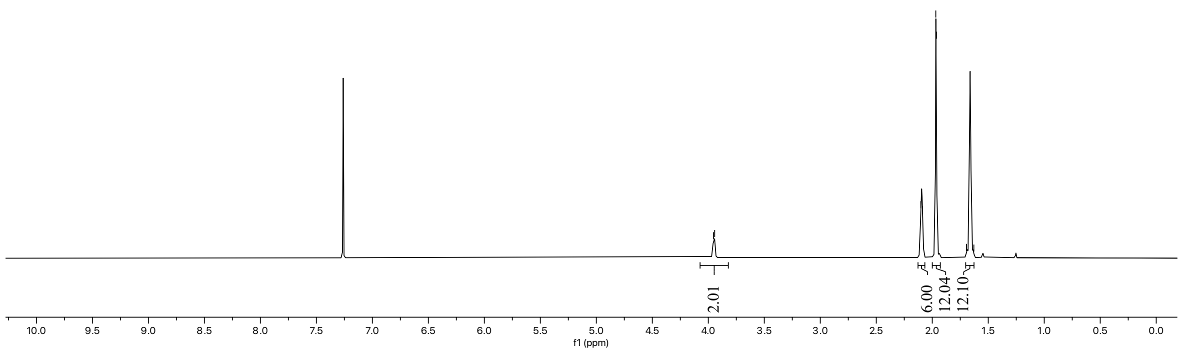

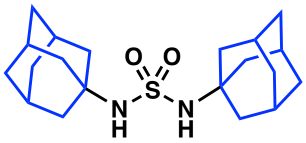

1
(^{13}C, 126 MHz, $CDCl_3$)

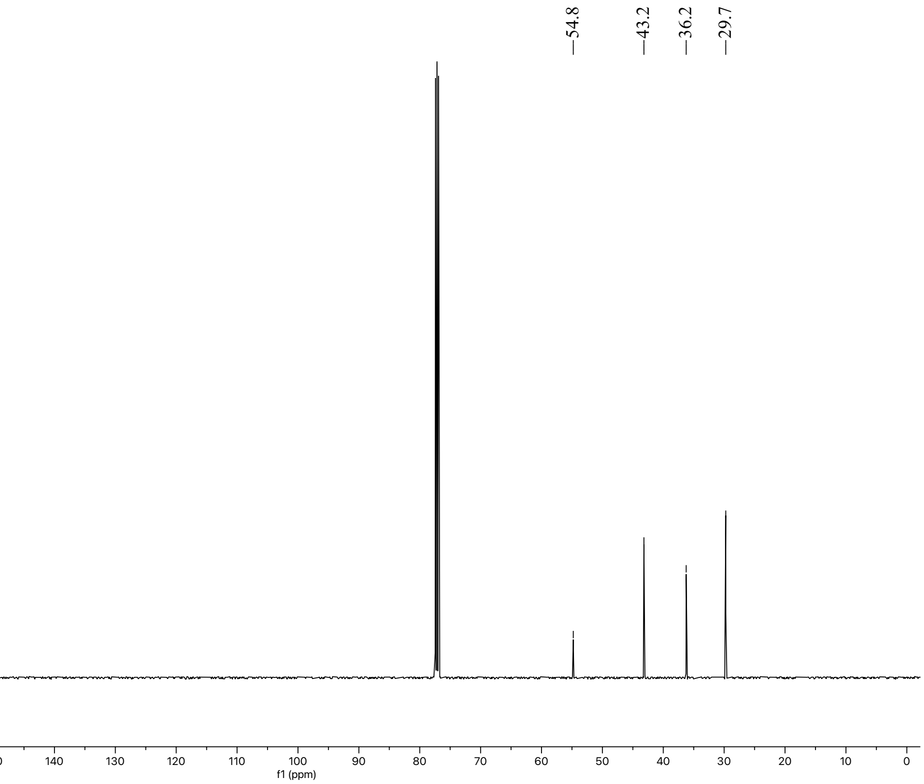

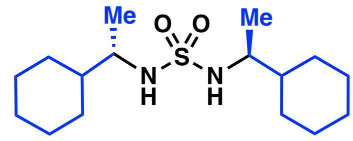

S5
(^{1}H, 400 MHz, $CDCl_3$)

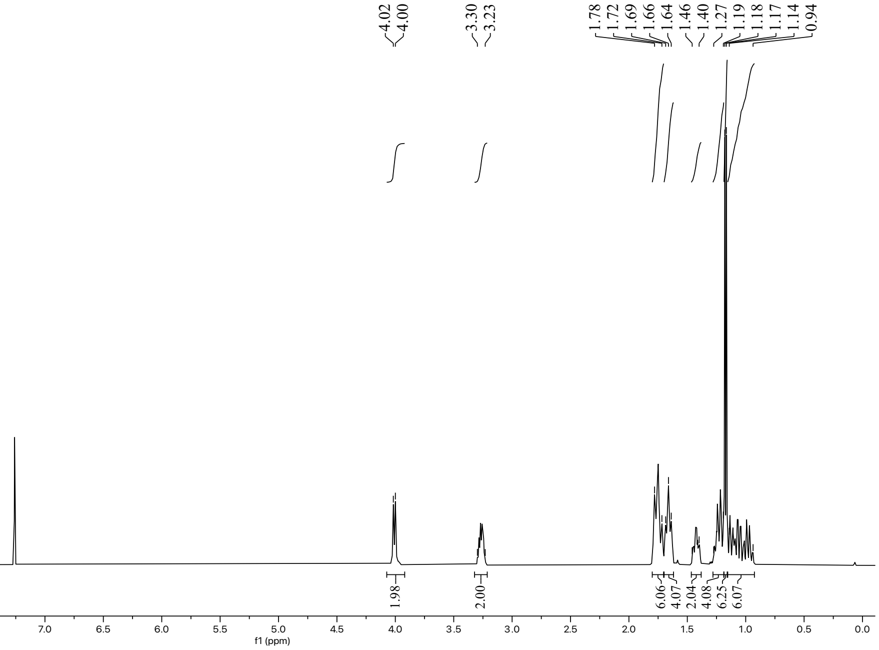

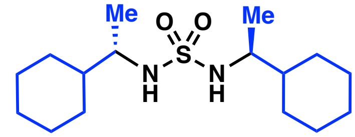

S5
(^{13}C, 126 MHz, $CDCl_3$)

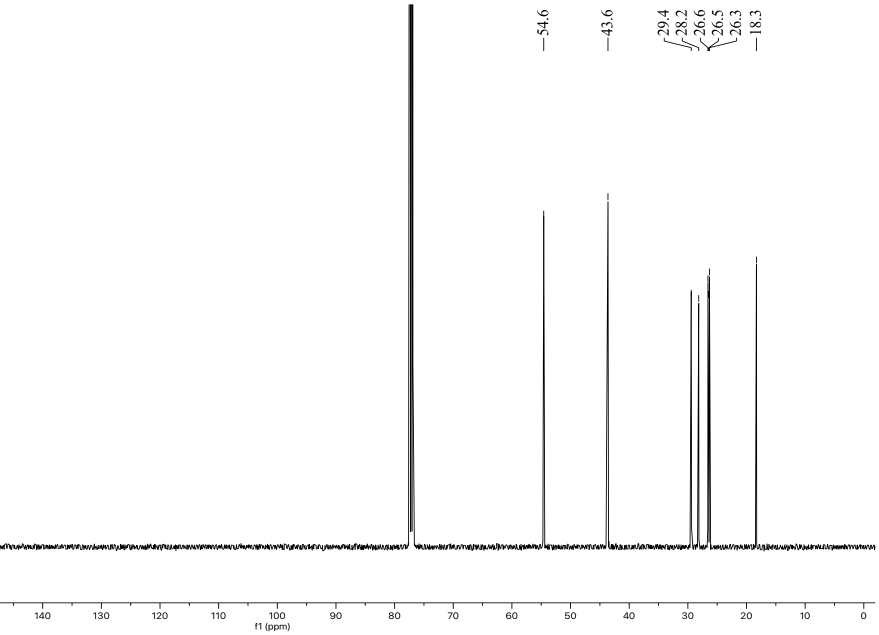

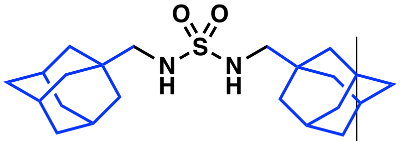

S8

(^{1}H, 400 MHz, $CDCl_3$)

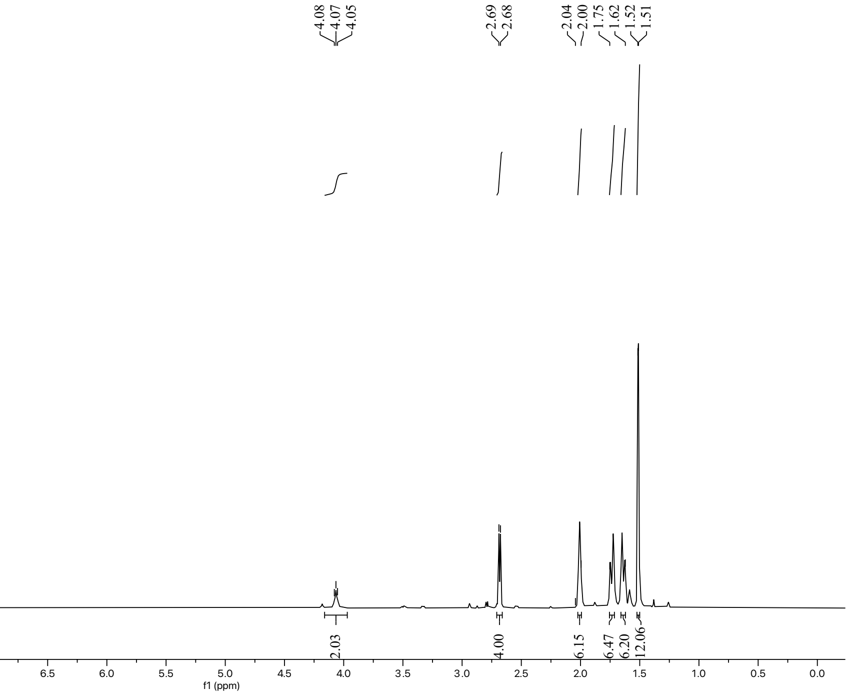

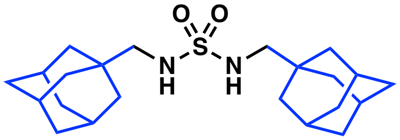

S8

(^{13}C, 126 MHz, $CDCl_3$)

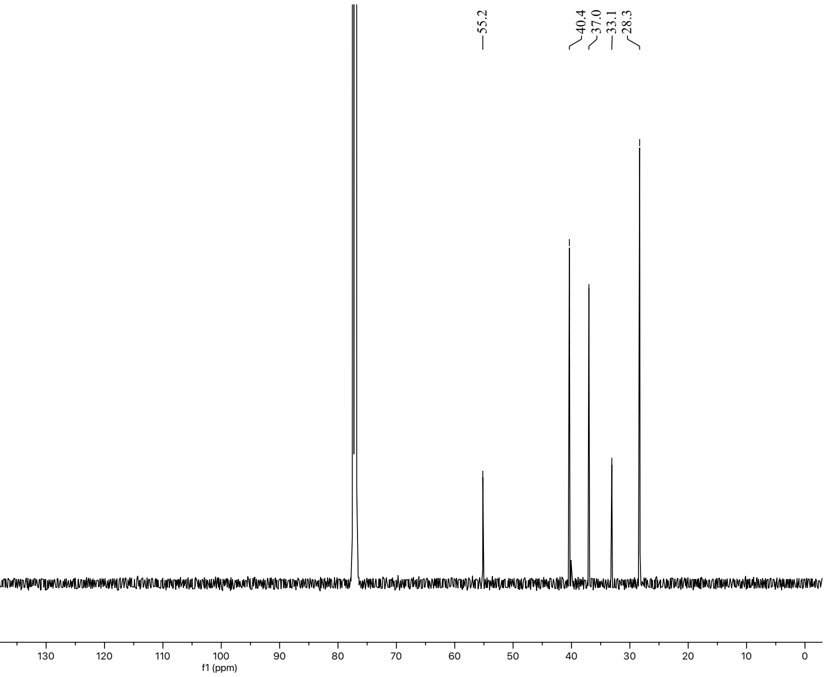

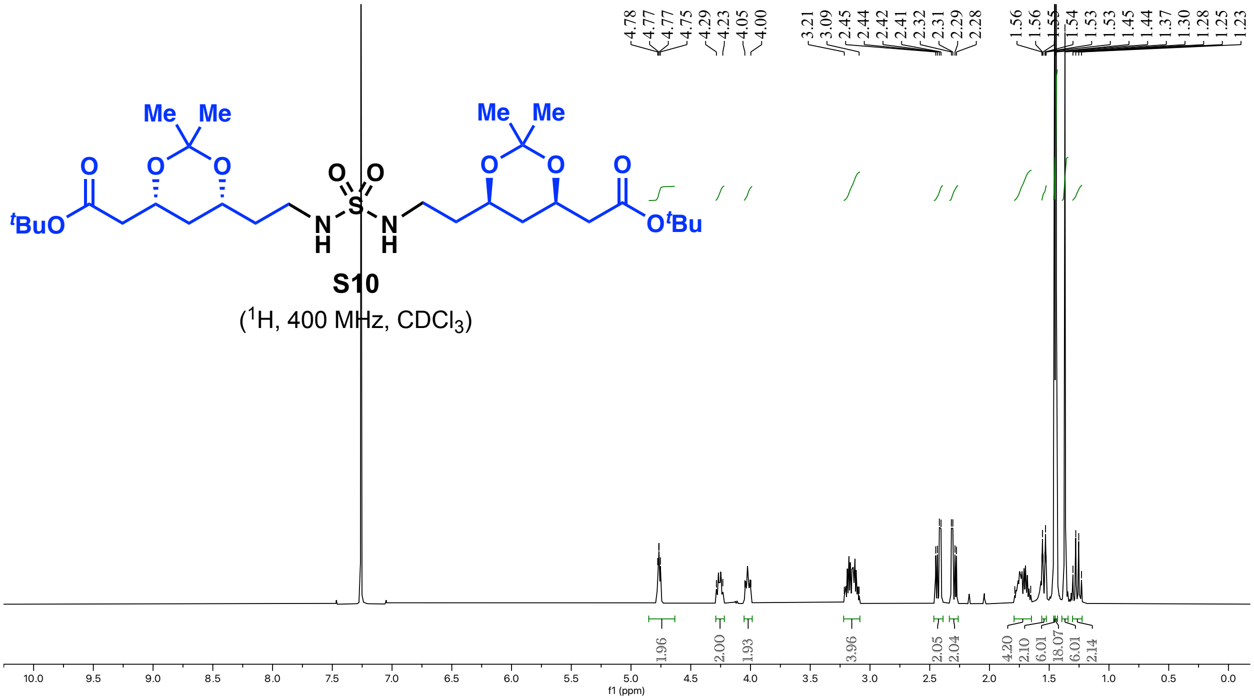

Me Me
O O O O
tBuO
O O
S
N N
H H
O tBu
S10
(1H, 400 MHz, CDCl3)
4.78 4.77 4.77 4.75 4.29 4.23 4.05 4.00
3.21 3.09 2.45 2.44 2.42 2.41 2.32 2.31 2.29 2.28
1.56 1.56 1.55 1.54 1.53 1.53 1.45 1.44 1.37 1.30 1.28 1.25 1.23
1.96 2.00 1.93 3.96 2.05 2.04 4.20 2.10 6.01 18.07 6.01 2.14
f1 (ppm)

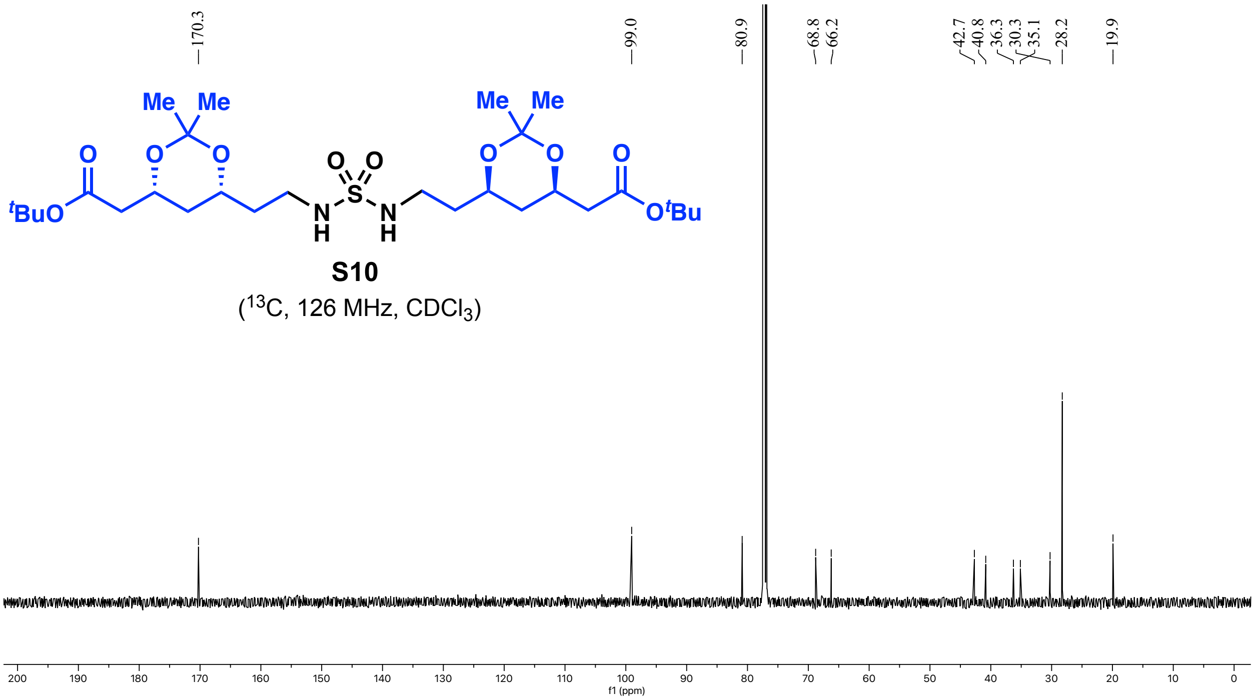

170.3 99.0 80.9 68.8 66.2 42.7 40.8 36.3 30.3 35.1 28.2 19.9
S10
(13C, 126 MHz, CDCl3)
f1 (ppm)

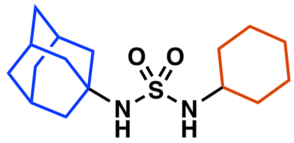

S11

(^{1}H, 400 MHz, $CDCl_3$)

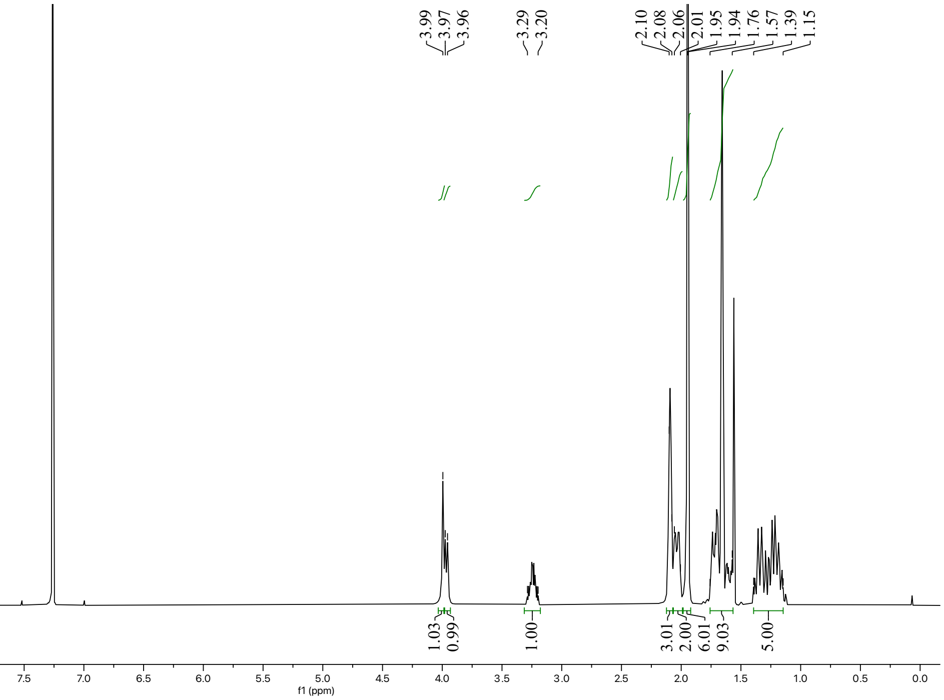

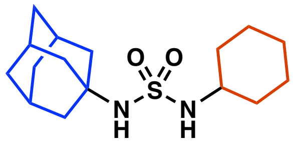

S11

(^{13}C, 126 MHz, $CDCl_3$)

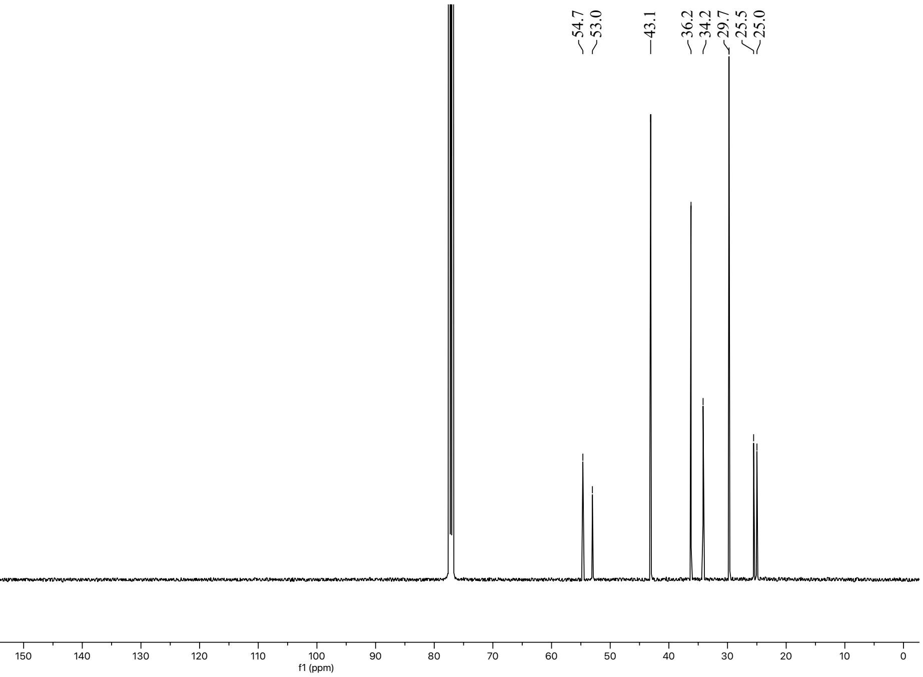

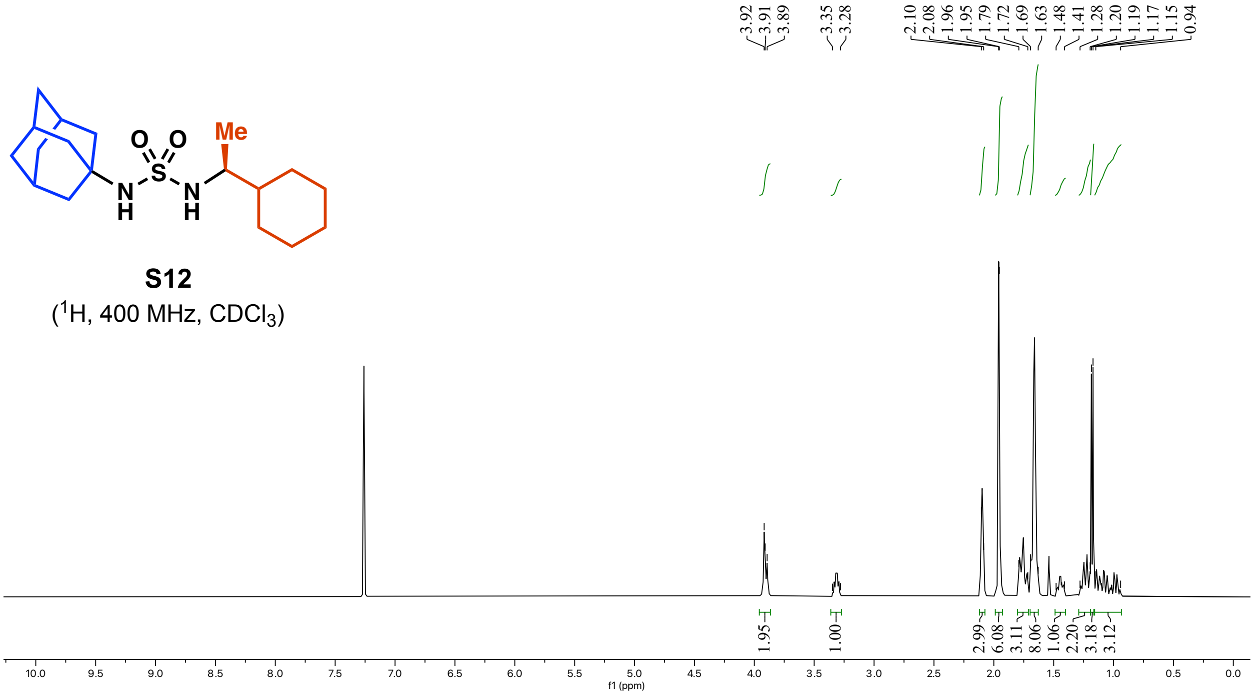

S12
(^{1}H, 400 MHz, $CDCl_3$)

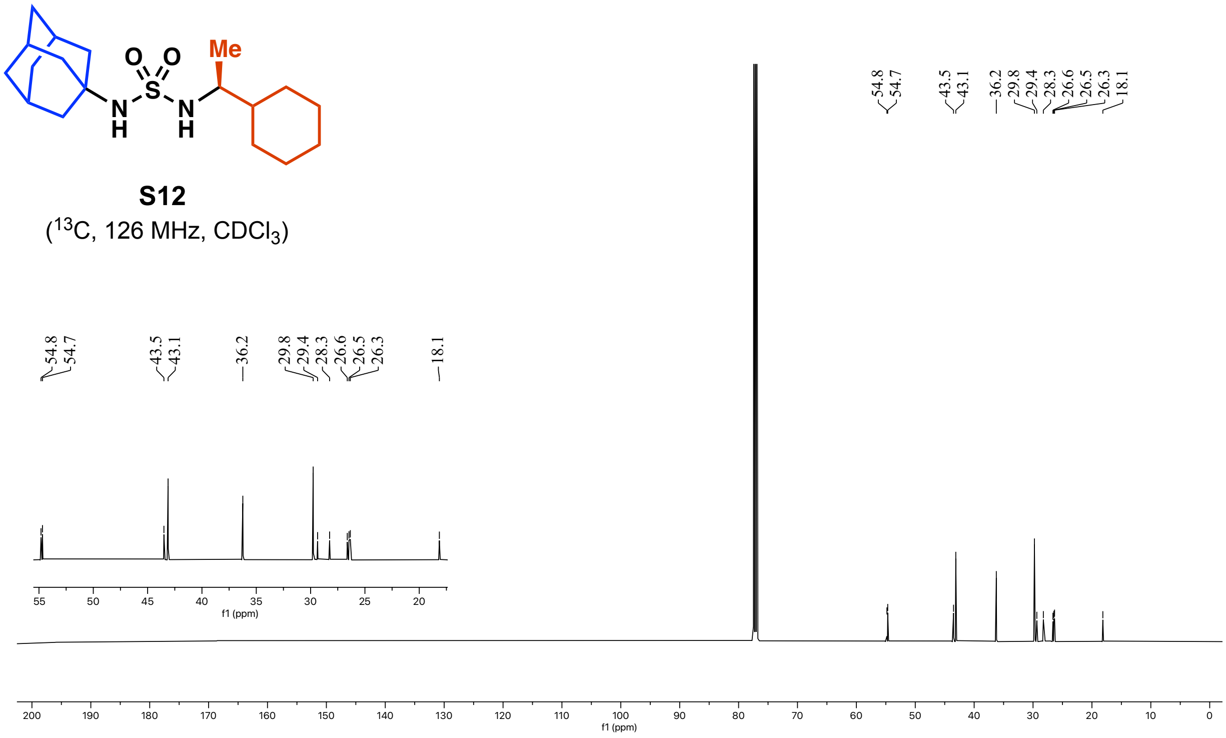

S12
(^{13}C, 126 MHz, $CDCl_3$)

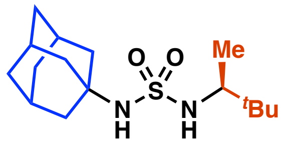

S13

(^{1}H, 400 MHz, $CDCl_3$)

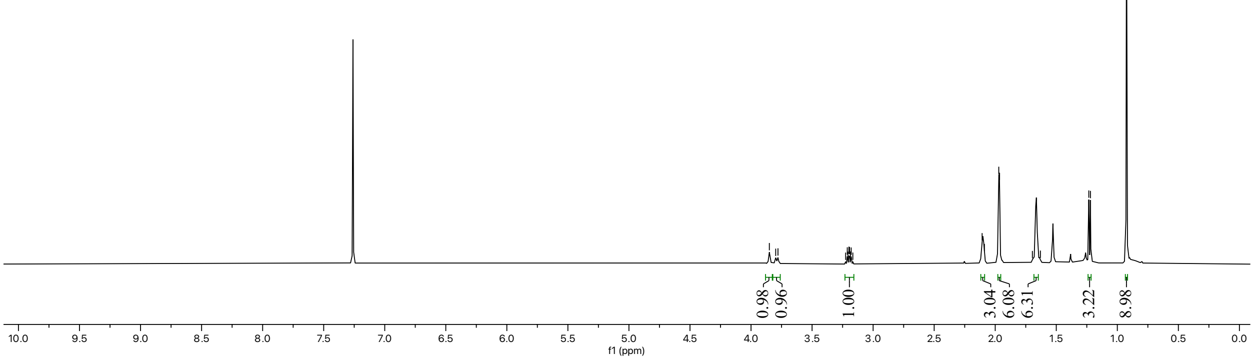

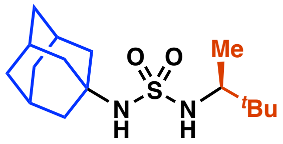

S13

(^{13}C, 126 MHz, $CDCl_3$)

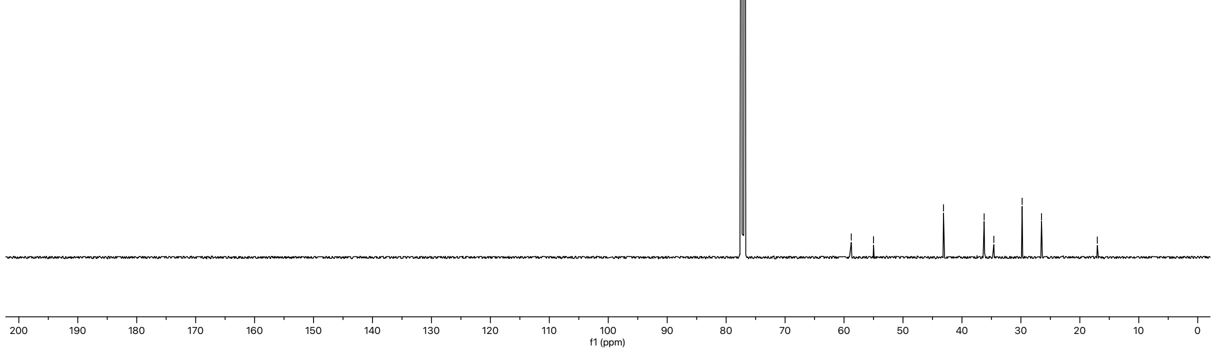

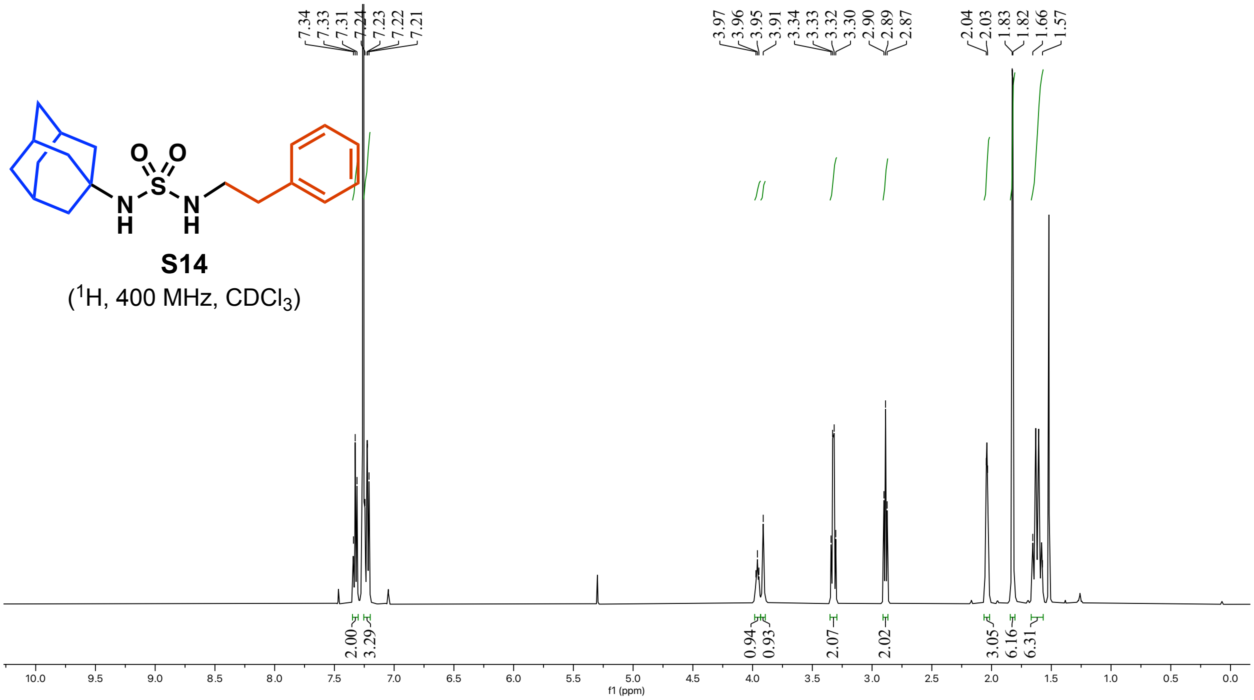

S14
(^{1}H, 400 MHz, CDCl$_3$)

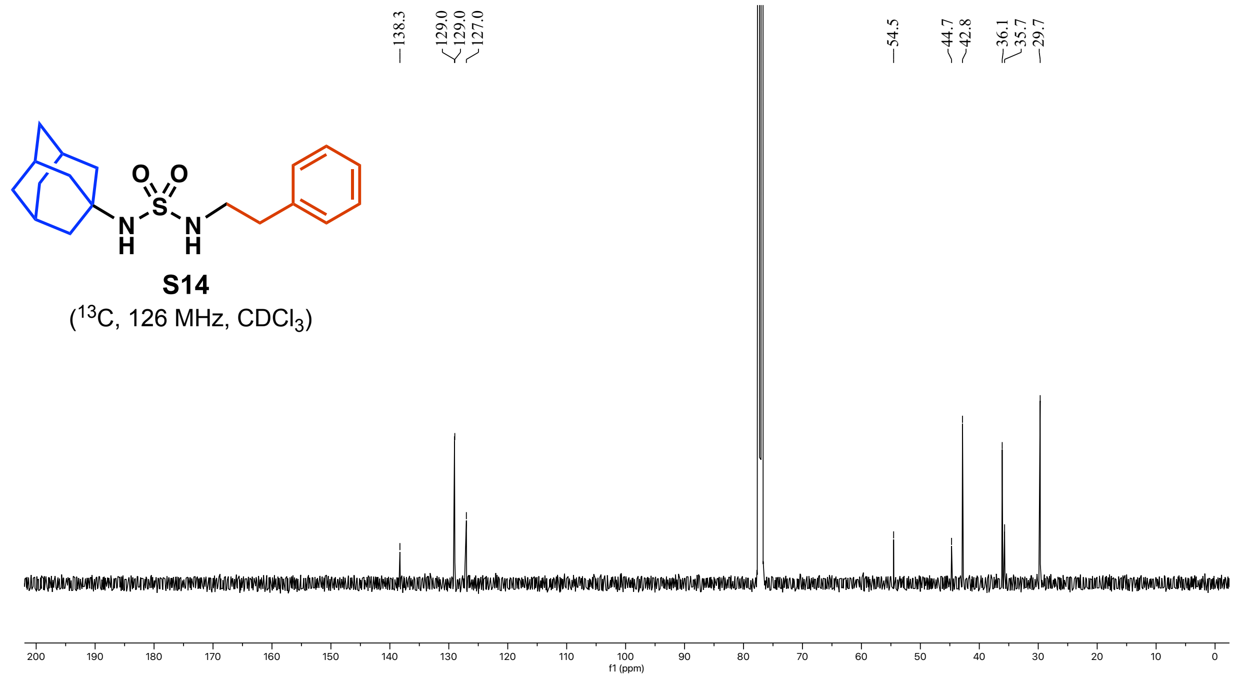

S14
(^{13}C, 126 MHz, CDCl$_3$)

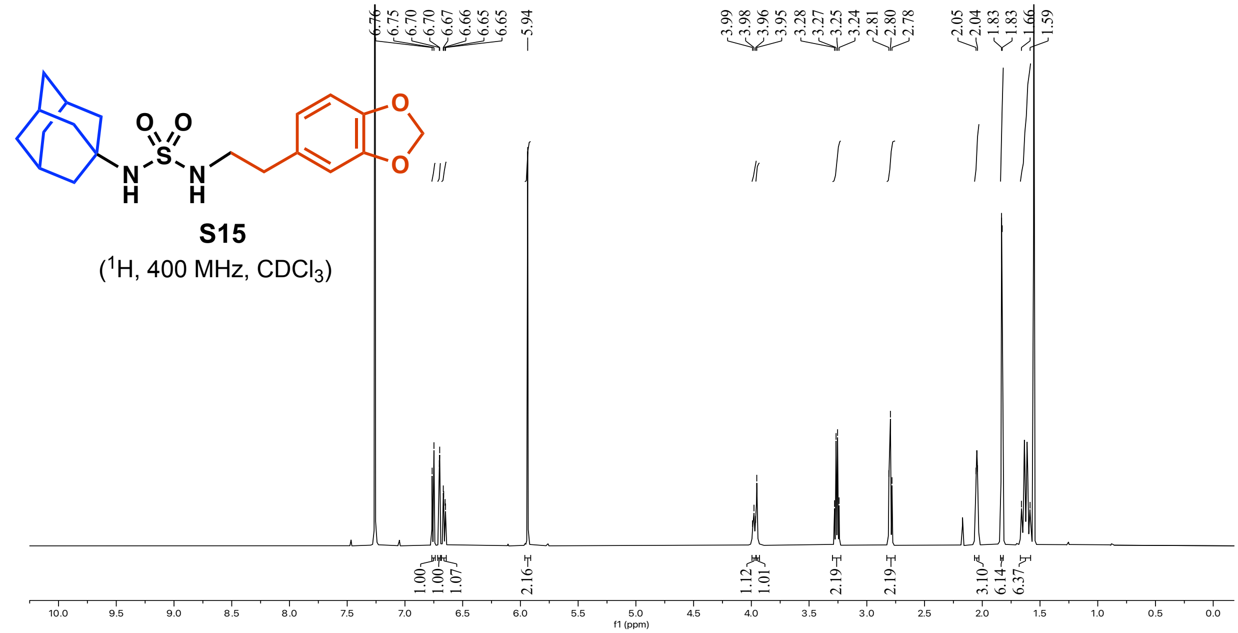
S15
(¹H, 400 MHz, CDCl₃)
6.76 6.75 6.70 6.70 6.67 6.66 6.65 6.65
5.94
3.99 3.98 3.96 3.95 3.28 3.27 3.25 3.24 2.81 2.80 2.78
2.05 2.04 1.83 1.83 1.66 1.59
1.00 1.00 1.07 2.16 1.12 1.01 2.19 2.19 3.10 6.14 6.37
f1 (ppm)

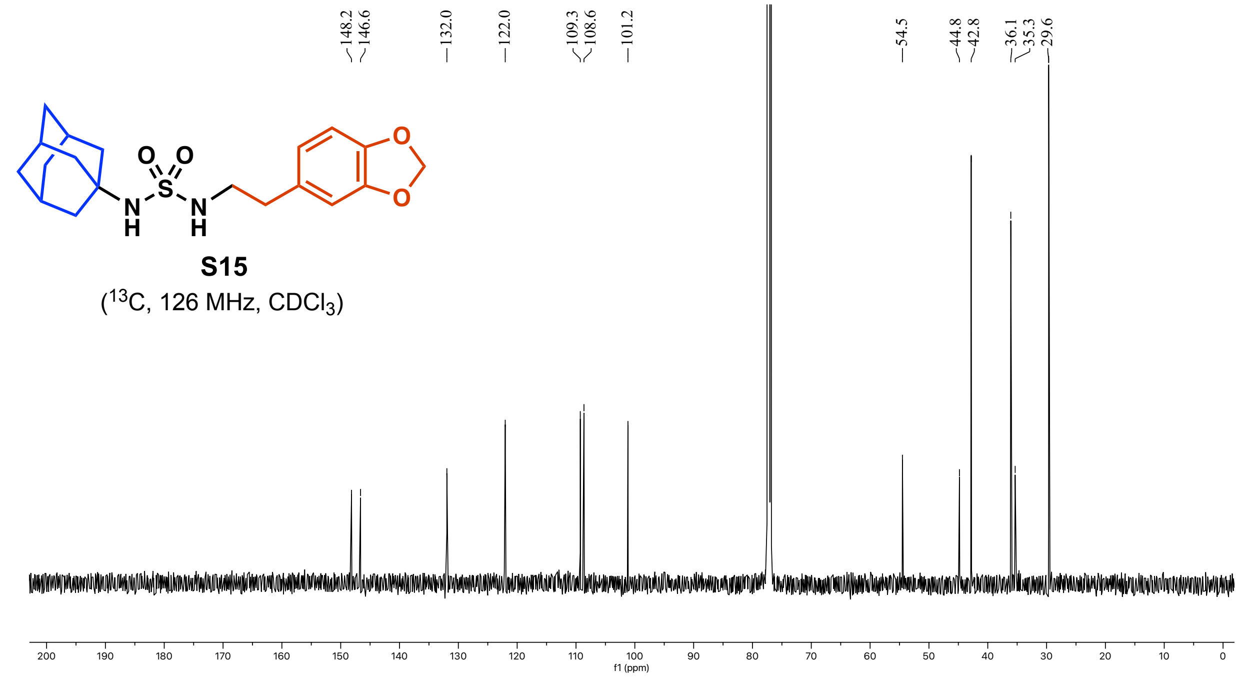
S15
(¹³C, 126 MHz, CDCl₃)
148.2 146.6 132.0 122.0 109.3 108.6 101.2 54.5 44.8 42.8 36.1 35.3 29.6
f1 (ppm)

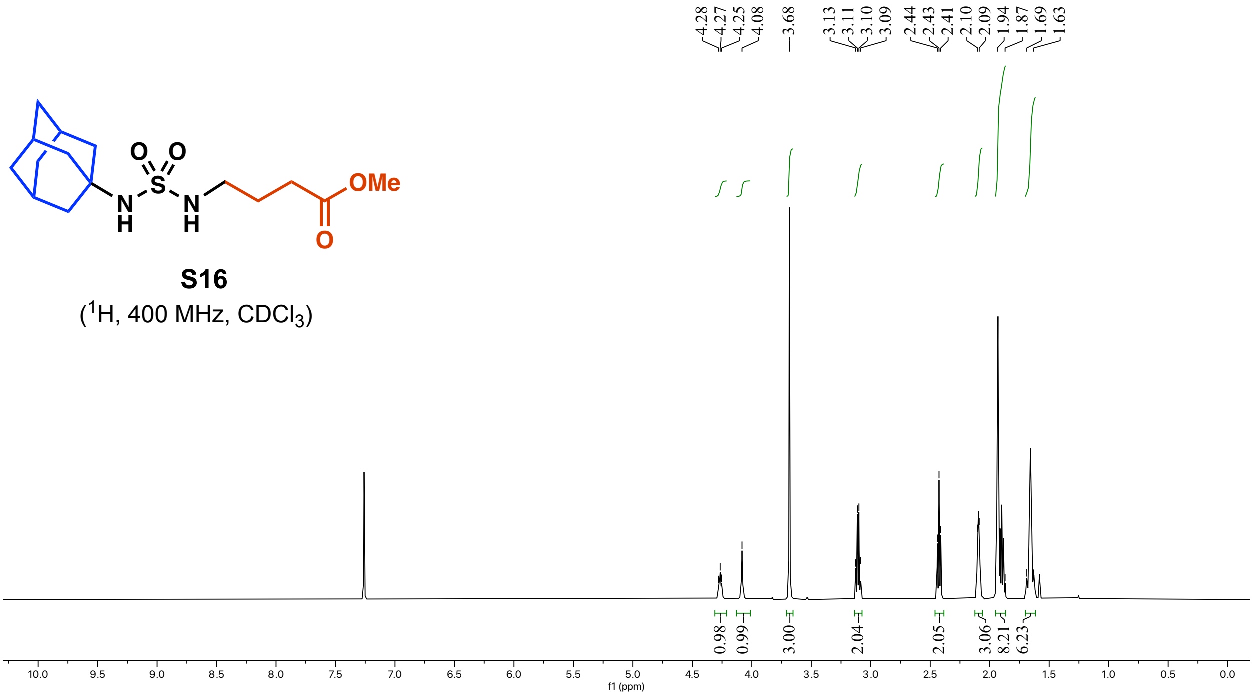

S16
(1H, 400 MHz, CDCl3)
f1 (ppm)

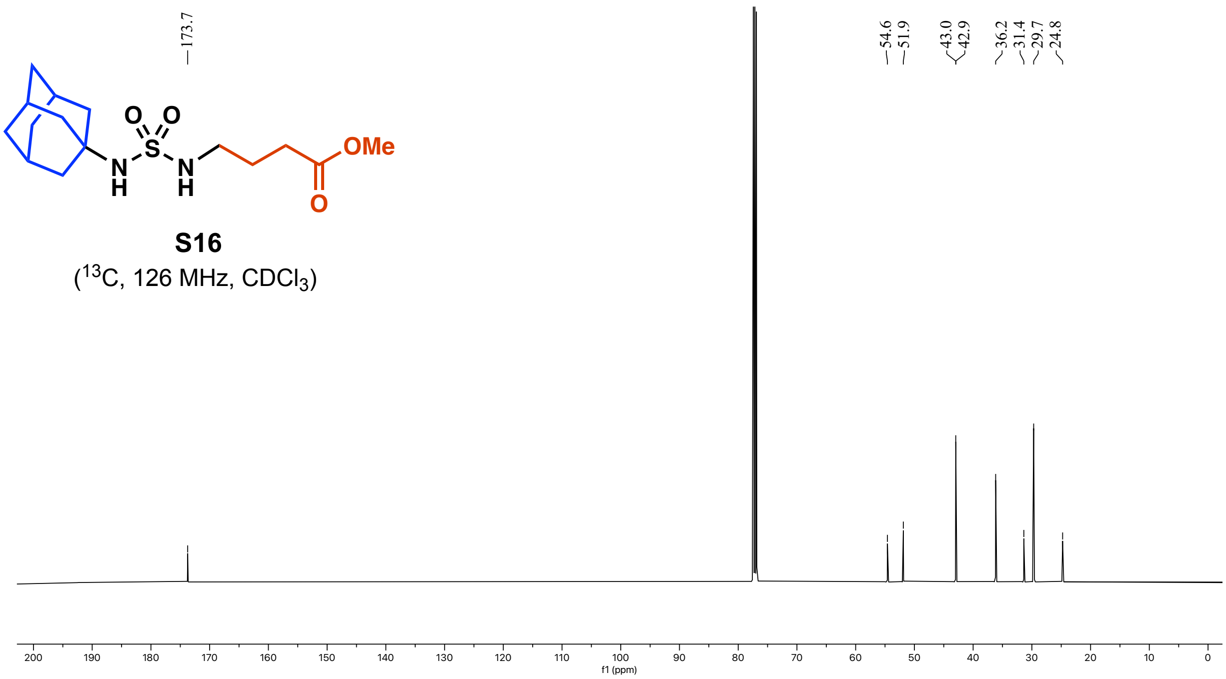

S16
(13C, 126 MHz, CDCl3)
f1 (ppm)

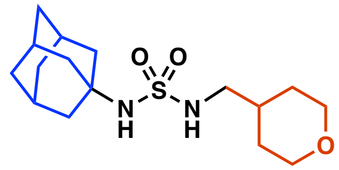

S17

(^{1}H, 400 MHz, CD_3CN)

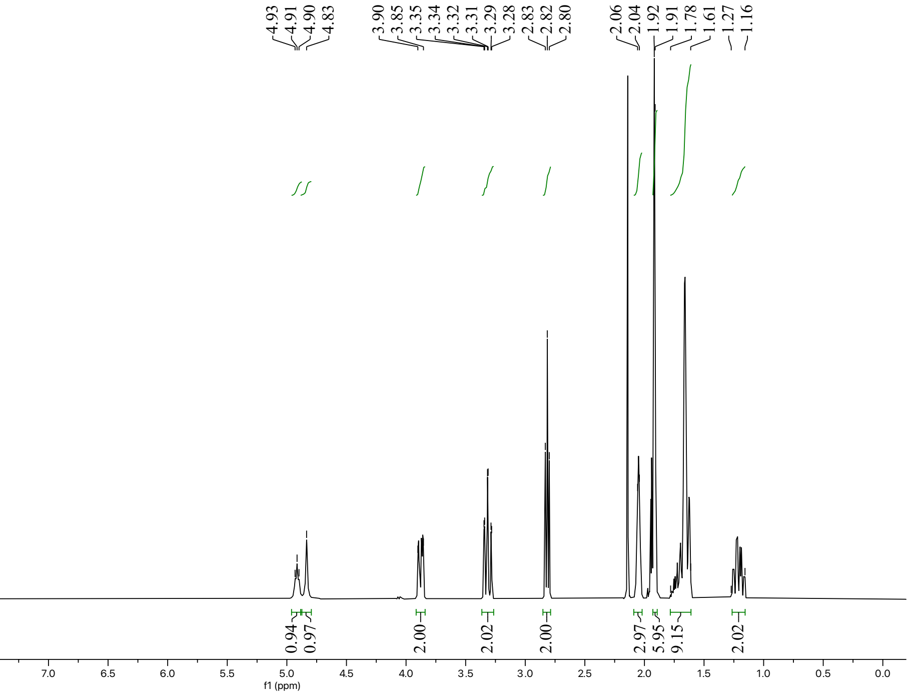

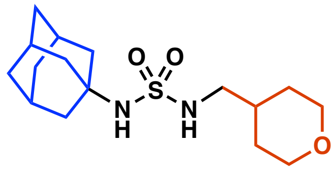

S17

(^{13}C, 126 MHz, CD_3CN)

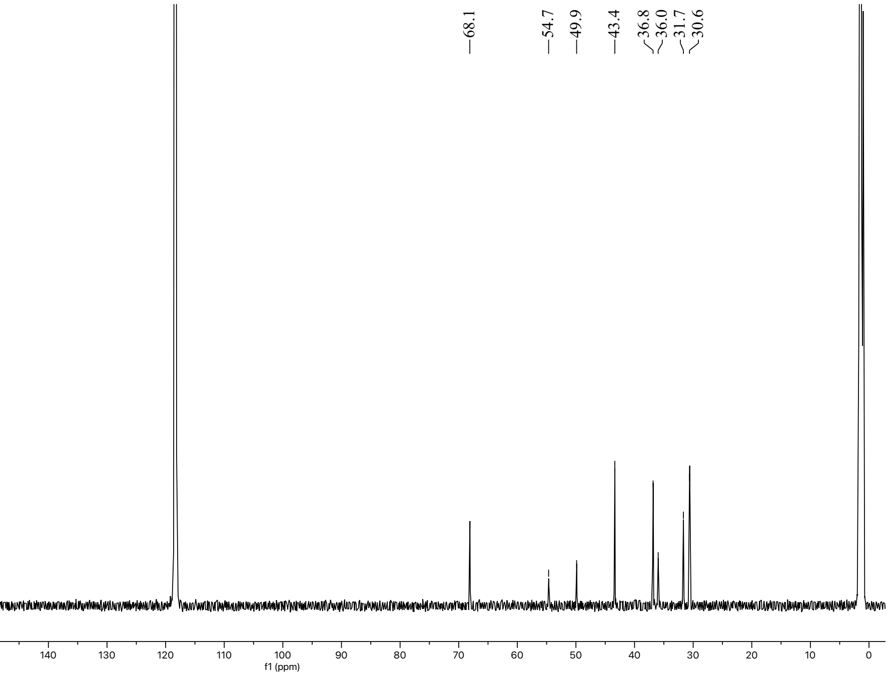

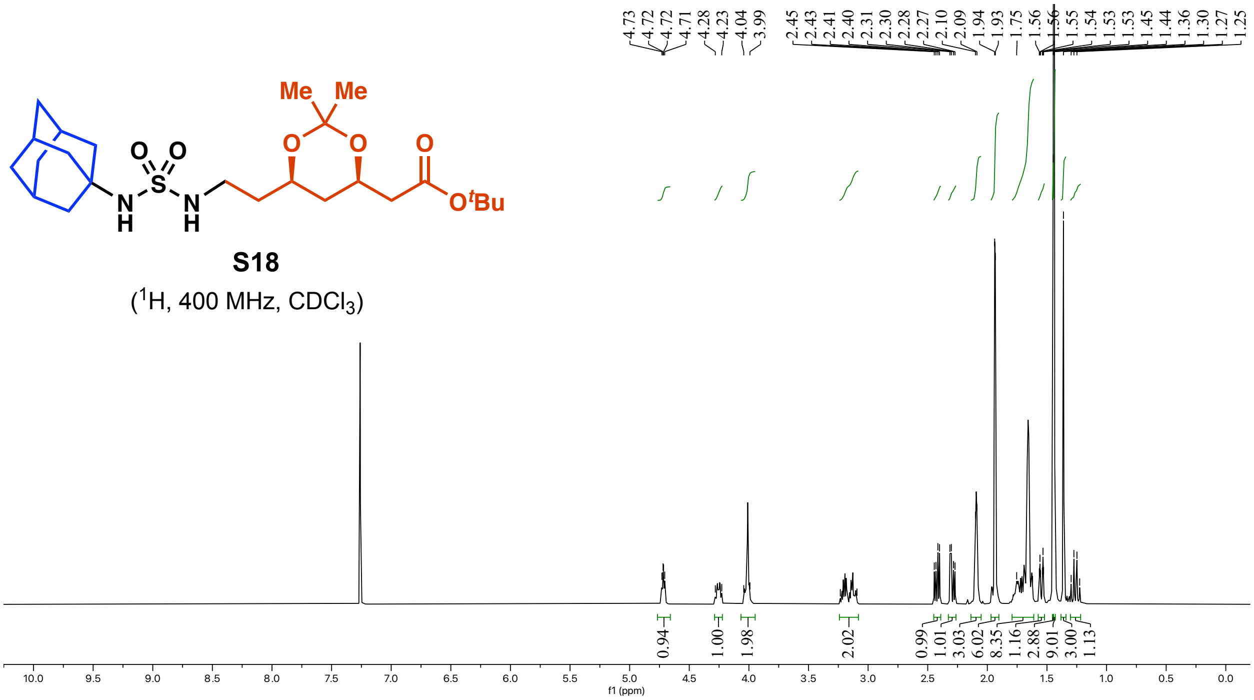

S18

(^{1}H, 400 MHz, $CDCl_3$)

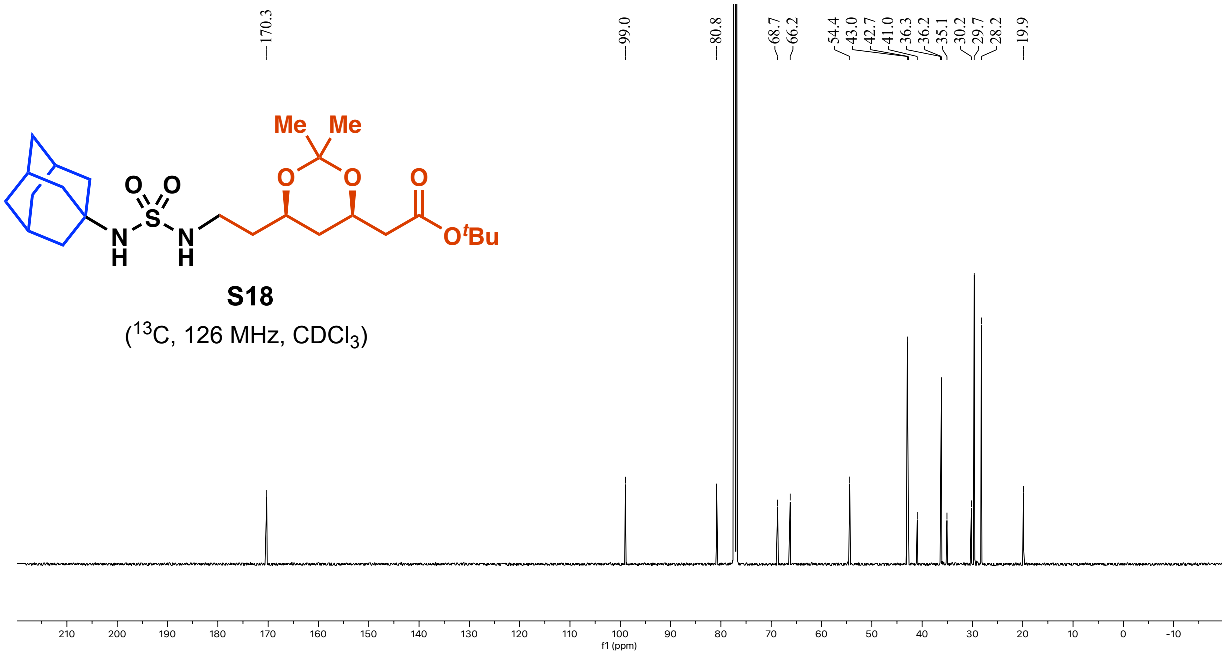

S18

(^{13}C, 126 MHz, $CDCl_3$)

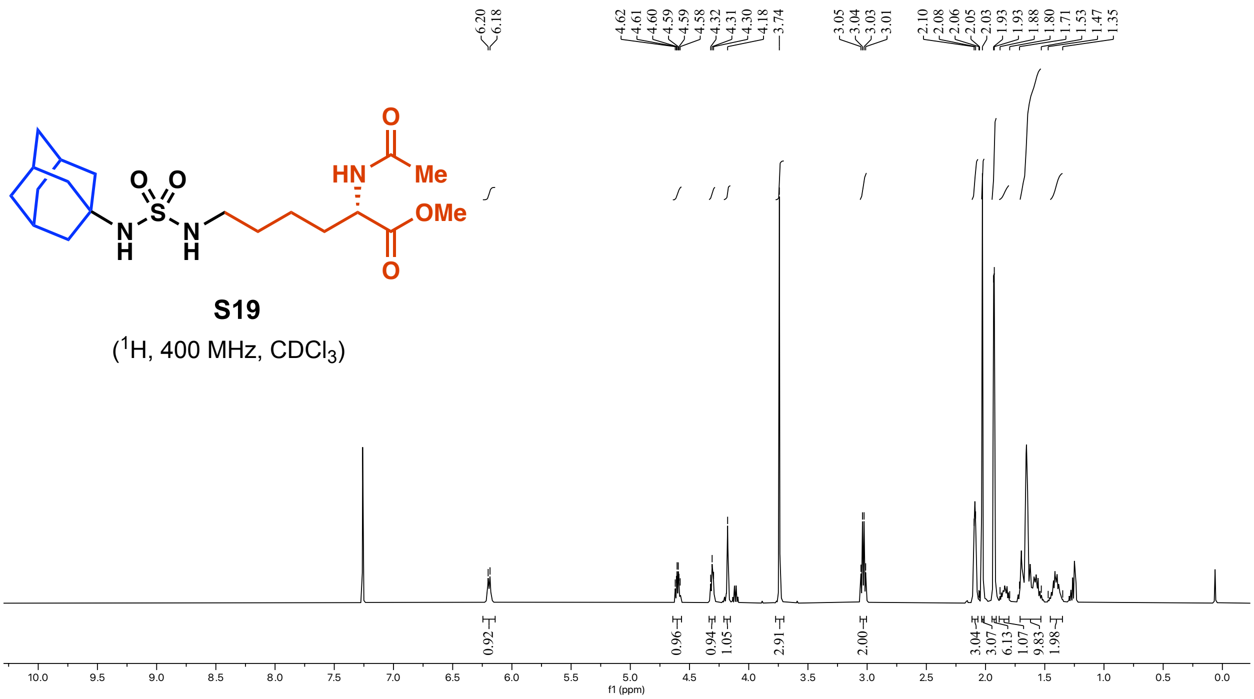

S19

(^{1}H, 400 MHz, $CDCl_3$)

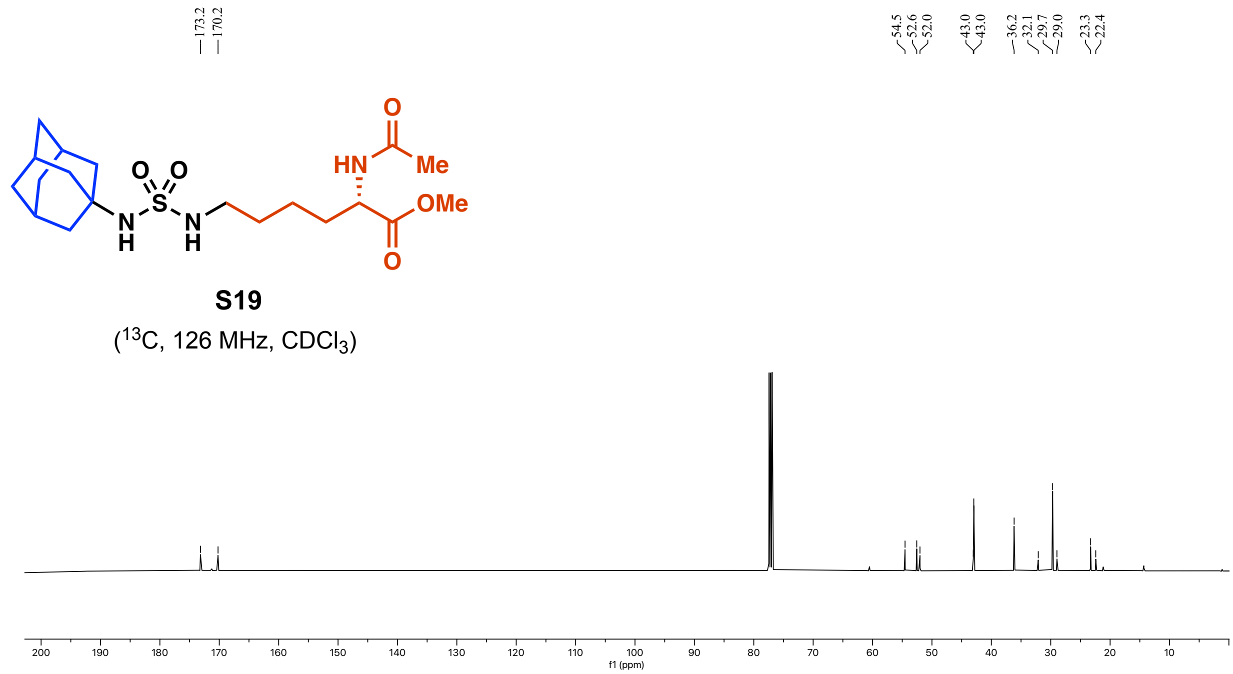

S19

(^{13}C, 126 MHz, $CDCl_3$)

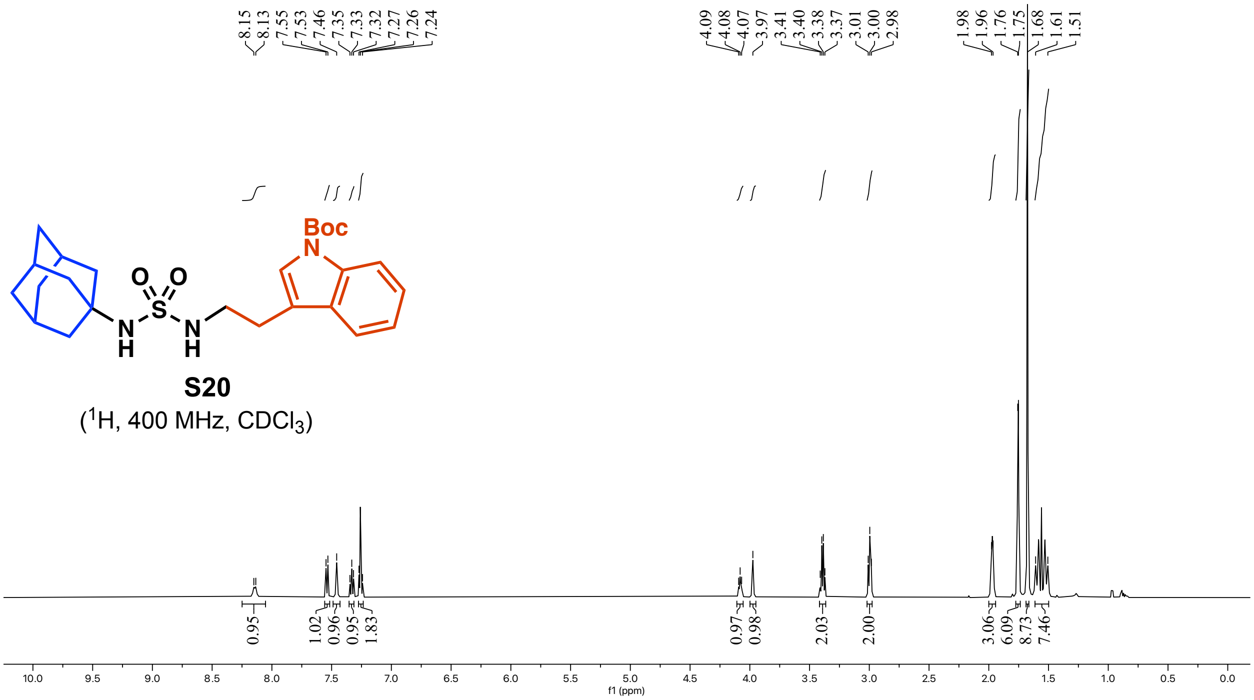
8.15
8.13
7.55
7.53
7.46
7.35
7.33
7.32
7.27
7.26
7.24
4.09
4.08
4.07
3.97
3.41
3.40
3.38
3.37
3.01
3.00
2.98
1.98
1.96
1.76
1.75
1.68
1.61
1.51
Boc
N
O O
S
N
H
N
H
S20
(^{1}H, 400 MHz, CDCl$_3$)
0.95
1.02
0.96
0.95
1.83
0.97
0.98
2.03
2.00
3.06
6.09
8.73
7.46
f1 (ppm)

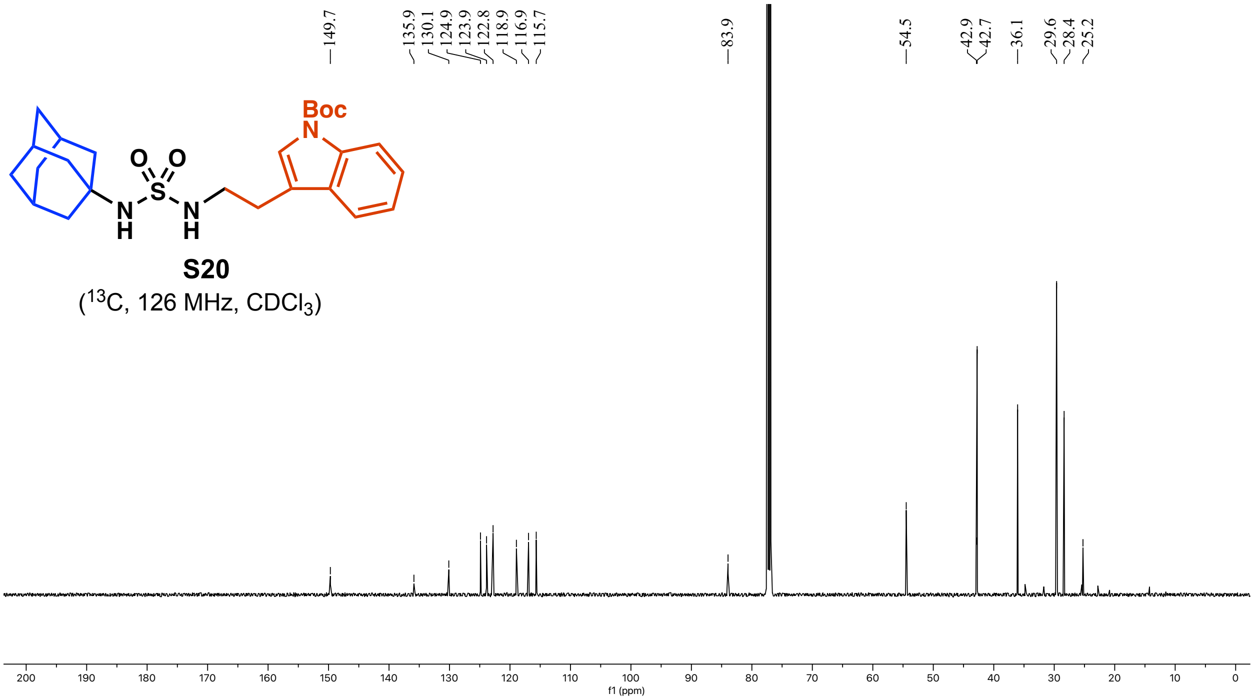
149.7
135.9
130.1
124.9
123.9
122.8
118.9
116.9
115.7
83.9
54.5
42.9
42.7
36.1
29.6
28.4
25.2
Boc
N
O O
S
N
H
N
H
S20
(^{13}C, 126 MHz, CDCl$_3$)
f1 (ppm)

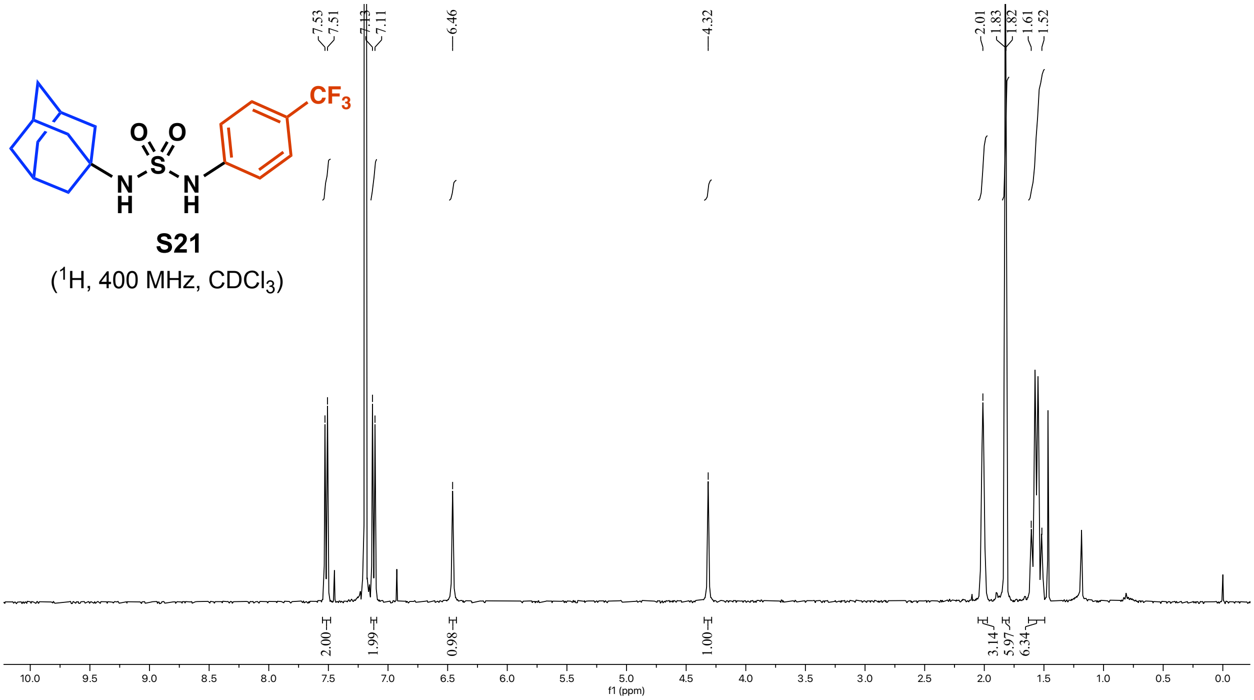
7.53
7.51
7.13
7.11
6.46
4.32
2.01
1.83
1.82
1.61
1.52
CF3
O O
S
N N
H H
S21
(1H, 400 MHz, CDCl3)
2.00
1.99
0.98
1.00
3.14
5.97
6.34
f1 (ppm)

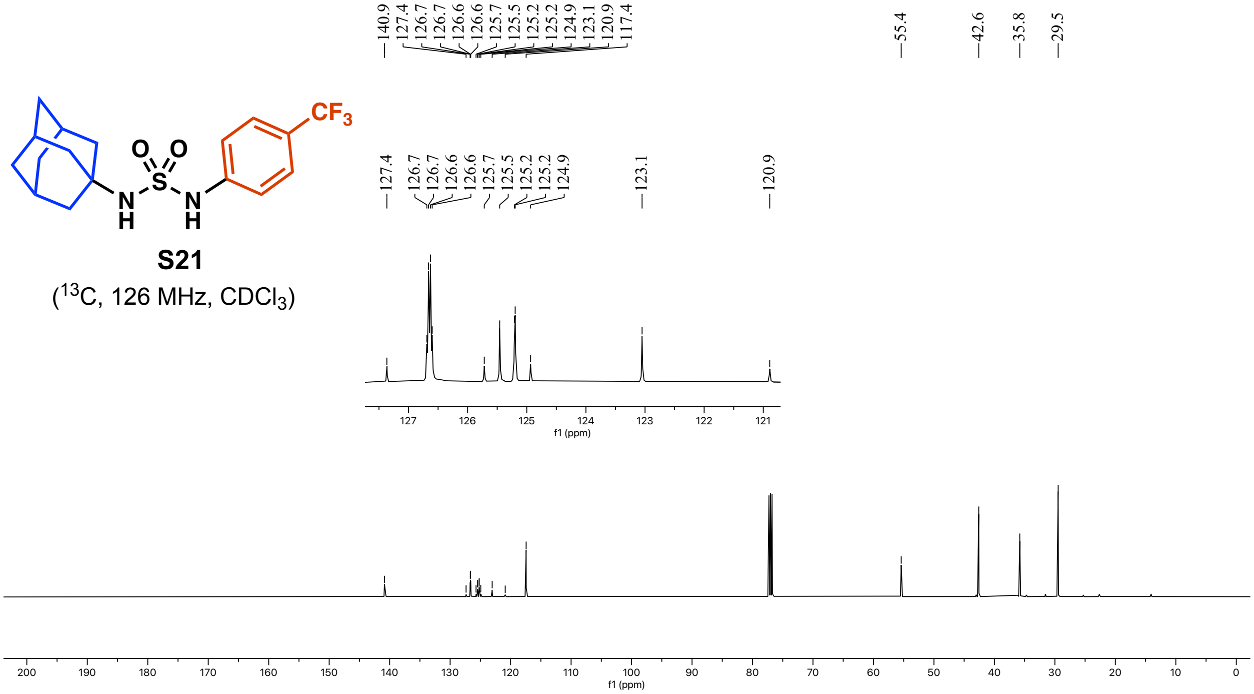
140.9
127.4
126.7
126.7
126.6
126.6
125.7
125.5
125.2
125.2
124.9
123.1
120.9
117.4
55.4
42.6
35.8
29.5
CF3
O O
S
N N
H H
S21
(13C, 126 MHz, CDCl3)
f1 (ppm)

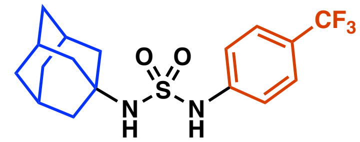

S21

(^{19}F, 470 MHz, $CDCl_3$)

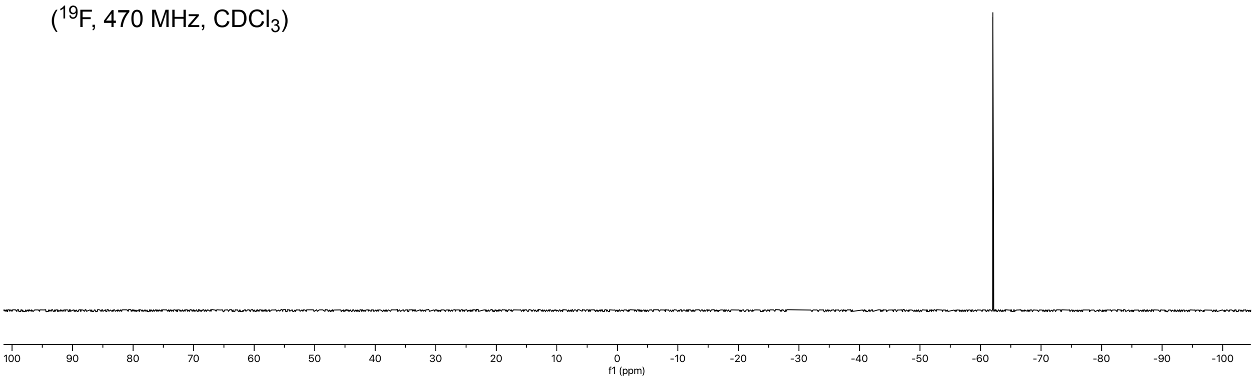

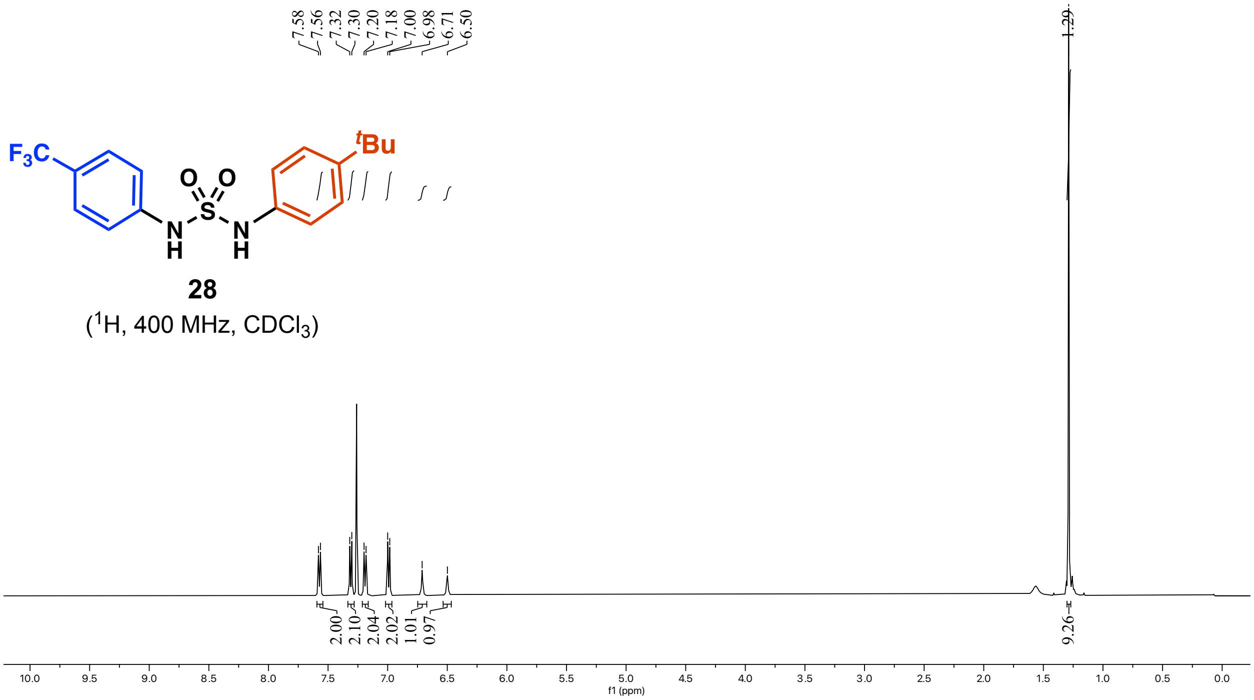

28

(^{1}H, 400 MHz, $CDCl_3$)

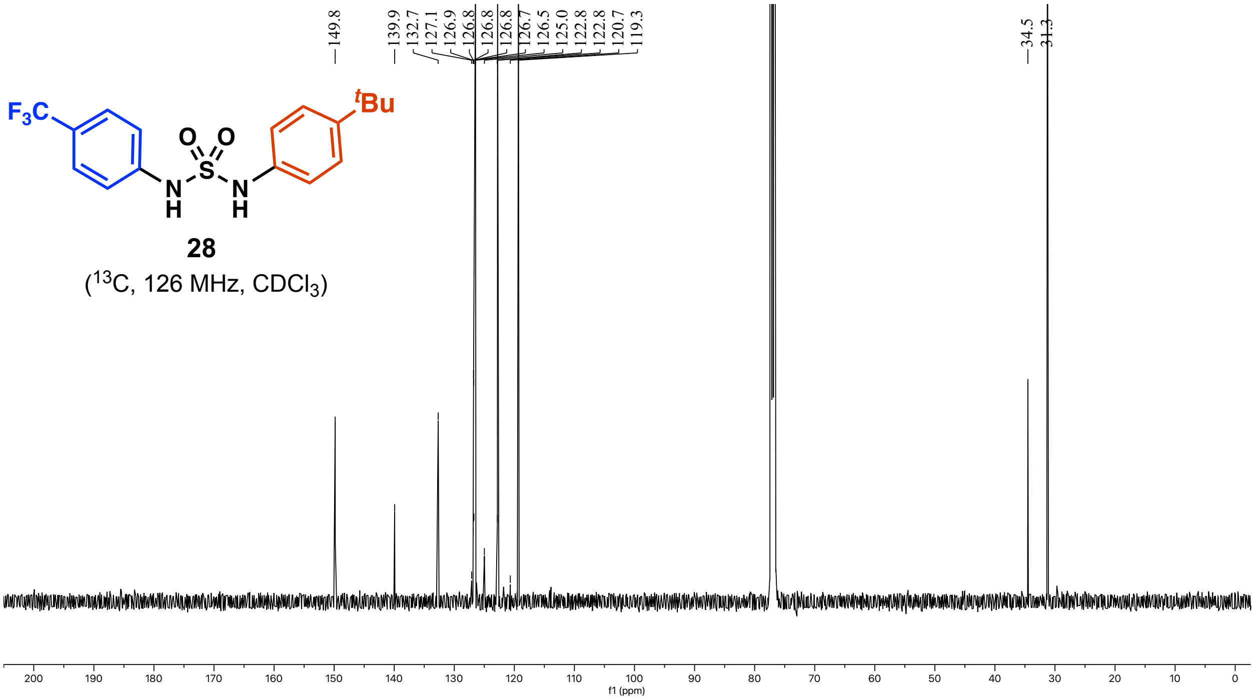

28

(^{13}C, 126 MHz, $CDCl_3$)

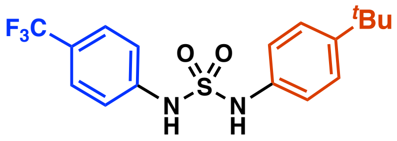

28

(^{19}F, 470 MHz, $CDCl_3$)

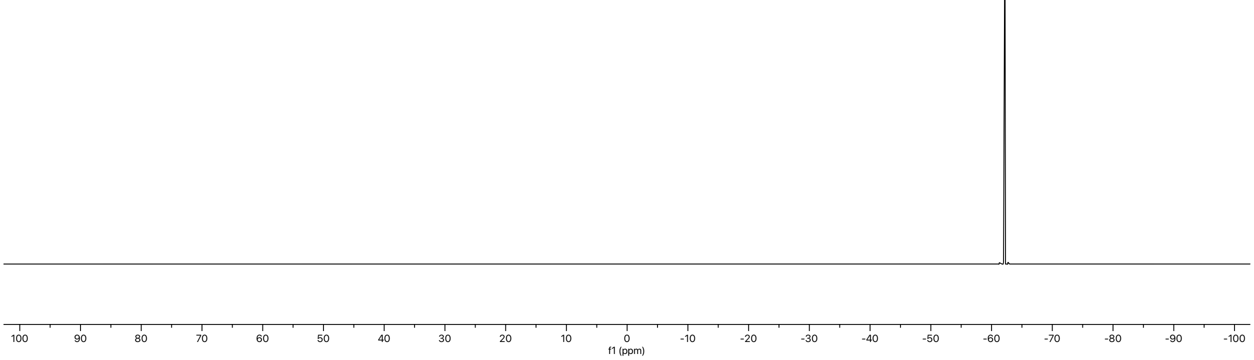

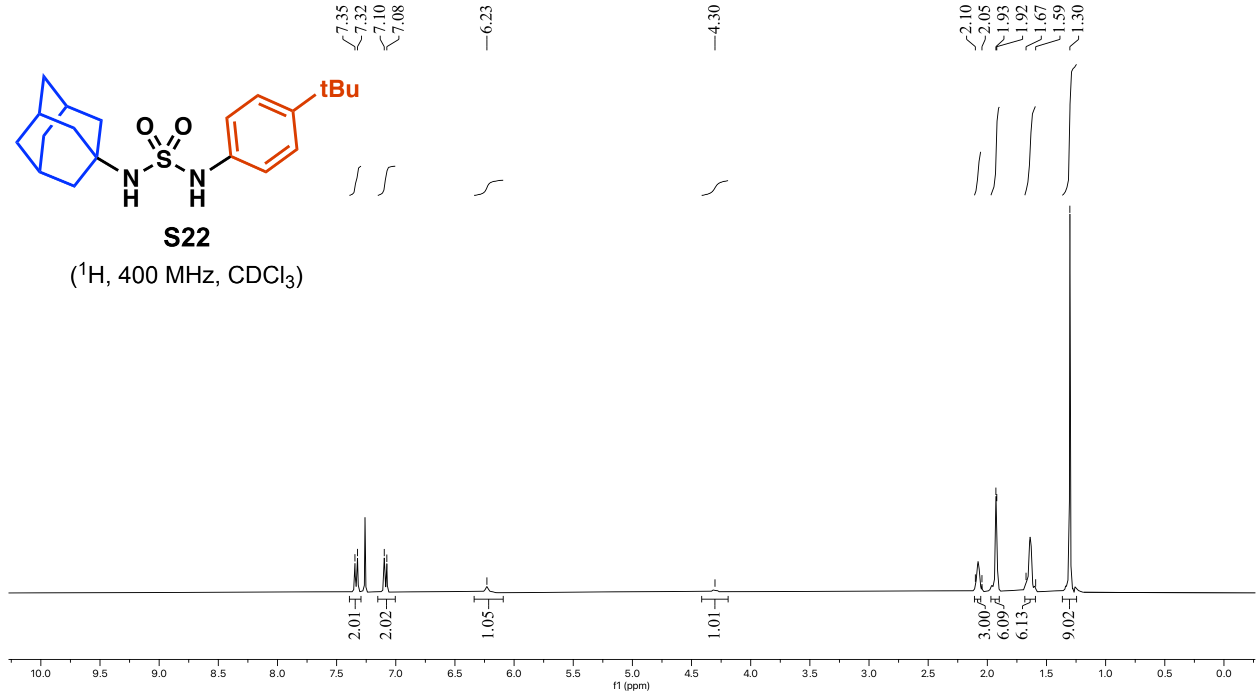
7.35
7.32
7.10
7.08
6.23
4.30
2.10
2.05
1.93
1.92
1.67
1.59
1.30
tBu
O O
S
N N
H H
S22
(^{1}H, 400 MHz, $CDCl_3$)
2.01
2.02
1.05
1.01
3.00
6.09
6.13
9.02
10.0 9.5 9.0 8.5 8.0 7.5 7.0 6.5 6.0 5.5 5.0 4.5 4.0 3.5 3.0 2.5 2.0 1.5 1.0 0.5 0.0
f1 (ppm)

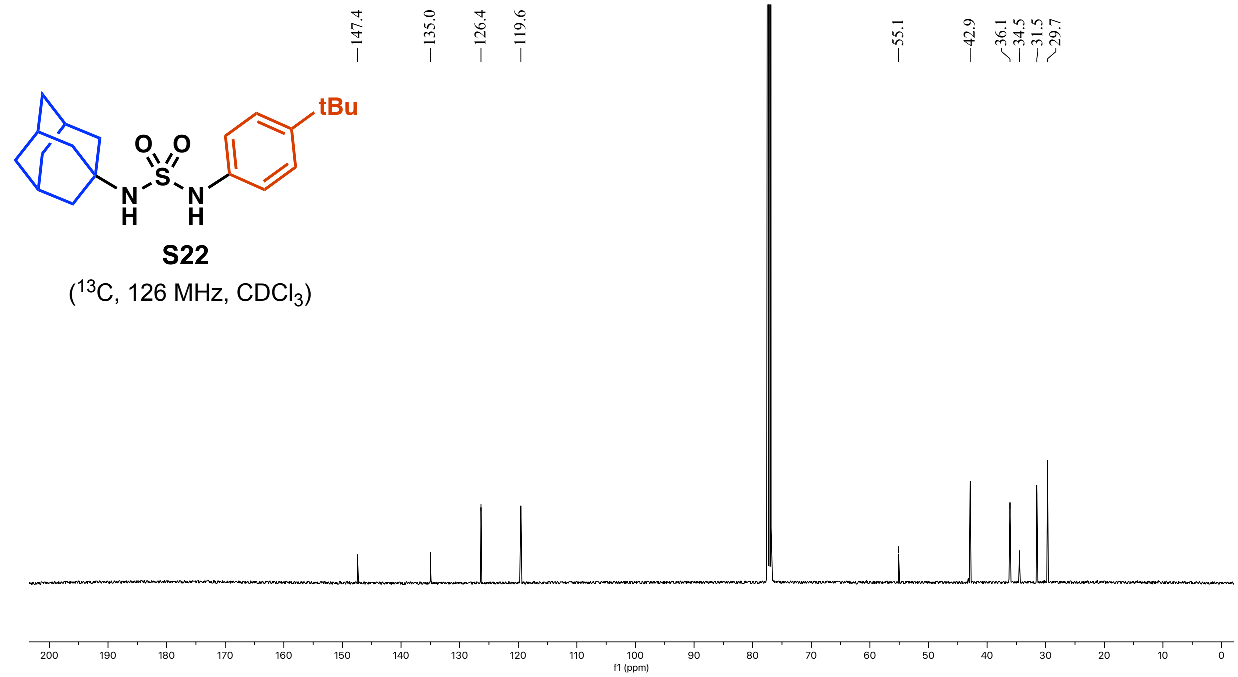
147.4
135.0
126.4
119.6
55.1
42.9
36.1
34.5
31.5
29.7
tBu
O O
S
N N
H H
S22
(^{13}C, 126 MHz, $CDCl_3$)
200 190 180 170 160 150 140 130 120 110 100 90 80 70 60 50 40 30 20 10 0
f1 (ppm)

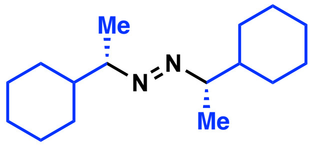

5

(^{1}H, 400 MHz, $CDCl_3$)

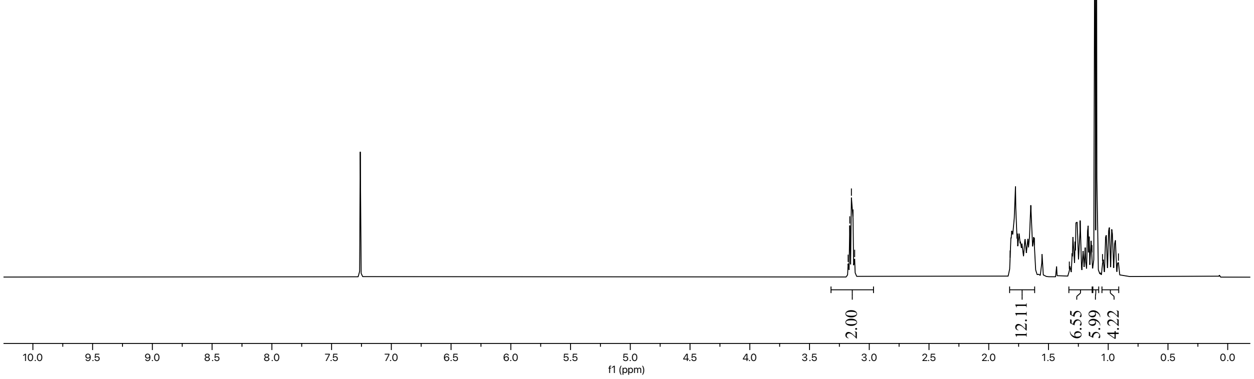

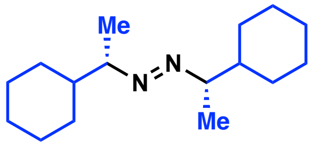

5

(^{1}H, 400 MHz, $CDCl_3$)

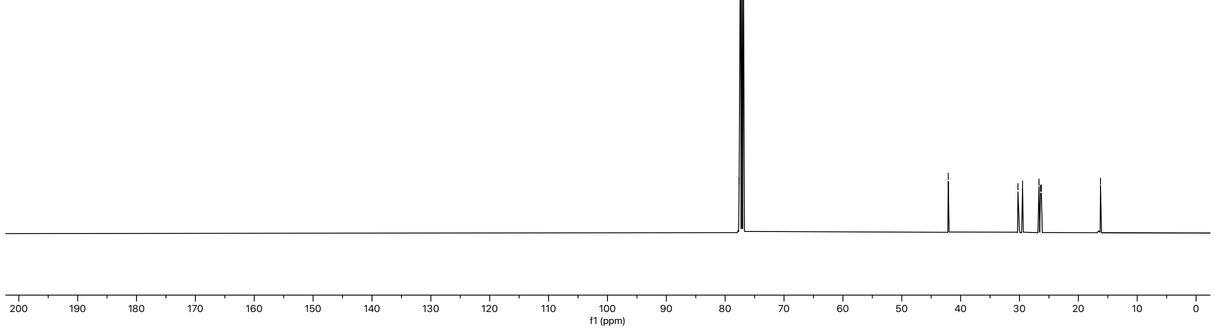

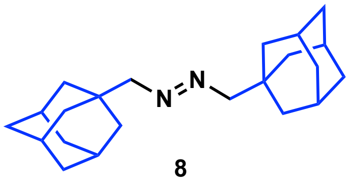

8

(^{1}H, 400 MHz, $CDCl_3$)

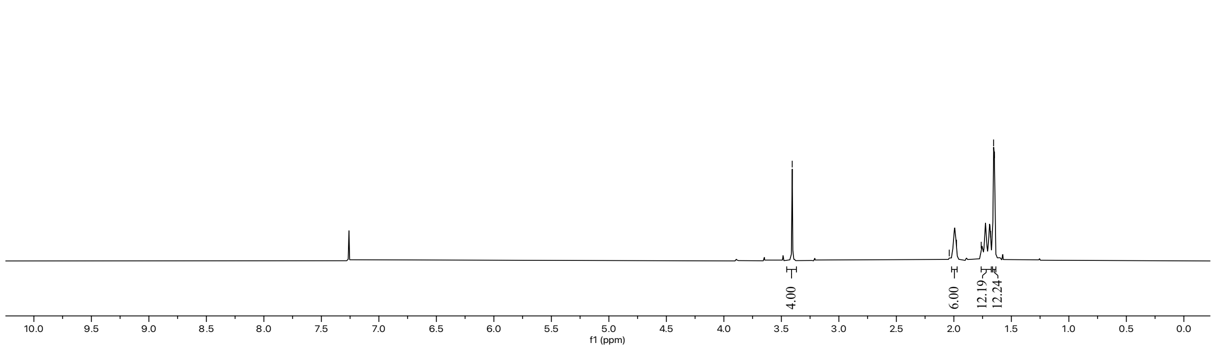

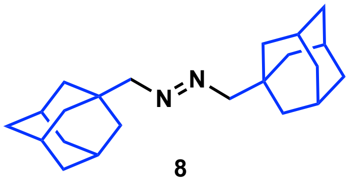

8

(^{13}C, 126 MHz, $CDCl_3$)

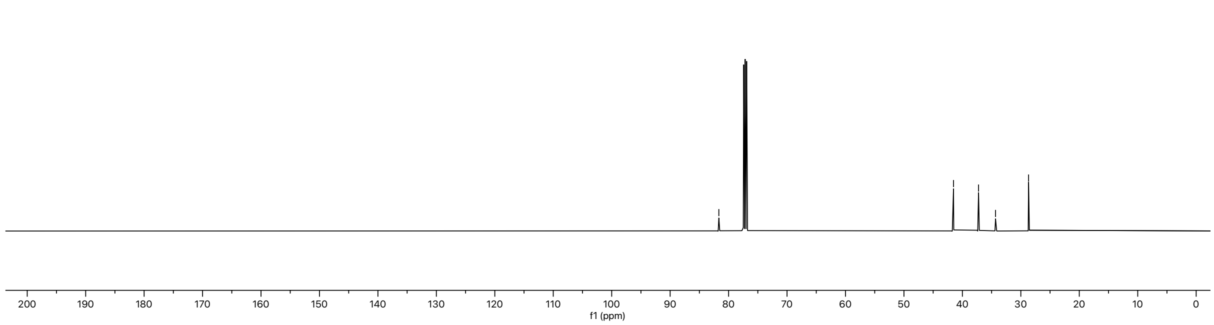

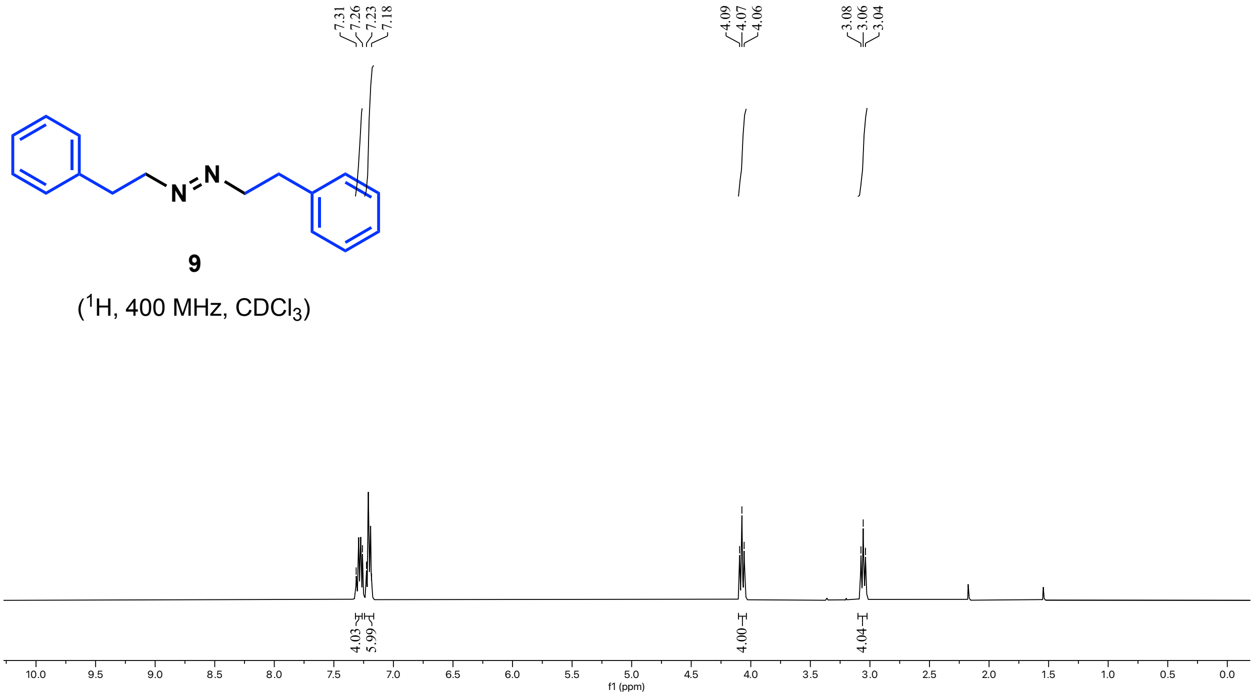
7.31
7.26
7.23
7.18
4.09
4.07
4.06
3.08
3.06
3.04
N
N
9
(¹H, 400 MHz, CDCl₃)
4.03
5.99
4.00
4.04
10.0
9.5
9.0
8.5
8.0
7.5
7.0
6.5
6.0
5.5
5.0
4.5
4.0
3.5
3.0
2.5
2.0
1.5
1.0
0.5
0.0
f1 (ppm)

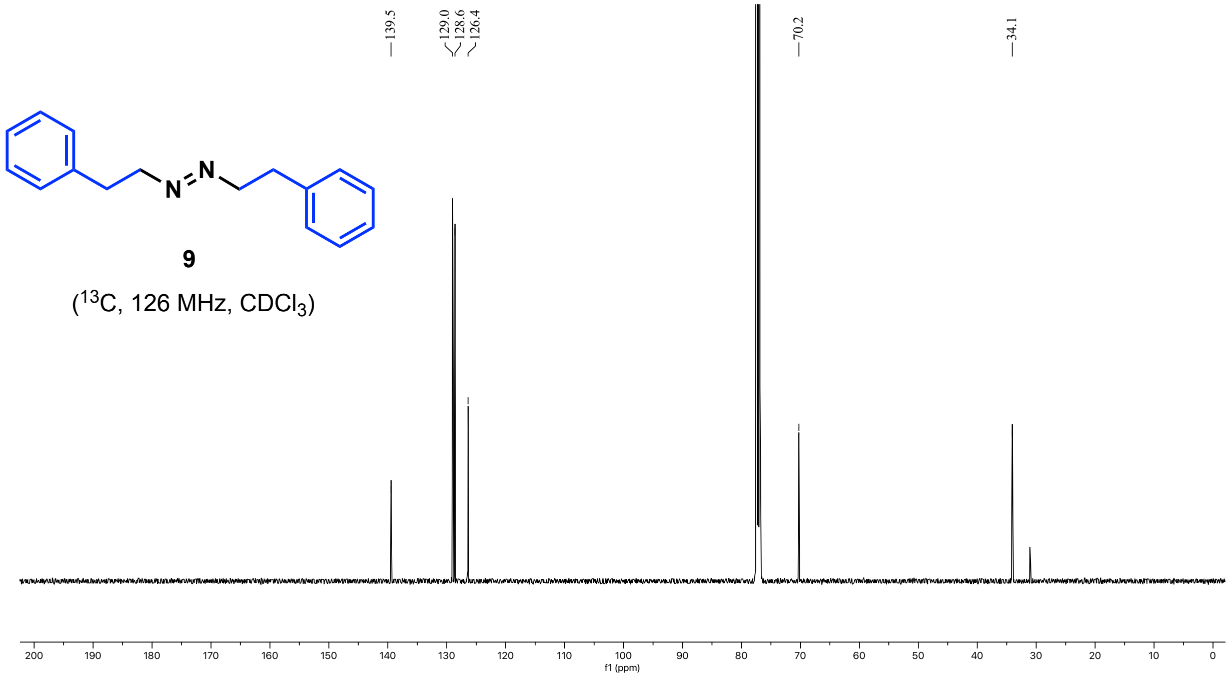
139.5
129.0
128.6
126.4
70.2
34.1
N
N
9
(¹³C, 126 MHz, CDCl₃)
200
190
180
170
160
150
140
130
120
110
100
90
80
70
60
50
40
30
20
10
0
f1 (ppm)

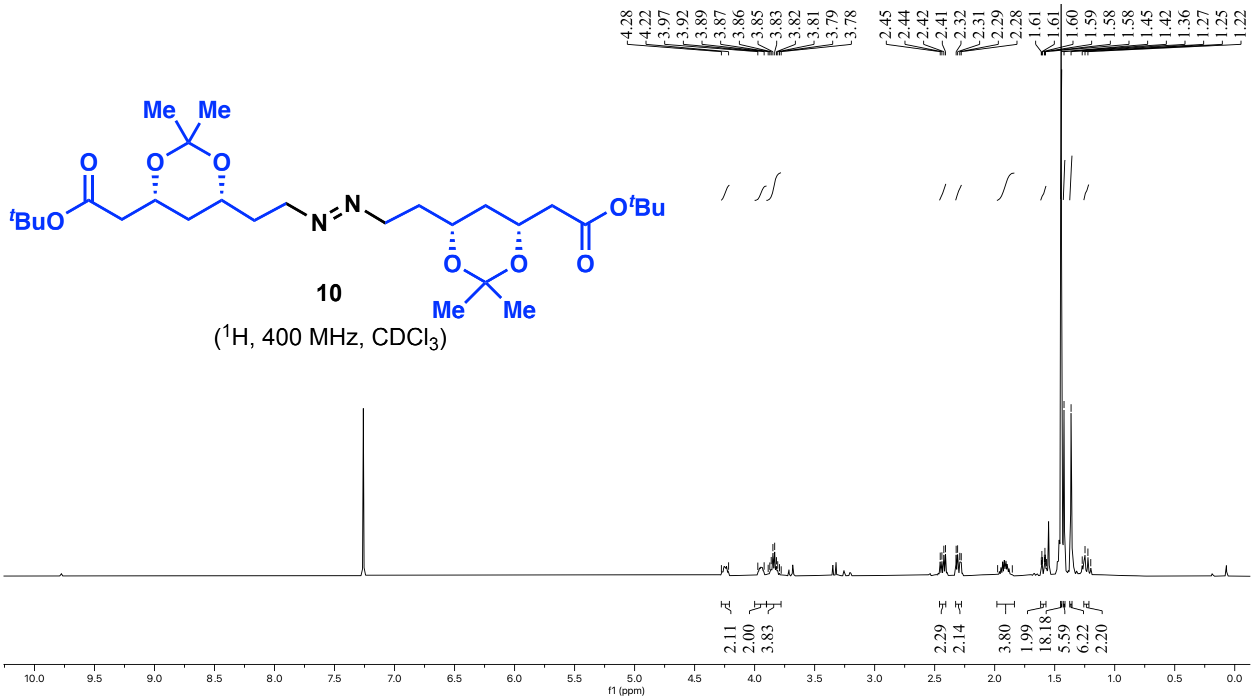

10

(^{1}H, 400 MHz, $CDCl_3$)

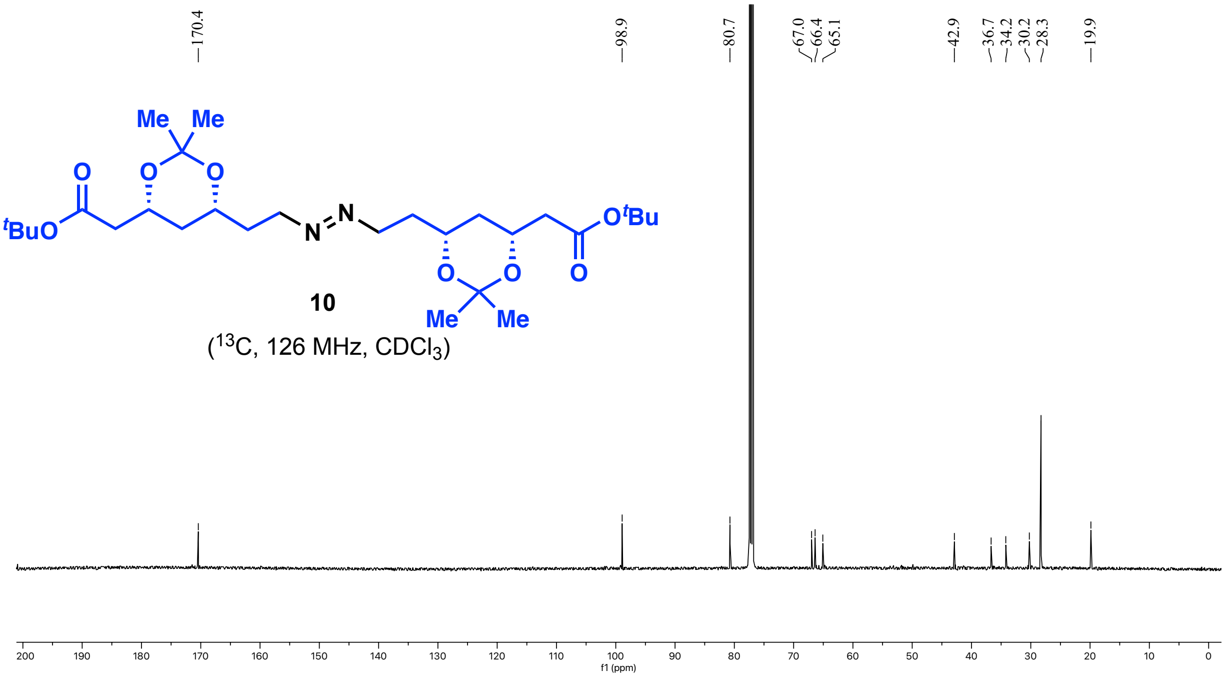

10

(^{13}C, 126 MHz, $CDCl_3$)

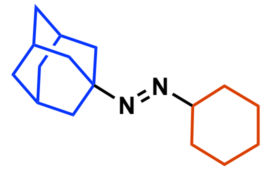

11

(^{1}H, 400 MHz, $CDCl_3$)

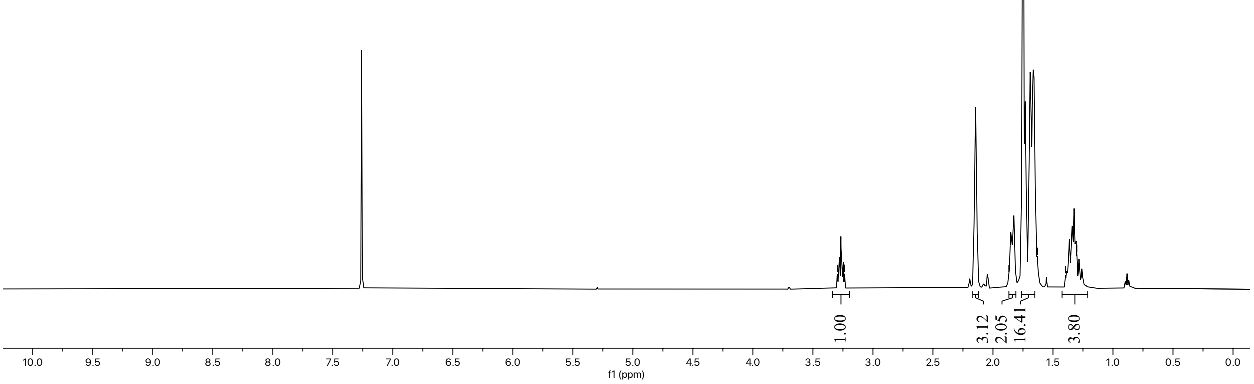

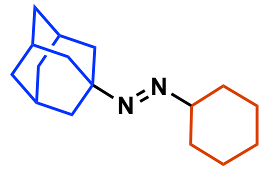

11

(^{13}C, 126 MHz, $CDCl_3$)

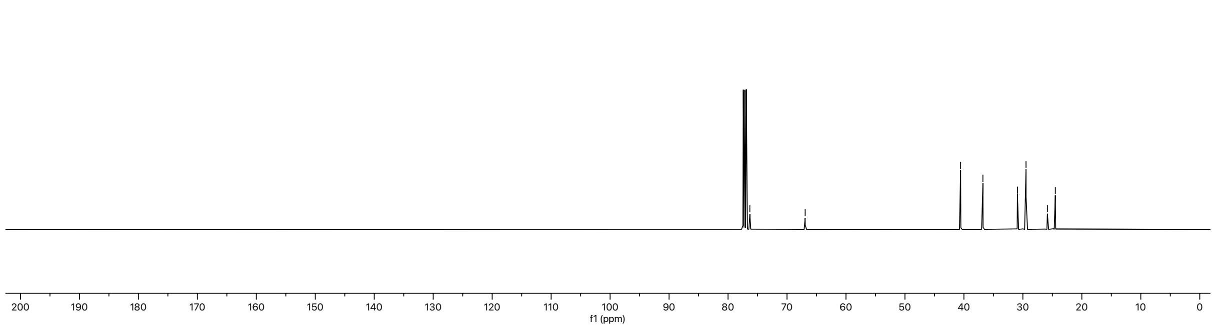

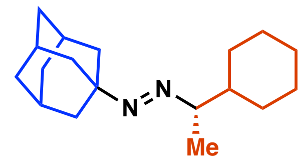

12

(^{1}H, 400 MHz, $CDCl_3$)

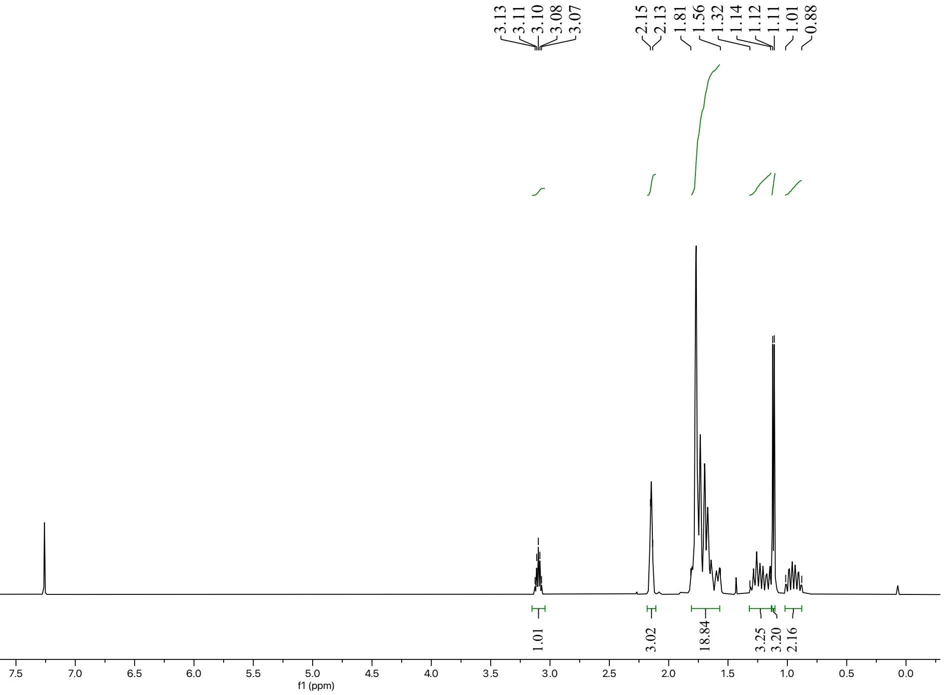

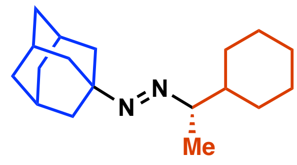

12

(^{13}C, 126 MHz, $CDCl_3$)

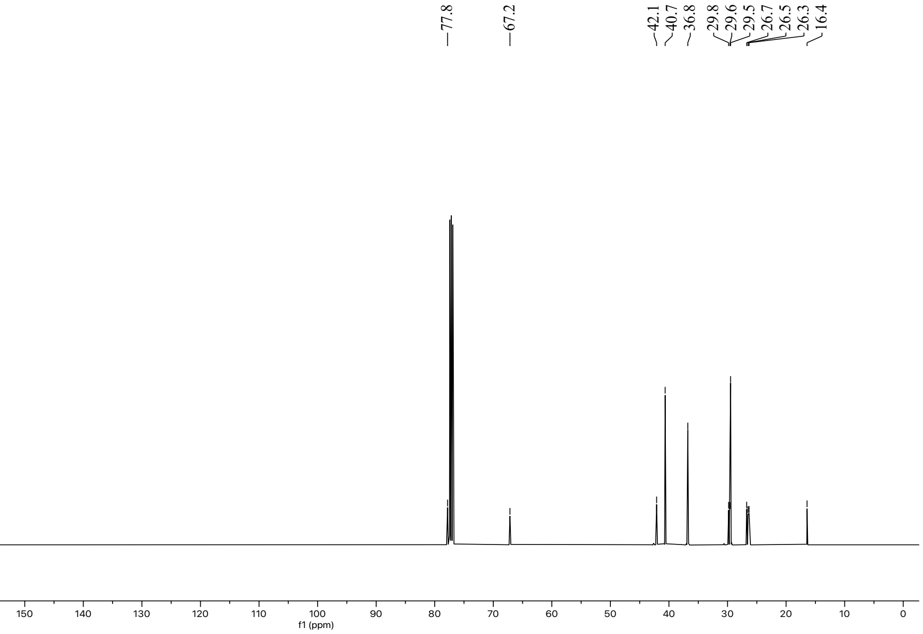

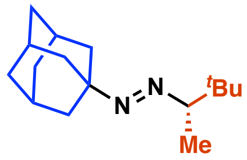

13

(^{1}H, 400 MHz, $CDCl_3$)

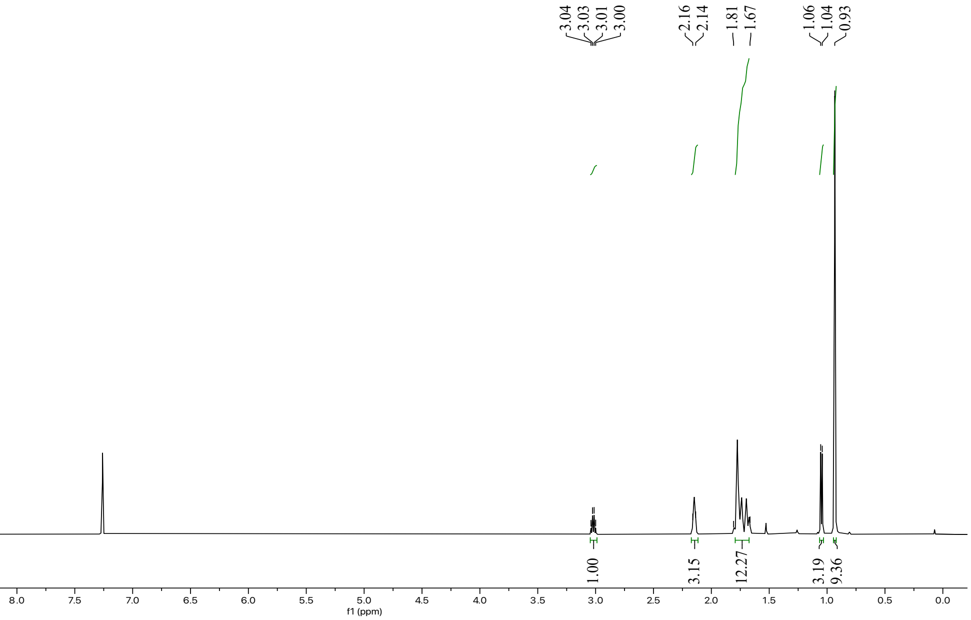

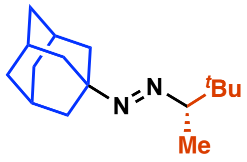

13

(^{13}C, 126 MHz, $CDCl_3$)

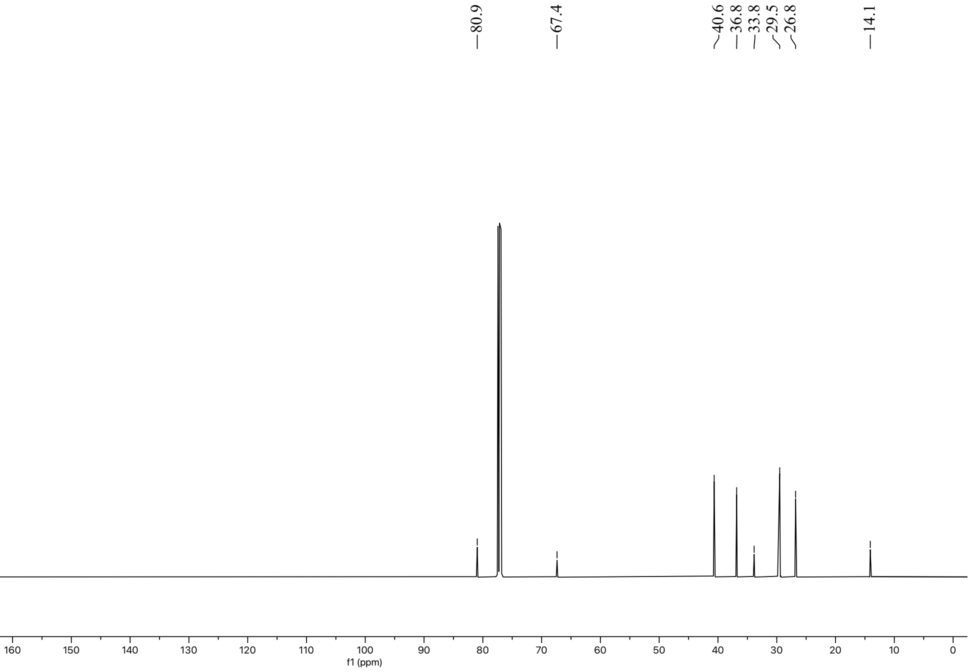

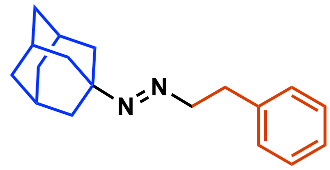

14

(^{1}H, 400 MHz, $CDCl_3$)

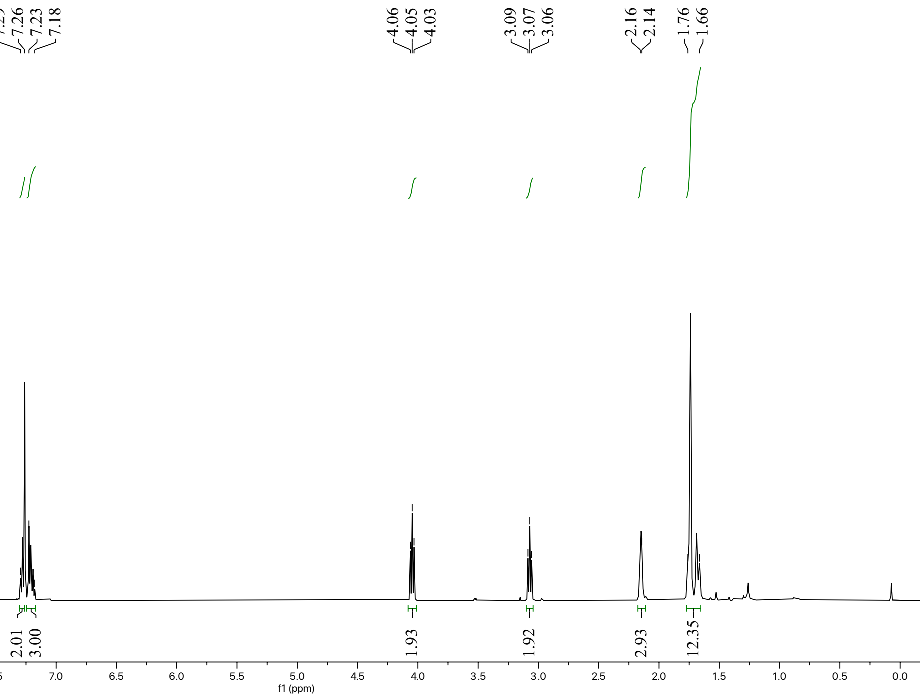

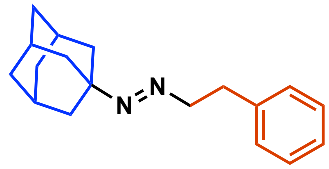

14

(^{13}C, 126 MHz, $CDCl_3$)

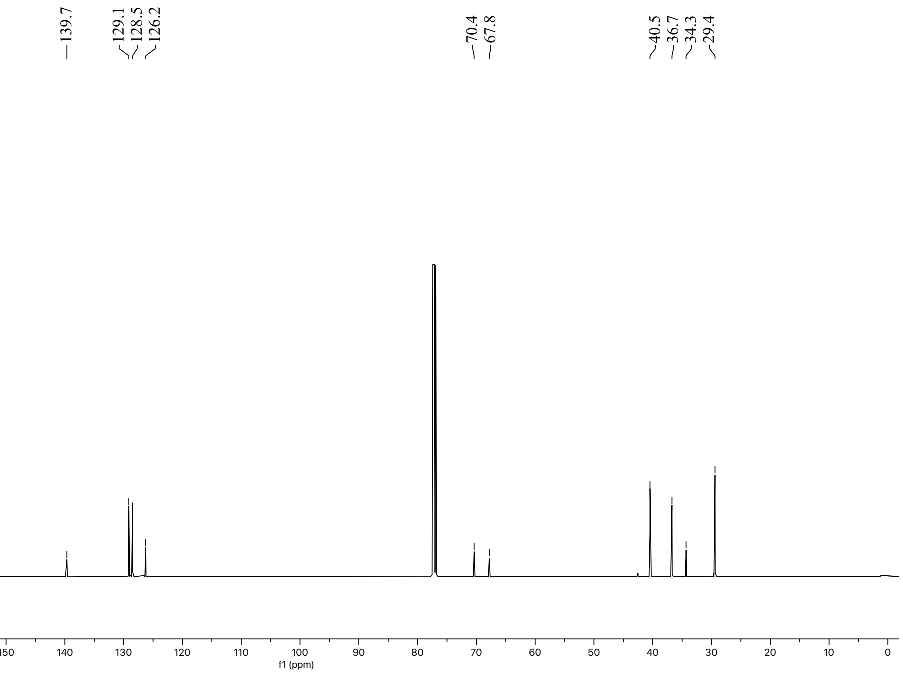

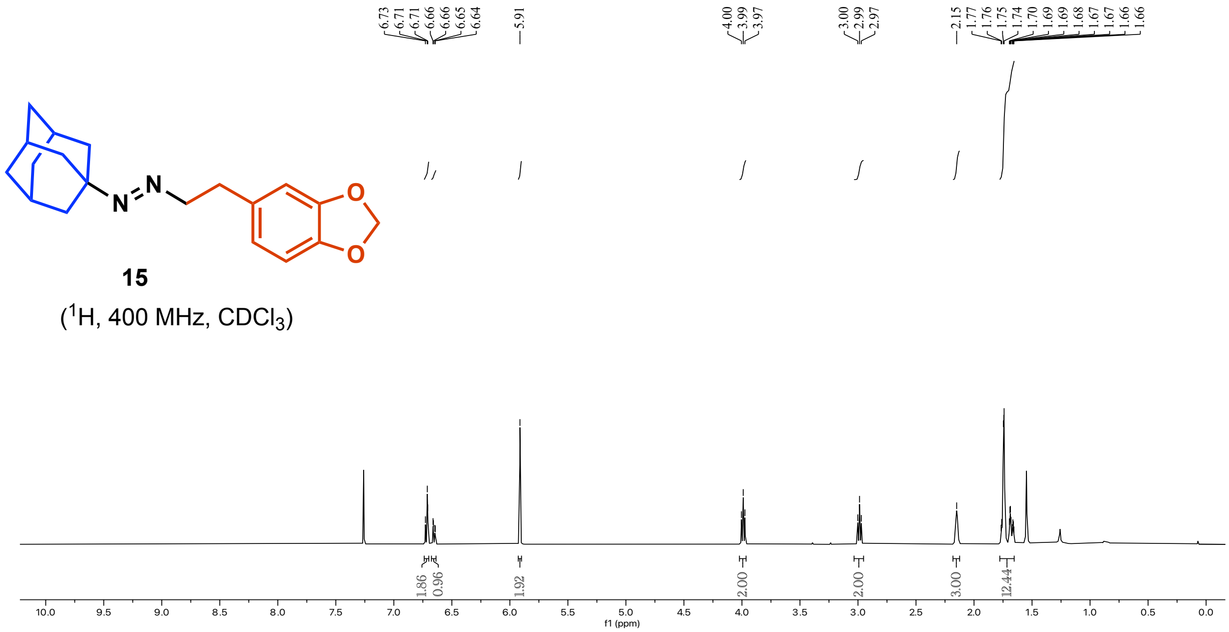

15

(^{1}H, 400 MHz, $CDCl_3$)

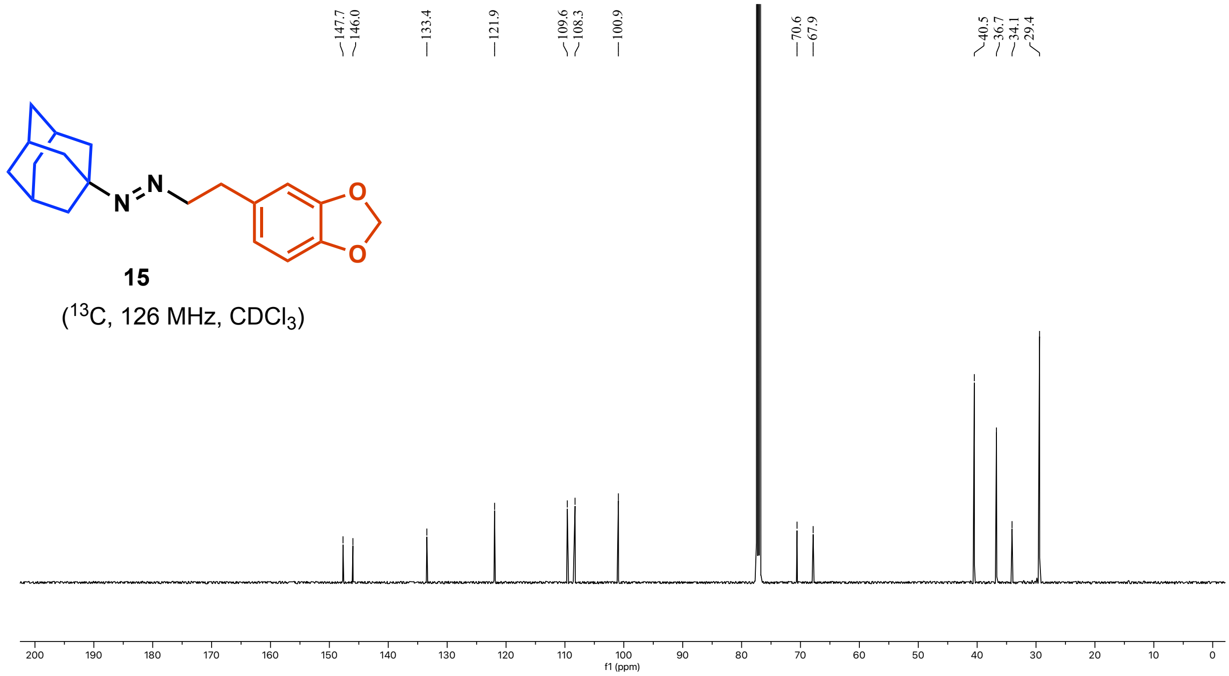

15

(^{13}C, 126 MHz, $CDCl_3$)

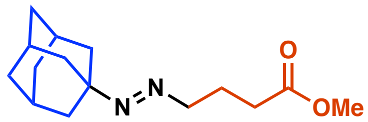

16

(^{1}H, 400 MHz, $CDCl_3$)

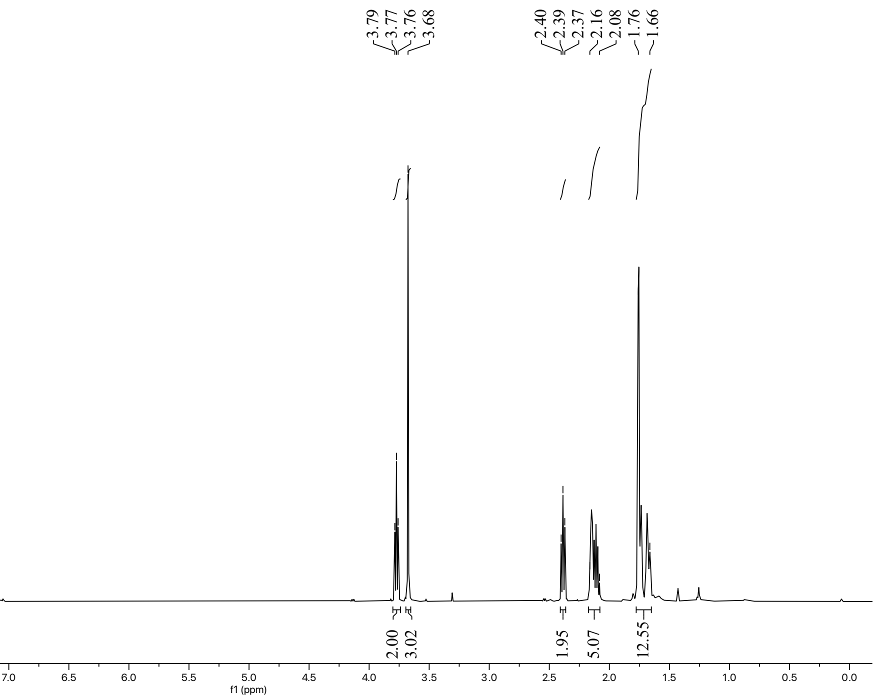

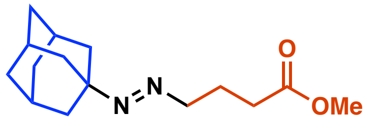

16

(^{13}C, 126 MHz, $CDCl_3$)

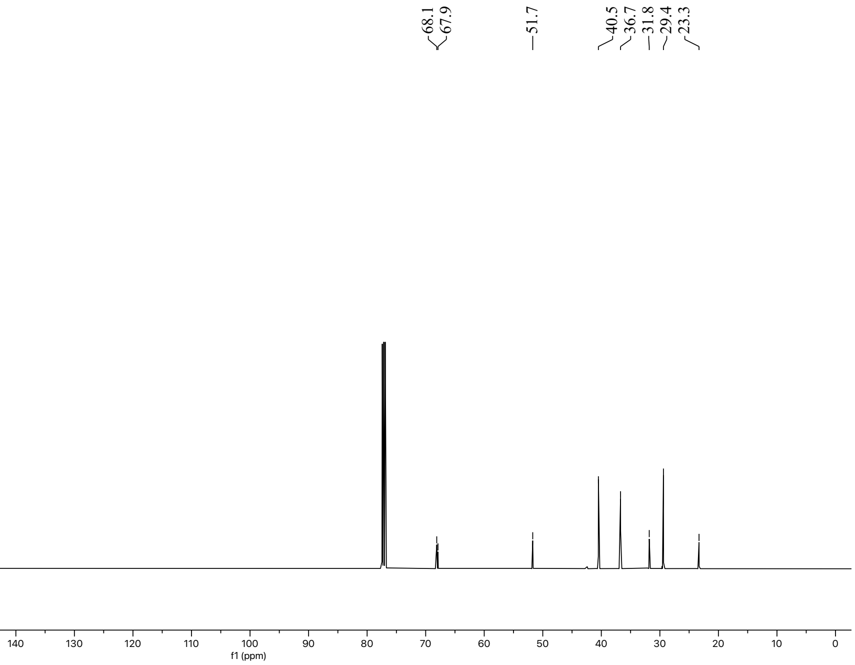

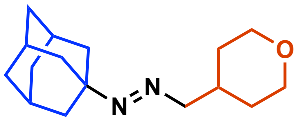

17

(^{1}H, 400 MHz, $CDCl_3$)

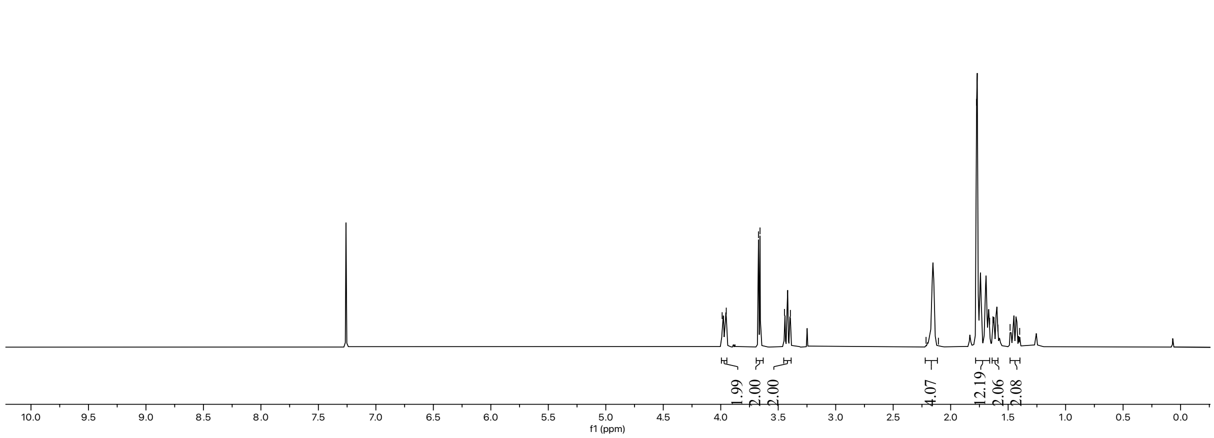

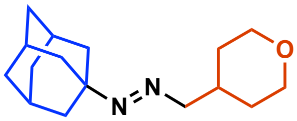

17

(^{13}C, 126 MHz, $CDCl_3$)

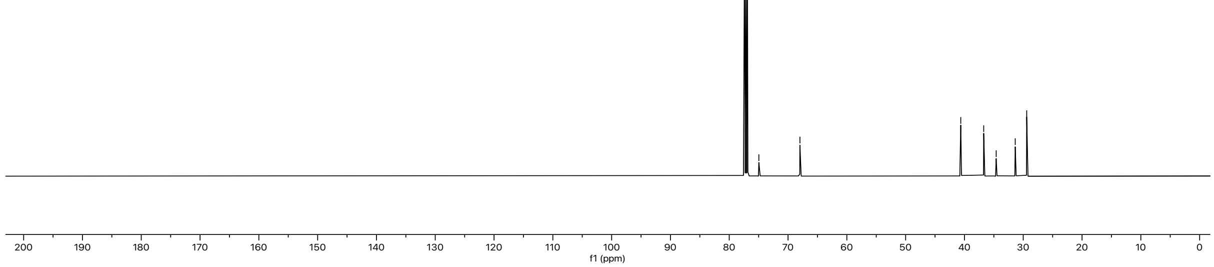

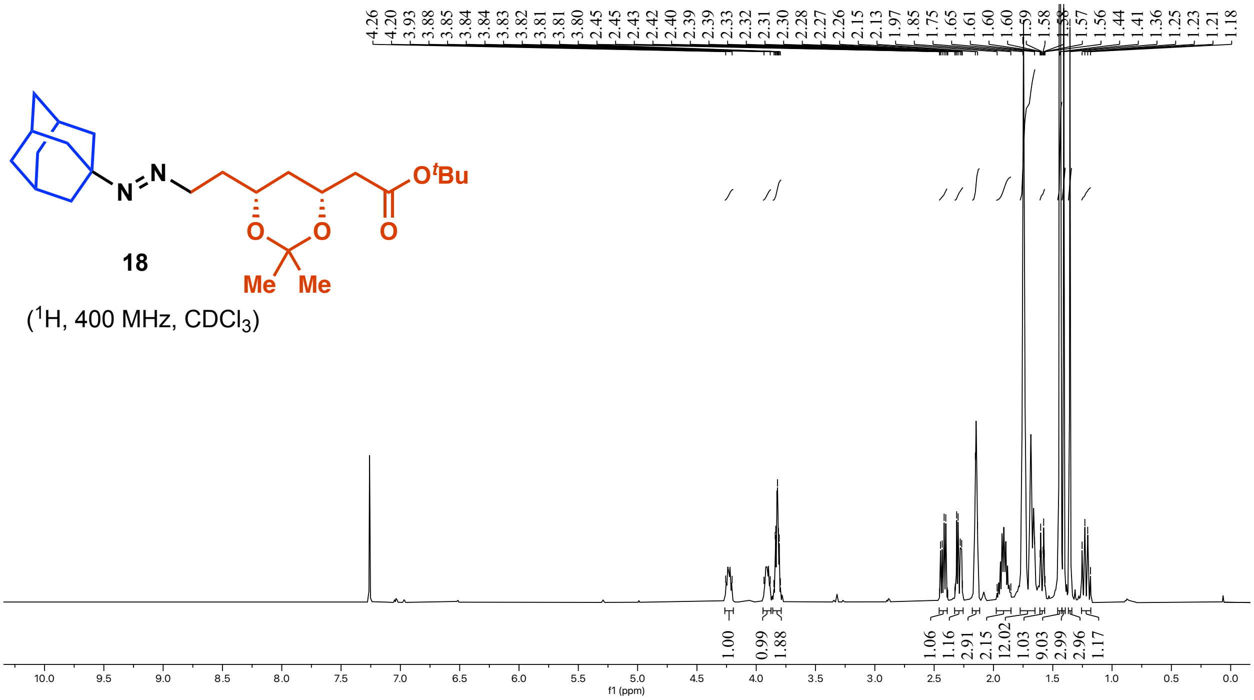

N
N
O^{t}Bu
O
O
O
Me
Me
18
(^{1}H, 400 MHz, CDCl$_3$)
4.26 4.20 3.93 3.88 3.85 3.84 3.84 3.83 3.82 3.81 3.81 3.80 2.45 2.45 2.43 2.42 2.40 2.39 2.39 2.33 2.32 2.31 2.30 2.28 2.27 2.26 2.15 2.13 1.97 1.85 1.75 1.65 1.61 1.60 1.60 1.59 1.58 1.58 1.57 1.56 1.44 1.41 1.36 1.25 1.23 1.21 1.18
1.00 0.99 1.88 1.06 1.16 2.91 2.15 12.02 1.03 9.03 2.99 2.96 1.17
f1 (ppm)

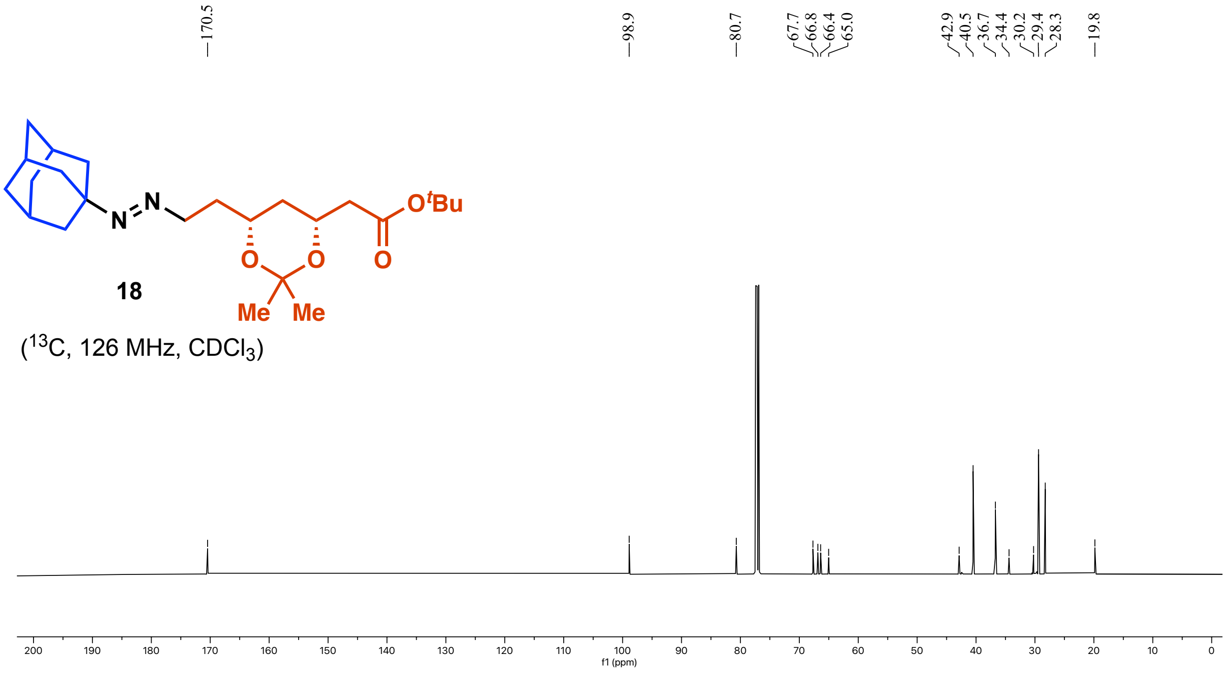

N
N
O^{t}Bu
O
O
O
Me
Me
18
(^{13}C, 126 MHz, CDCl$_3$)
170.5
98.9
80.7
67.7 66.8 66.4 65.0
42.9 40.5 36.7 34.4 30.2 29.4 28.3
19.8
f1 (ppm)

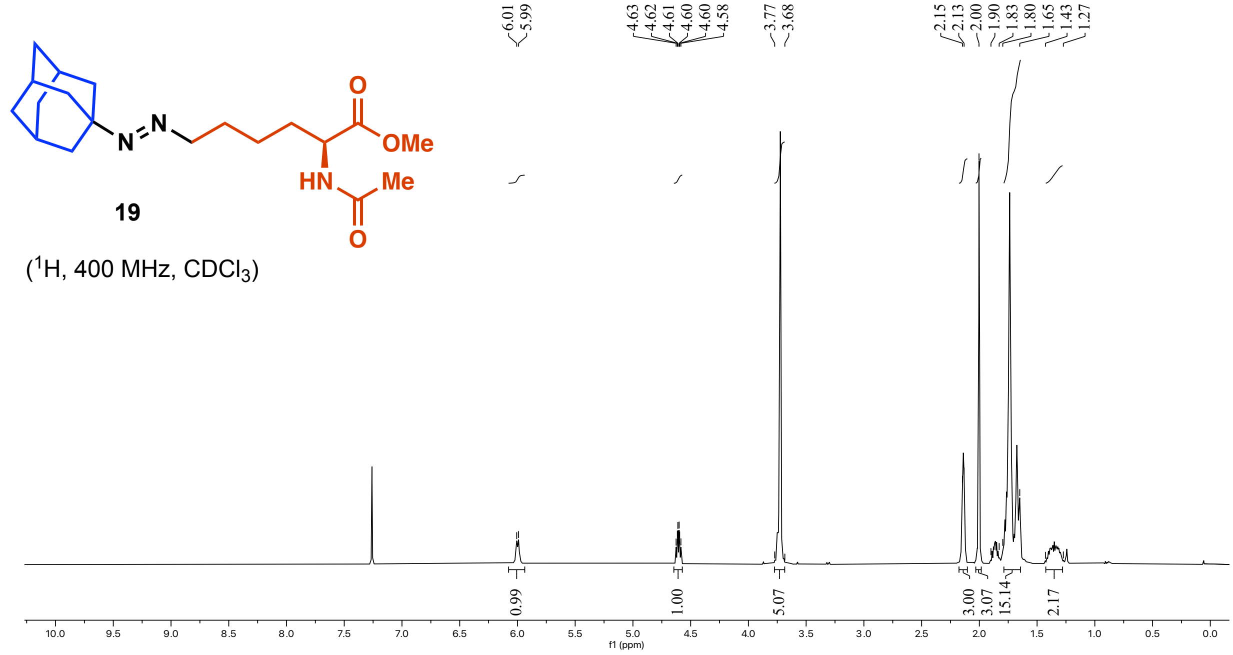
19
(¹H, 400 MHz, CDCl₃)
6.01
5.99
4.63
4.62
4.61
4.60
4.60
4.58
3.77
3.68
2.15
2.13
2.00
1.90
1.83
1.80
1.65
1.43
1.27
0.99
1.00
5.07
3.00
3.07
15.14
2.17
f1 (ppm)

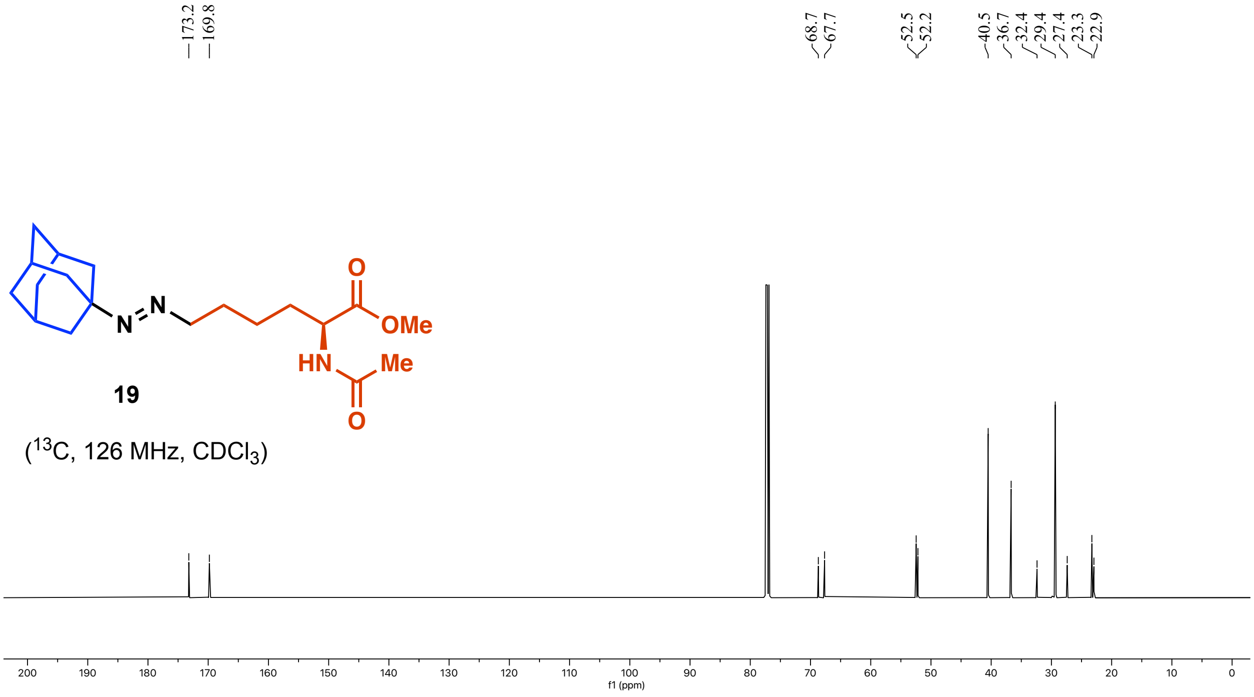
19
(¹³C, 126 MHz, CDCl₃)
173.2
169.8
68.7
67.7
52.5
52.2
40.5
36.7
32.4
29.4
27.4
23.3
22.9
f1 (ppm)

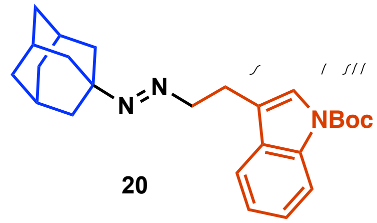

20

(^{1}H, 400 MHz, $CDCl_3$)

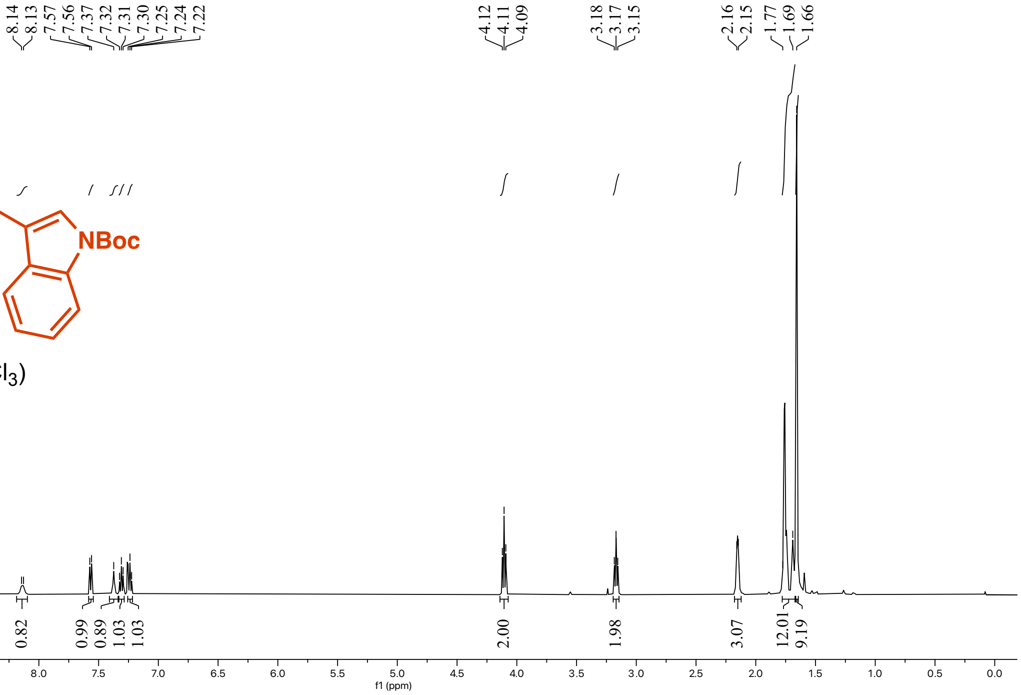

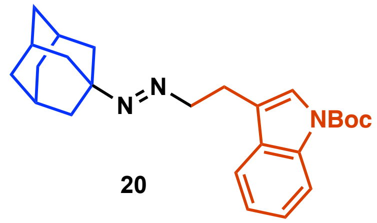

20

(^{13}C, 126 MHz, $CDCl_3$)

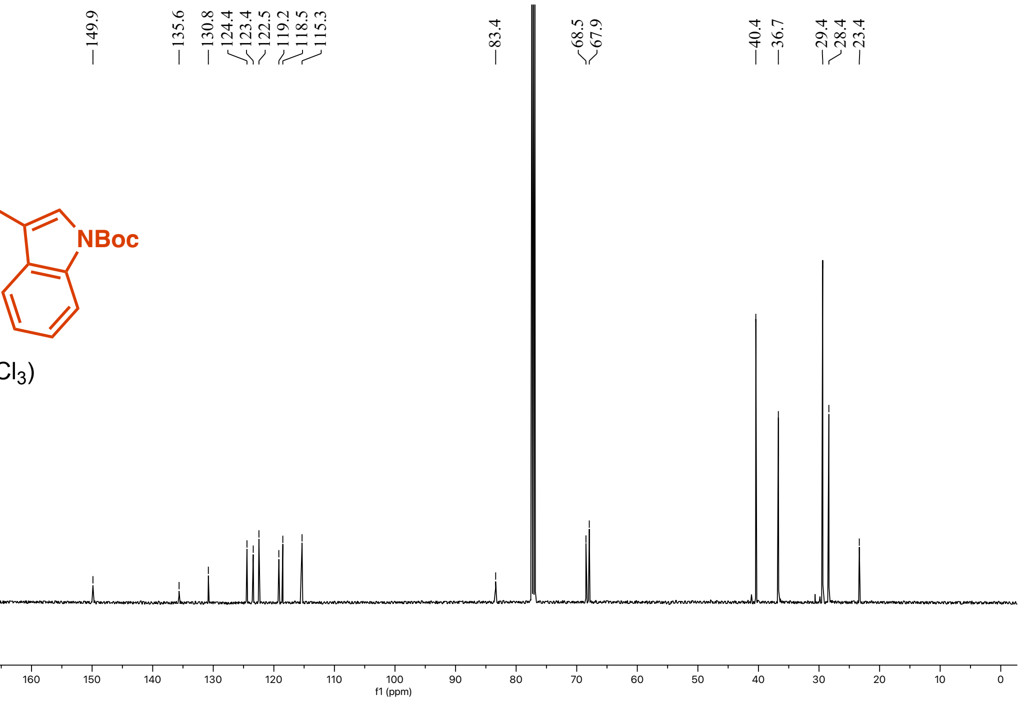

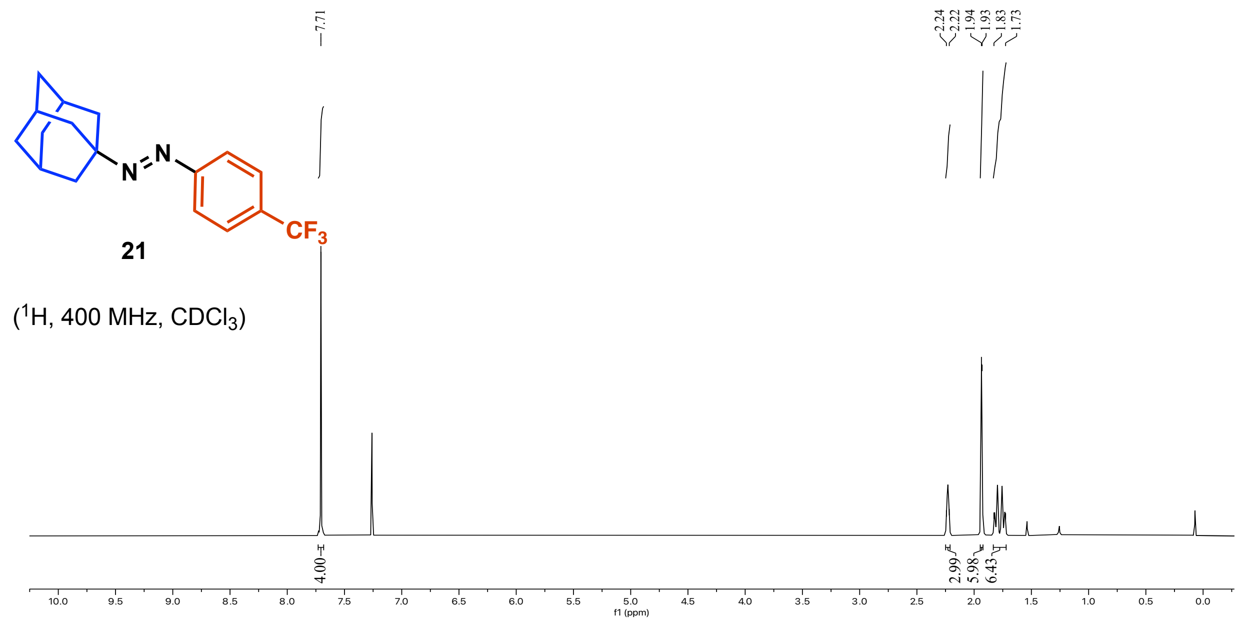

21

(^{1}H, 400 MHz, $CDCl_3$)

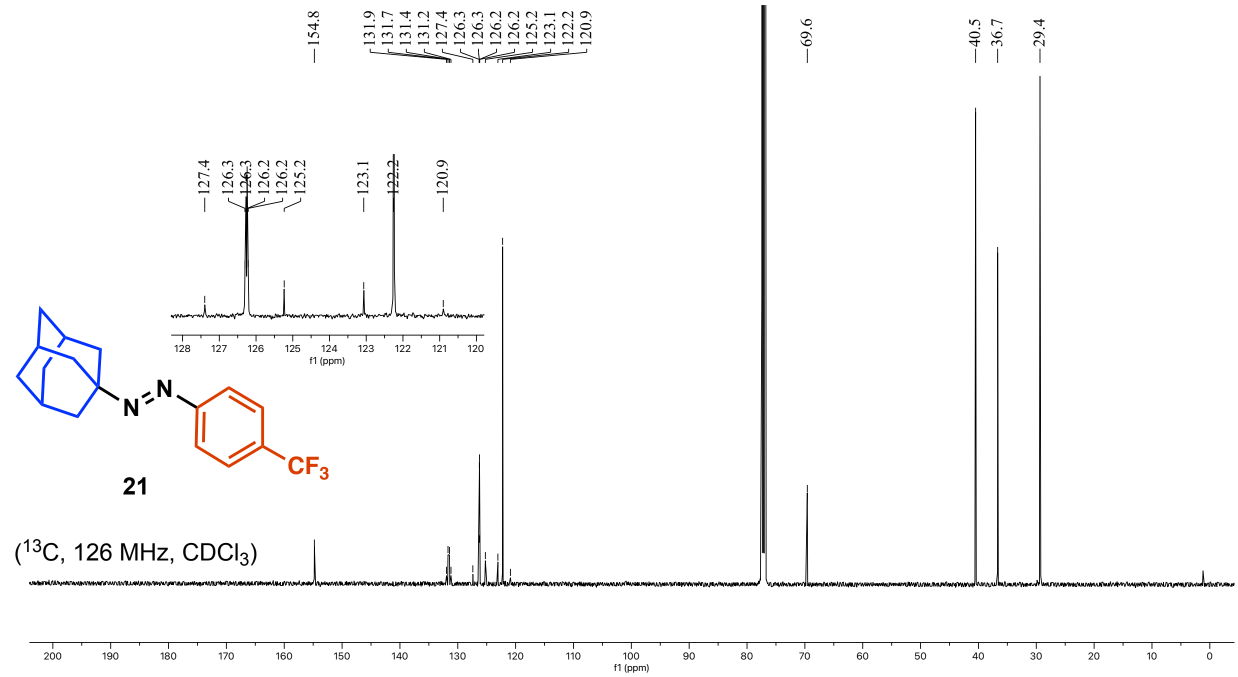

21

(^{13}C, 126 MHz, $CDCl_3$)

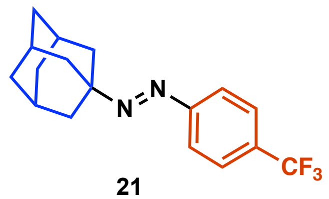

21

(^{19}F, 470 MHz, $CDCl_3$)

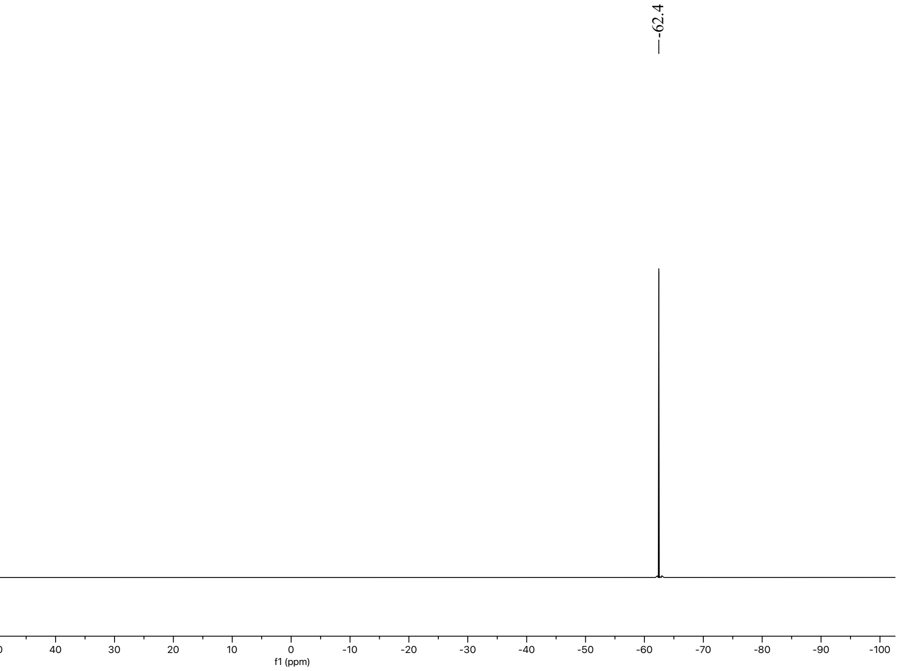

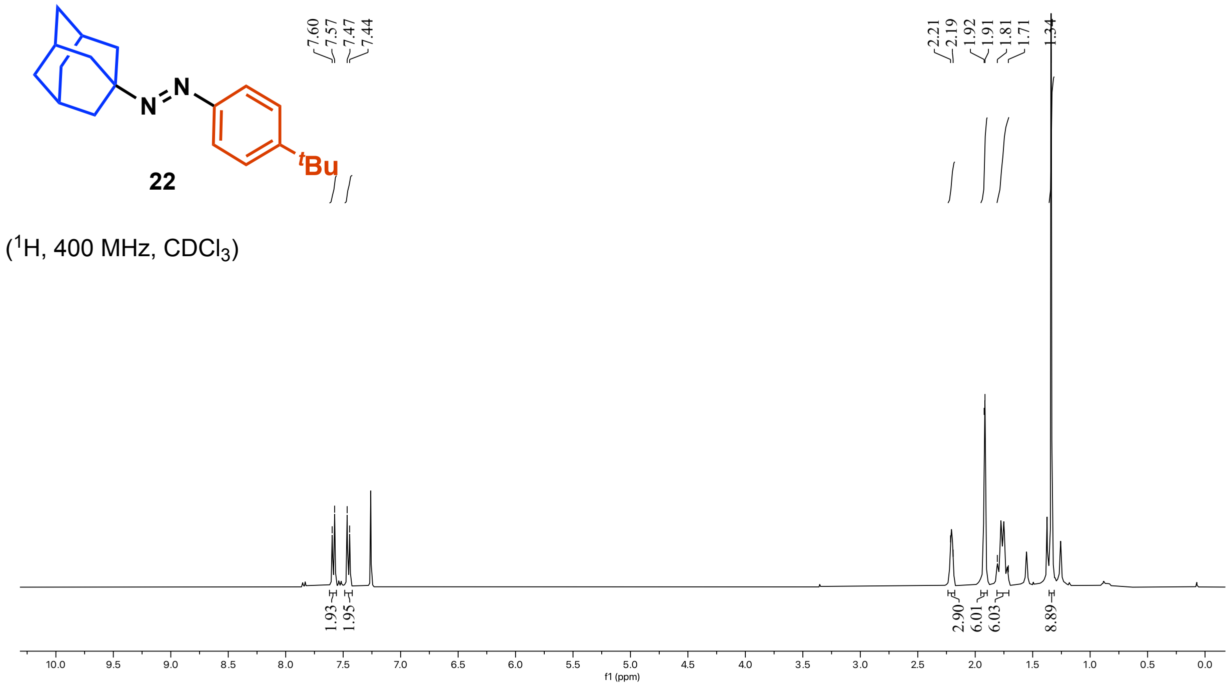

(1H, 400 MHz, $CDCl_3$)

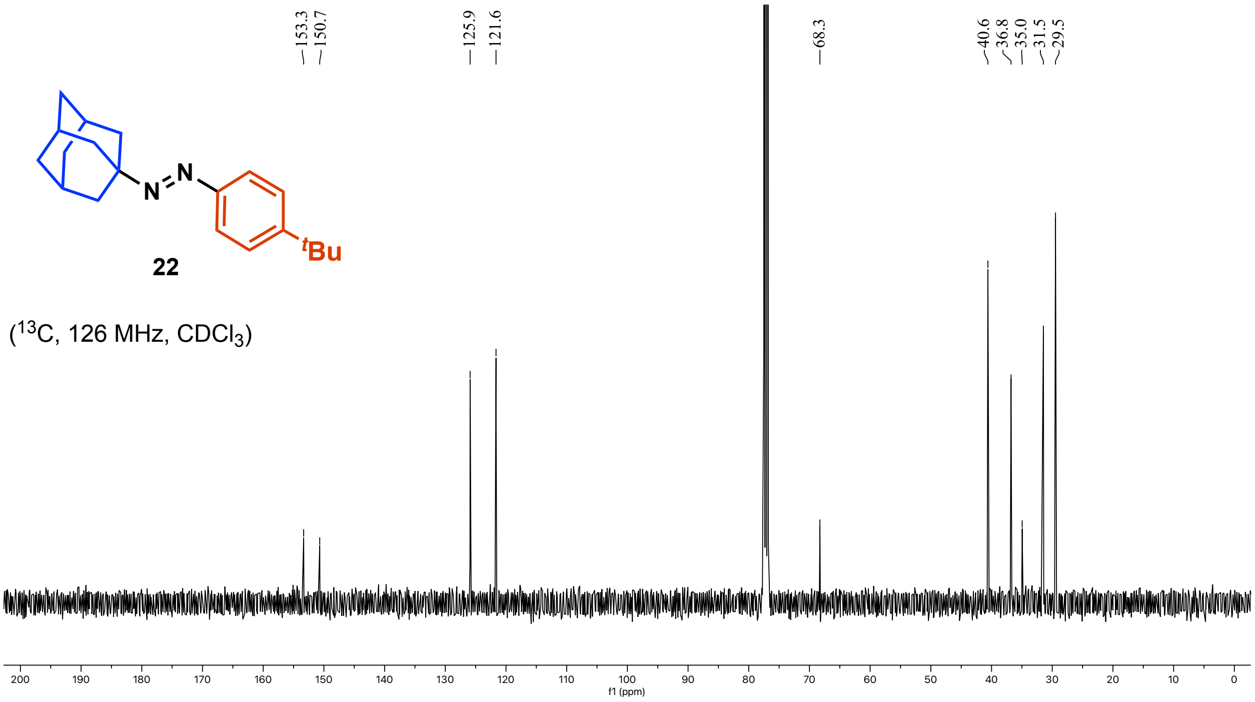

(13C, 126 MHz, $CDCl_3$)

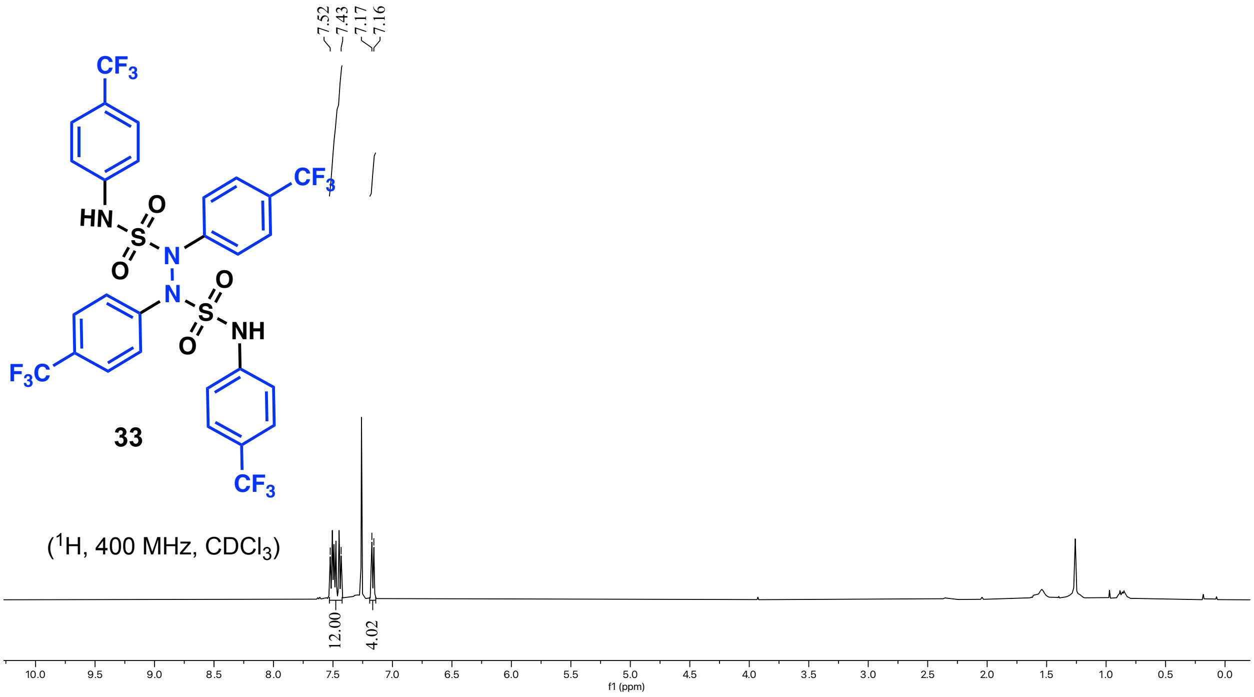
7.52
7.43
7.17
7.16
CF3
HN
O
S
O
N
N
CF3
O
S
O
NH
F3C
33
CF3
(1H, 400 MHz, CDCl3)
12.00
4.02
f1 (ppm)

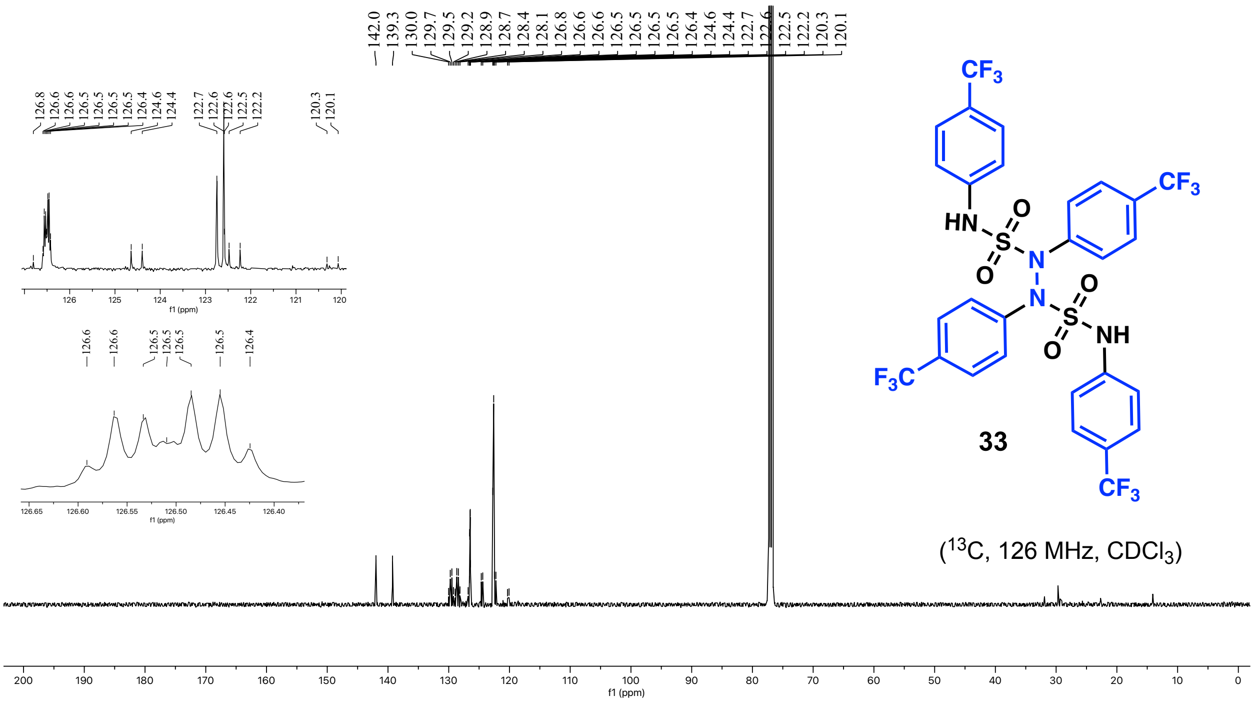
142.0
139.3
130.0
129.7
129.5
129.2
128.9
128.7
128.4
128.1
126.8
126.6
126.6
126.5
126.5
126.5
126.4
124.6
124.4
122.7
122.6
122.5
122.2
120.3
120.1
CF3
HN
O
S
O
N
N
CF3
O
S
O
NH
F3C
33
CF3
(13C, 126 MHz, CDCl3)
f1 (ppm)

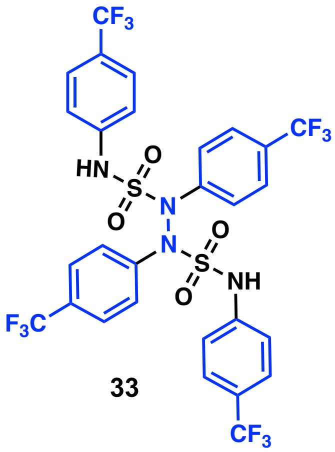

33

(^{19}F, 470 MHz, $CDCl_3$)

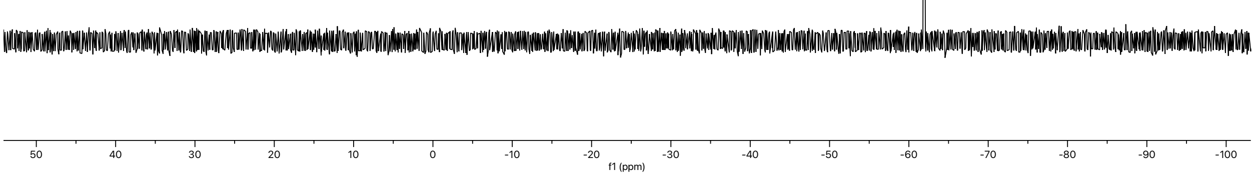

Supplement: Supplementary file 1 — ol4c02218_si_001.pdf [file ol4c02218_si_001.pdf]
